# Multicomponent Synthesis of the SARS-CoV-2 Main Protease Inhibitor Nirmatrelvir

**DOI:** 10.1021/acs.joc.3c01274

**Published:** 2023-08-22

**Authors:** H. Daniel Preschel, Ruben T. Otte, Ying Zhuo, Rebecca E. Ruscoe, Ashleigh J. Burke, Rachel Kellerhals, Brendan Horst, Sven Hennig, Elwin Janssen, Anthony P. Green, Nicholas J. Turner, Eelco Ruijter

**Affiliations:** † Department of Chemistry & Pharmaceutical Sciences, Amsterdam Institute of Molecular & Life Sciences (AIMMS), Vrije Universiteit Amsterdam, De Boelelaan 1108, 1081 HZ Amsterdam, The Netherlands; ‡ Department of Chemistry, The University of Manchester, Manchester Institute of Biotechnology, 131 Princess Street, Manchester M1 7DN, United Kingdom

## Abstract

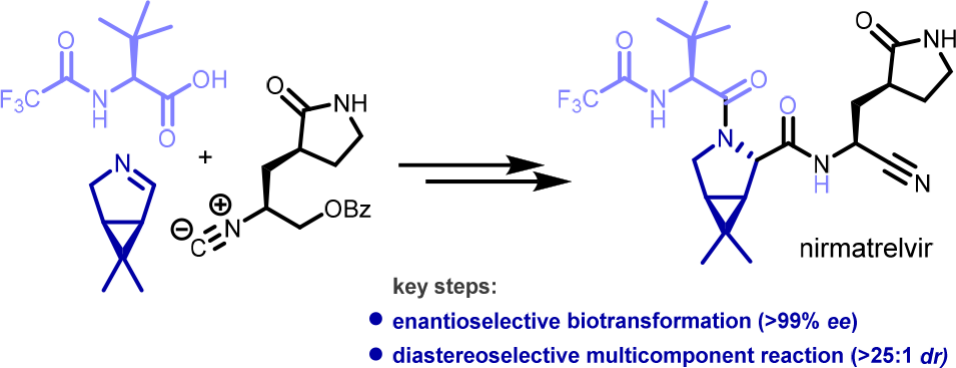

In the wake of the Covid-19 pandemic, it has become clear that global access to efficacious antiviral drugs will be critical to combat future outbreaks of SARS-CoV-2 or related viruses. The orally available SARS-CoV-2 main protease inhibitor nirmatrelvir has proven an effective treatment option for Covid-19, especially in compromised patients. We report a new synthesis of nirmatrelvir featuring a highly enantioselective biocatalytic desymmetrization (>99% ee) and a highly diastereoselective multicomponent reaction (>25:1 dr) as the key steps. Our route avoids the use of transition metals and peptide coupling reagents, resulting in an overall highly efficient and atom-economic process.

## Introduction

The Covid-19 pandemic has impacted global health, society, and economies in a way unprecedented in modern history. Although large-scale vaccination against SARS-CoV-2 has brought the pandemic under control, serious concerns over the rise of new virus variants potentially evading an immune response remain. Consequently, there is an urgent need for orthogonal strategies to prevent or cure SARS-CoV-2 infection. One of the most promising therapeutic interventions to treat active infections with SARS-CoV-2, as well as related viruses that may emerge in the future, is the development of efficacious small molecule drugs.

Among the nonstructural proteins encoded in the SARS-CoV-2 viral genome, the 3C-like protease (3CL^pro^) or main protease (M^pro^) may be regarded as the most druggable target for the effective treatment of Covid-19.^1^ Based on earlier work on the homologous SARS-CoV main protease, multiple highly active mechanism-based inhibitors were recently developed.^2^ The development of the orally available reversible covalent SARS-CoV-2 M^pro^ inhibitor nirmatrelvir (**1**) by Pfizer was a major breakthrough in this area.^3^ In December 2021, the FDA approved Paxlovid (nirmatrelvir + ritonavir) for the emergency treatment of Covid-19 in the US. Regulatory approval in other countries (including the EU and the UK) soon followed.

Our groups share a common interest in developing efficient and sustainable methods for the production of complex small molecule drugs. Previously, we exploited our combined expertise in biocatalysis and multicomponent chemistry in the efficient synthesis of druglike prolyl peptides. Biocatalytic oxidation of *meso*-pyrrolidines with the monoamine oxidase N (MAO-N) D5 mutant afforded the corresponding bicyclic imines,^4^ which were subjected to a diastereoselective Ugi-type three-component reaction (U-3CR)^5^ to give bicyclic proline derivatives in high optical purity.^6^ This strategy was subsequently exploited in an efficient and diastereoselective synthesis of the hepatitis C virus (HCV) NS3 protease inhibitor, telaprevir.^7^

**Scheme 1:**
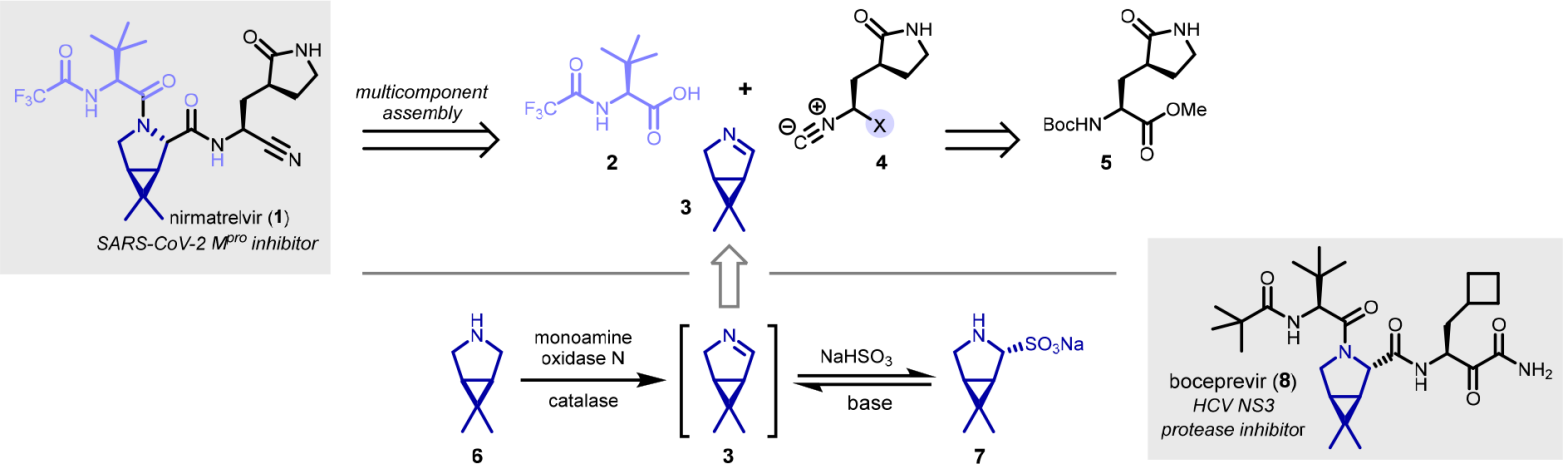
Retrosynthesis of Nirmatrelvir and Biocatalytic Synthesis of Imine **3**

With the emergence of new, highly active SARS-CoV-2 M^pro^ inhibitors featuring bicyclic proline residues,^2d,3^ we recognized these compounds may be accessed using a similar strategy. In particular, we envisioned the rapid multicomponent assembly of nirmatrelvir from *N*-trifluoacetyl-*tert*-leucine (**2**), chiral bicyclic imine **3**, and isocyanide **4** (Scheme 1). The latter could plausibly be derived from the known compound **5**.^8^ We anticipated that this U-3CR would proceed with high diastereoselectivity owing to highly effective shielding of the concave face of the bicyclic system of **3** by the *gem*-dimethyl moiety. We had previously demonstrated that the stereochemical outcome of such U-3CRs is determined solely by the chiral bicyclic imine, and not by chiral isocyanides or carboxylic acids.^6^ Biocatalytic access to imine **3** by MAO-N-mediated oxidation of **6** was previously reported by Merck and Codexis researchers, who also demonstrated it could be conveniently isolated as its crystalline bisulfite adduct **7** (Scheme 1).^9^ This method was optimized for the large-scale production of **7** as an intermediate toward the HCV NS3 protease inhibitor, boceprevir (**8**), which features the same bicyclic proline residue as nirmatrelvir. Given the excellent accessibility of imine **3**, we decided to pursue the multicomponent assembly of nirmatrelvir by U-3CR (Scheme 1) as a potentially greener alternative to the original route^3^ (as well as other, related routes^10,11^) that rely heavily on peptide coupling of the respective protected amino acids (see Supporting Information (SI) Scheme S1 and Table S1 for a direct comparison of our route and the original route).

## Results and Discussion

The bisulfite adduct **7** of the chiral imine **3** was prepared by monoamine oxidase (MAO-N) catalyzed oxidation of the amine **6** according to the published procedure.^9^ The enantioselectivity of the MAO-N-mediated formation of **3** (>99% ee) was determined by reaction of the cyclic imine **3** with phenylmagnesium bromide to yield the corresponding Grignard adduct, which was compared to a racemic standard (see the SI for further details).

Having secured access to key imine derivative **7**, we next focused on the synthesis of requisite isocyanide **4**. While it is widely accepted that the Ugi reaction and its variations typically have a very broad scope with respect to the isocyanide, there are only scarce reports of the use of chiral, highly functionalized isocyanides such as **4**.^12^ We have previously established that the generation of the isocyanide moiety from the corresponding formamide is compatible with the presence of esters and secondary amides.^7^ However, we soon ruled out the use of esters and nitriles as the X substituent in **4**, as such α-acidic isocyanides typically display rather different reactivity.^13^ Moreover, α-isocyano esters tend to be configurationally unstable, while the near-complete absence of α-isocyano nitriles in the literature suggests they are inherently unstable.^14^ We thus decided to carry forward the C-terminus in a reduced form, i.e., as a protected primary alcohol. Given the demonstrated compatibility of esters with both isocyanide generation and the multicomponent reaction,^7^ we selected **4a** (X = CH_2_OBz) as the target isocyanide, with the benzoate facilitating TLC and SFC analysis.

**Scheme 2:**
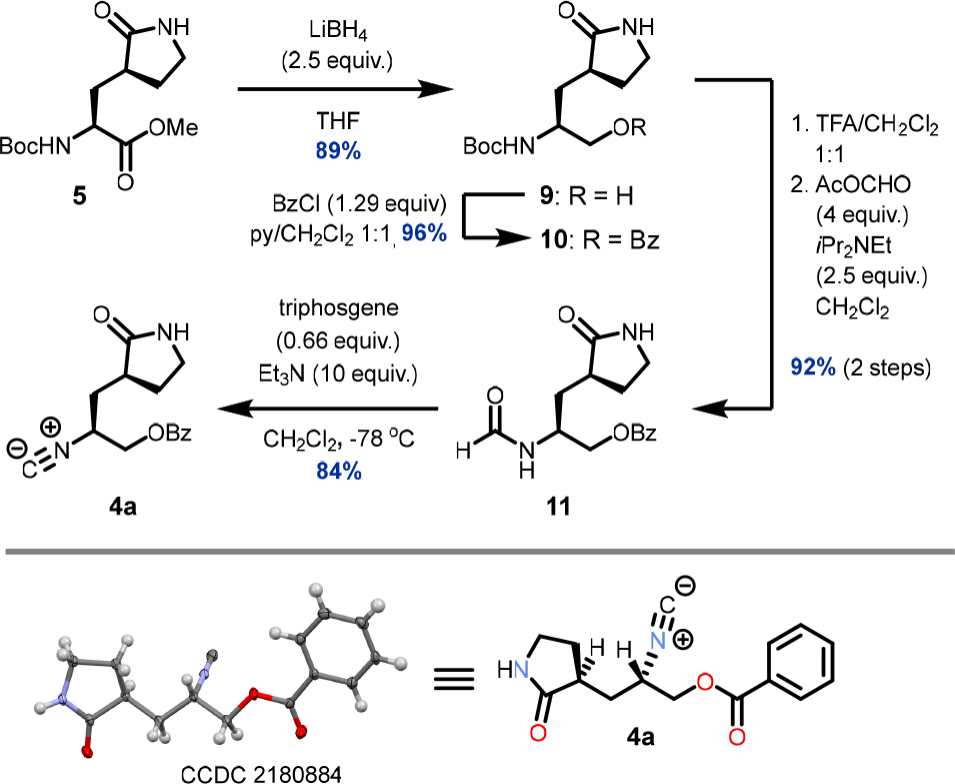
Synthesis of Isocyanide **4a**

The Boc-protected amino ester **5** has been reported by several sources^8^ and is now also commercially available. Despite widely recognized reproducibility issues^8c^ with the key cyanomethylation step,^15^ we were able to obtain sufficient amounts of **5** following literature procedures.^8a,8b^ We then turned our attention to the synthesis of isocyanide **4a** (Scheme 2). Chemoselective reduction of the ester moiety followed by benzoylation smoothly afforded **10**. Subsequent Boc cleavage followed by immediate formylation gave formamide **11** in excellent yield. The dehydration of **11** to **4a** initially proved challenging, with most initial attempts leading either to incomplete conversion or to decomposition. Gratifyingly, under optimized conditions [triphosgene (0.66 equiv), Et_3_N (10 equiv), CH_2_Cl_2_, −78 °C] we could obtain **4a** in 84% isolated yield. Alternatively, **11** can be dehydrated to give **4a** in 83% isolated yield by treatment with trifluoroacetic anhydride (1.9 equiv) and Et_3_N (8 equiv) in THF (0 °C, 30 min.).^16^ To our delight, isocyanide **4a** also proved to be a readily isolable, stable, crystalline solid. We were able to grow suitable crystals of **4a** for X-ray diffraction and thus unambiguously confirmed its structure and absolute configuration (Scheme 2).

**Scheme 3:**
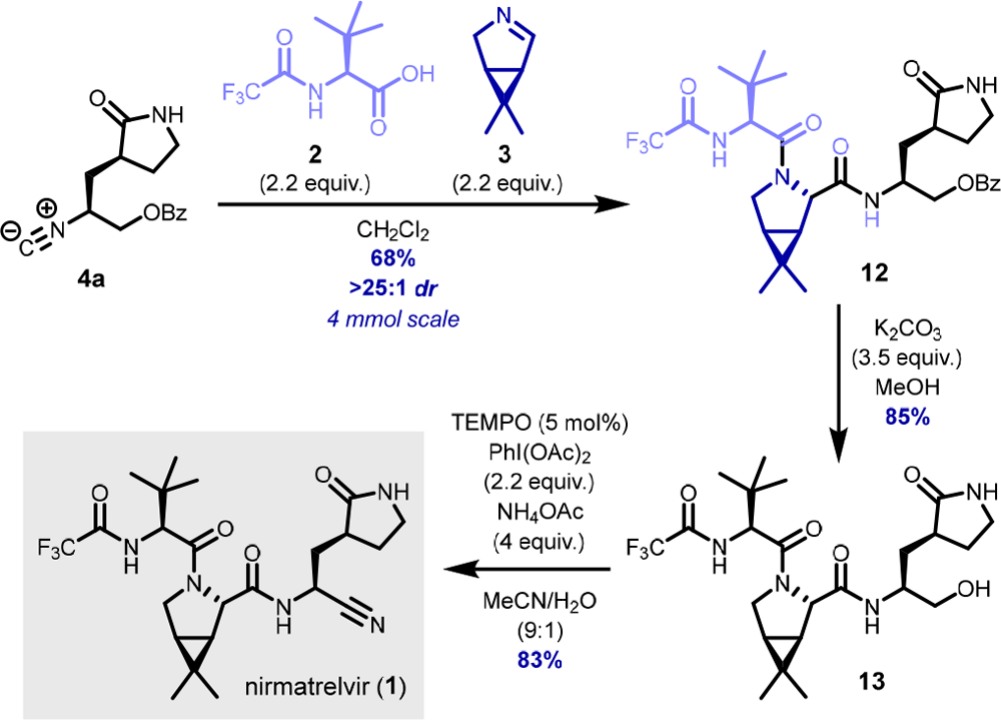
Synthesis of Nirmatrelvir

With all required fragments in hand, we moved on to the multicomponent assembly of the nirmatrelvir core. Initially, we attempted to use bisulfite adduct **7** directly in the U-3CR using model isocyanides and carboxylic acids as the other inputs. Although we were indeed able to isolate the corresponding Ugi adducts when the reaction was performed in protic solvents such as MeOH, the reaction was sluggish (plausibly as a result of the slow equilibrium between **7** and **3**) and the isolated yield was low (∼20–35%). Moreover, we observed the formation of the “truncated” Ugi products (i.e., lacking the carboxylic acid moiety) as a side reaction.^17^ We therefore opted to perform the key U-3CR with free imine **3** (Scheme 3). With a moderate excess of carboxylic acid **2** and the volatile, *in situ* generated imine **3**, the U-3CR smoothly proceeded to completion (i.e., with full consumption of isocyanide **4a**) to give Ugi adduct **12** in 68% isolated yield. In our initial efforts, **12** was isolated as an ∼5:1 mixture of diastereomers (as determined by SFC-MS analysis), which we initially attributed to incomplete stereoinduction at the newly formed stereocenter. Upon closer examination, however, we found that the procedure we used for the trifluoroacetylation of *tert-*leucine^18^ led to some erosion of the stereochemistry, affording **2** in varying ee. Gratifyingly, the use of commercial **2** (>98% ee) in the key U-3CR afforded **12** in good yield with excellent diastereoselectivity (>25:1 dr). Subsequent methanolysis of the benzoate ester then gave alcohol **13** in a good yield. To our delight, oxidative conversion of the primary alcohol in **13** to the nitrile was smoothly accomplished using a convenient one-pot procedure^19^ combining PhI(OAc)_2_/TEMPO oxidation with ammonium acetate as the nitrogen source to afford nirmatrelvir in 83% yield, with NMR data corresponding with literature data.^3^

**Scheme 4:**
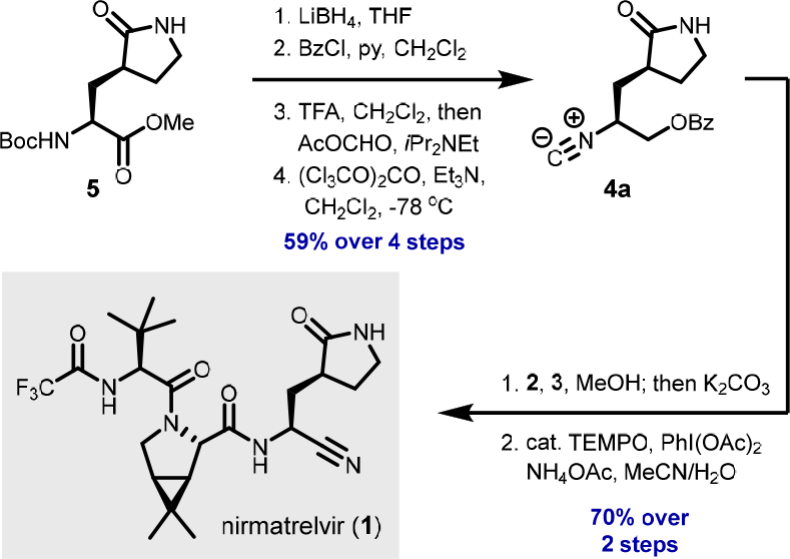
Streamlined Route to Nirmatrelvir

Encouraged by the smooth conversion of essentially all of the steps in our synthesis, we investigated whether we could reduce the number of intermediate purifications (Scheme 4). After streamlining some workup procedures, we were able to carry forward commercial ester **5** to isocyanide **4a** without intermediate purifications with only minimal yield loss, affording **4a** in 59% yield over four steps (versus 66% combined yield over the four individual steps). Advantageously, the subsequent U-3CR of **2**, **3**, and **4a** can also be performed in MeOH, allowing the one-pot combination with the ensuing saponification to give **13** with >25:1 dr. The crude product **13** was then directly used in the final step, affording nirmatrelvir in 70% yield from **4a**, which is considerably higher than the yield over the last three steps with intermediate purifications (48%, cf. Scheme 3).

## Conclusion

We developed a new synthetic route to the SARS-CoV-2 M^pro^ inhibitor nirmatrelvir featuring a biocatalytic desymmetrization and construction of the central bicyclic proline residue by an Ugi-type multicomponent reaction as the key steps. The process is scalable, efficient, and highly selective owing to the excellent enantioselectivity of the biotransformation (>99% ee) and excellent diastereoselectivity of the multicomponent reaction (>25:1 dr). Nearly all of the steps in the process proceed with full conversion, reducing the necessity for intermediate purifications. Despite a required reduction/oxidation sequence, our route compares favorably to the original route in terms of step count and overall yield (SI Scheme S1 and Table S1). Combined, these credentials provide a basis for future development of the route to a possibly more sustainable and cost-efficient production process of this valuable API.

## Experimental Section

### General Information

Compounds **2** and **5** were purchased from Angene Chemical. Other commercially available reagents were purchased from Merck (Sigma-Aldrich), Fischer Scientific, Strem Chemicals, TCI Europe, or Fluorochem and were used as received, unless mentioned otherwise. Solvents were purchased from VWR Chemicals or Sigma-Aldrich and used without purification unless stated otherwise. Anhydrous, air-free solvents (CH_2_Cl_2_, toluene, THF) were obtained from a PureSolv MD 5 solvent purification system. Nuclear magnetic resonance (NMR) spectra were recorded on a Bruker Avance 600, Bruker Avance 500, or Bruker Avance 300 using the residual CHCl_3_ signal (^1^H: δ 7.26 ppm) or the CDCl_3_ signal (^13^C{^1^H}: δ 77.16 ppm) as internal reference standard. Chemical shifts (δ) are given in ppm, and coupling constants (*J*) are quoted in hertz (Hz). Resonances are described as s (singlet), d (doublet), t (triplet), q (quartet), br (broad singlet), and m (multiplet) or combinations thereof. Structural assignments were made with additional information from gCOSY, gHSQC, and gHMBC experiments. Electrospray ionization (ESI) high-resolution mass spectrometry was carried out using a Bruker micrOTOF-Q instrument in positive-ion mode (capillary potential of 4500 V). Flash chromatography was performed on Silicycle Silica-P Flash Silica Gel (particle size 40–63 μm, pore diameter 60 Å) using the indicated eluent. Thin layer chromatography (TLC) was performed using TLC plates from Merck (SiO_2_, Kieselgel 60 F254 neutral, on aluminum with fluorescence indicator), and compounds were visualized by UV detection (254 nm), potassium permanganate, cerium(IV) sulfate, ninhydrin, and/or *p*-anisaldehyde stain. SFC-MS analysis was conducted using a Shimadzu Nexera SFC-MS equipped with a Nexera X2 SIL-30AC autosampler, Nexera UC LC-30AD SF CO_2_ pump, Nexera X2 LC-30AD liquid chromatograph, Nexera UC SFC-30A back pressure regulator, prominence SPD-M20A diode array detector, prominence CTO-20AC column oven, and CBM-20A system controller, coupled to a Shimadzu LCMS-2020 mass spectrometer. The data were acquired in full-scan APCI mode (MS) from *m*/*z* 100 to 800 in positive ionization mode. Data was processed using Shimadzu Labsolutions 5.82. Enantiomeric excess was determined by SFC-MS analysis using method A [Lux 3 μm Cellulose-3 column (cellulose tris(4-methylbenzoate), 150 × 4.6 mm) eluting with an isocratic mixture of supercritical CO_2_ (99%) and methanol (1%) at 1 mL/min, run length of 10 min, detection at 246 nm] or method B [Lux 3 μm Cellulose-1 column (cellulose tris(3,5-dimethylphenylcarbamate) eluting with an isocratic mixture of supercritical CO_2_ (70%) and methanol (30%) at 2 mL/min, run length of 5.5 min, detection at 224 nm]. The sample injection volume was 5 μL. Specific rotations were measured on a Krüss P3000 polarimeter, using a 0.5 dm cell and solvent as indicated. GC-FID analysis was performed on an Agilent 6850 GC with a Gerstel Multipurpose sampler MPS2L using β-dex 325 (Supelco) with dimensions 30 m × 0.25 mm × 0.25 μm. X-ray crystallographic data were obtained from a Bruker D8 Quest instrument, equipped with PHOTON II detector. X-rays were generated from an INCOATEC IμS 3.0 Cu Kα sealed X-ray tube source at 1.54178 Å. Measurements were carried out at 100(2) K, using an Oxford Cryosystems CRYOSTREAM 800.

Experimental details for the biocatalytic production of imine **3** and its bisulfite adduct **7**, additional optimization data for the dehydration of **11** to **4a**, and experimental details for the streamlined synthesis of **1** are reported in the Supporting Information.

#### *tert*-Butyl ((*S*)-1-Hydroxy-3-((*S*)-2-oxopyrrolidin-3-yl)propan-2-yl) Carbamate (**9**)

To a solution of **5**(20) (5.00 g, 17.46 mmol, 1.0 equiv) in dry THF (140 mL) at 0 °C under N_2_ atmosphere LiBH_4_ (2 M in THF, 21.8 mL, 43.6 mmol, 2.5 equiv) was added dropwise. The reaction mixture was kept at 0 °C for 10 min, then allowed to warm to room temperature, and stirred for 3 h. Upon full conversion (as judged by TLC analysis), the reaction mixture was cooled to 0 °C and quenched by dropwise addition of aq NH_4_Cl (50 mL, 0.5 M) until gas evolution ceased. The reaction mixture was further diluted with water (100 mL), and the aqueous phase was repeatedly extracted with ethyl acetate (6 × 150 mL). The combined organic extracts were dried (MgSO_4_), concentrated *in vacuo*, and subjected to flash column chromatography over silica gel (6–8% MeOH in CH_2_Cl_2_) to afford **9** (4.024 g, 15.58 mmol, **89%**) as a white, foamy solid. ***R***_***f***_: 0.35 (6% MeOH in CH_2_Cl_2_; ninhydrin stain). ^**1**^**H NMR (600 MHz, CDCl**_**3**_**)** δ 6.37 (s, 1H), 5.47 (d, *J* = 7.0 Hz, 1H), 3.76–3.67 (m, 1H), 3.61 (dd, *J* = 11.3, 4.2 Hz, 1H), 3.58 (dd, *J* = 11.3, 4.8 Hz, 1H), 3.38–3.29 (m, 2H), 2.58–2.44 (m, 1H), 2.54–2.45 (m, 1H), 2.43–2.32 (m, 1H), 1.94 (ddd, *J* = 14.4, 9.9, 4.5 Hz, 1H), 1.87–1.78 (m, 1H), 1.60 (ddd, *J* = 14.4, 7.5, 4.5 Hz, 1H), 1.42 (s, 9H). ^**13**^**C{**^**1**^**H} NMR (126 MHz, CDCl**_**3**_**)** δ 181.1 (CO), 156.7 (CO), 79.6 (C), 66.1 (CH_2_), 51.2 (CH), 40.6 (CH_2_), 38.2 (CH), 32.6 (CH_2_), 28.5 (CH_3_). **HRMS (ESI)**: *m*/*z* calcd for C_12_H_22_N_2_O_4_Na ([M + Na]^+^) 281.1472, found 281.1477. **Mp**: 161.2 °C, **[α]**_**D**_^**20**^ −16 (*c* = 2.0, CHCl_3_).

#### (*S*)-2-((*tert*-Butoxycarbonyl)amino)-3-((*S*)-2-oxopyrrolidin-3-yl)propyl Benzoate (**10**)

Boc-amino alcohol **9** (586 mg, 2.27 mmol, 1.0 equiv) was dissolved in dry CH_2_Cl_2_/pyridine (1:1, 22.6 mL) and cooled to 0 °C to form a clear solution. Benzoyl chloride (0.34 mL, 2.92 mmol, 1.29 equiv) was added dropwise under stirring, and the reaction mixture was brought back to room temperature after 10 min. TLC analysis indicated full conversion after 3 h, after which the solvents were removed *in vacuo* by coevaporation with chloroform (3×). The concentrate was diluted with CH_2_Cl_2_ (70 mL) and successively washed with 0.25 M HCl (70 mL), followed by a saturated solution of NaHCO_3_ (70 mL), water (70 mL), and brine (70 mL). The organic layer was dried (MgSO_4_), concentrated *in vacuo*, and subjected to flash column chromatography over silica gel (3% MeOH in CH_2_Cl_2_) to yield **10** (792 mg, 2.18 mmol, **96%**) as a cloudy white solid. ***R***_***f***_: 0.24 (3% MeOH in CH_2_Cl_2_, UV/ninhydrin stain). ^**1**^**H NMR (500 MHz, CDCl**_**3**_**)** δ 8.03 (dd, *J* = 8.4, 1.4 Hz, 2H), 7.55 (dd, *J* = 7.4, 1.4 Hz, 1H), 7.43 (d, *J* = 7.4 Hz, 2H), 6.26 (s, 1H), 5.05 (d, *J* = 9.0 Hz, 1H), 4.38–4.26 (m, 2H), 4.07 (ddd, *J* = 11.9, 9.0, 3.5 Hz, 1H), 3.44–3.29 (m, 2H), 2.56–2.44 (m, 2H), 2.09 (ddd, *J* = 14.2, 11.9, 3.5 Hz, 1H), 1.85–1.79 (m, 1H), 1.55 (ddd, *J* = 13.6, 9.0, 3.5 Hz, 1H), 1.38 (s, 9H). ^**13**^**C{**^**1**^**H} NMR (126 MHz, CDCl**_**3**_**)** δ 180.4 (CO), 166.5 (CO), 156.0 (CO), 133.3 (ArH), 129.9 (ArC), 129.9 (ArH), 128.5 (ArH), 79.7 (C), 67.6 (CH_2_), 48.3 (CH), 40.5 (CH_2_), 38.2 (CH), 33.6 (CH_2_), 28.4 (CH_2_), 28.4 (CH_3_). **HRMS (ESI)**: *m*/*z* calcd for C_19_H_26_N_2_O_5_Na ([M + Na]^+^) 385.1743, found 385.1744. **Mp**: 158.8 °C, **[α]**_**D**_^**20**^ −26 (*c* = 2.0, CHCl_3_).

#### (*S*)-2-Formamido-3-((*S*)-2-oxopyrrolidin-3-yl)propyl Benzoate (**11**)

Benzoate **10** (1.06 g, 2.91 mmol, 1.0 equiv) was dissolved in dry CH_2_Cl_2_/TFA 1:1 (20 mL) to yield a clear, slightly yellow solution. The reaction mixture was stirred for 3 h at room temperature, after which the solvents were removed *in vacuo* by coevaporation with chloroform (3×). The yellow to orange oily residue was redissolved in dry CH_2_Cl_2_ (30 mL), and freshly prepared acetic formic anhydride (0.93 mL, 11.6 mmol, 4.0 equiv) was added. The reaction mixture was then cooled down to 0 °C, followed by dropwise addition of DIPEA (1.30 mL, 7.28 mmol, 2.5 equiv). Subsequently, the reaction mixture was brought back to room temperature and stirred overnight. Upon full conversion (as indicated by TLC) the reaction mixture was successively washed with 0.25 M HCl (30 mL), followed by a saturated solution of NaHCO_3_ (30 mL), water (30 mL), and brine (30 mL). The organic phase was then dried (Na_2_SO_4_), concentrated *in vacuo*, and subjected to column chromatography over silica gel (3–5% MeOH in CH_2_Cl_2_) to afford **11** (792 mg, 2.73 mmol, **92%**) as an off-white to faint yellow viscous oil. ***R***_***f***_: 0.35 (4% MeOH in CH_2_Cl_2_, UV). ^**1**^**H NMR (500 MHz, CDCl**_**3**_**)** δ 8.26 (s, 1H), 8.03 (dd, *J* = 8.4, 1.4 Hz, 2H), 7.56 (tt, *J* = 7.5, 1.4 Hz, 1H), 7.44 (t, *J* = 7.5 Hz, 2H), 6.99 (d, *J* = 8.0 Hz, 1H), 6.11 (s, 1H), 4.47 (ddd, *J* = 11.8, 8.0, 4.9 Hz, 1H), 4.38 (d, *J* = 4.9 Hz, 2H), 3.40–3.30 (m, 2H), 2.54–2.38 (m, 2H), 2.13 (ddd, *J* = 14.2, 11.8, 4.9 Hz, 1H), 1.85–1.77 (m, 1H), 1.64 (ddd, *J* = 14.2, 8.0, 3.5 Hz, 1H). ^**13**^**C{**^**1**^**H} NMR (126 MHz, CDCl**_**3**_**)** δ 180.4 (CO), 166.5 (CO), 161.8 (COH), 133.4 (ArH), 129.8 (ArH), 129.8 (ArC), 128.6 (ArH), 66.7 (CH_2_), 46.3 (CH), 40.6 (CH_2_), 38.3 (CH), 32.3 (CH_2_), 28.8 (CH_2_). **HRMS (ESI)**: *m*/*z* calcd for C_15_H_18_N_2_O_4_Na ([M + Na]^+^) 313.1159, found 313.1159. **[α]**_**D**_^**20**^ −25 (*c* = 2.0, CHCl_3_).

#### (*S*)-2-Isocyano-3-((*S*)-2-oxopyrrolidin-3-yl)propyl Benzoate (**4a**)

##### Procedure A

Formamide **11** (694 mg, 2.39 mmol, 1.0 equiv) was dissolved in dry CH_2_Cl_2_ (5.2 mL) and Et_3_N (3.3 mL, 23.9 mmol, 10.0 equiv). The solution was cooled to −78 °C before triphosgene (468 mg, 1.58 mmol, 0.66 equiv) was quickly added. The reaction mixture was stirred for 2 h, quenched with water (20 mL) and a saturated solution of NaHCO_3_ (20 mL), and then allowed to warm slowly to room temperature. The layers were separated, and the aqueous phase was re-extracted with CH_2_Cl_2_ (4 × 20 mL). The combined organic extracts were dried (MgSO_4_), concentrated in vacuo, and subjected to column chromatography over silica gel (3% MeOH in CH_2_Cl_2_) to afford isocyanide **4a** (546 mg, 2.01 mmol, **84%**) as a colorless crystalline solid.

##### Procedure B

Formamide **11** (880 mg, 3.03 mmol, 1.0 equiv) was dissolved in dry THF (30 mL) and Et_3_N (3.4 mL, 24.25 mmol, 8.0 equiv), after which the solution was cooled to 0 °C and trifluoroacetic anhydride (0.813 mL, 5.76 mmol, 1.9 equiv) was added. The reaction mixture was stirred for 30 min at this temperature, and the conversion carefully monitored by TLC analysis. Upon completion, the reaction mixture was diluted with NaHCO_3_ (35 mL) and water (35 mL) and then extracted with EtOAc (1 × 70 mL) and CH_2_Cl_2_ (5 × 70 mL). The combined organic extracts are dried (Na_2_SO_4_), filtered, concentrated *in vacuo*, and subjected to flash column chromatography (2.5%-3 MeOH in CH_2_Cl_2_) to afford isocyanide **4a** (686 mg, 2.52 mmol, **83%**) as a colorless crystalline solid which could be recrystallized from boiling acetone, which was then slowly allowed to cool to room temperature to give crystals suitable for X-ray diffraction. ***R***_***f***_: 0.42 (4% MeOH in CH_2_Cl_2_, KMnO_4_ stain). ^**1**^**H NMR (600 MHz, CDCl**_**3**_**)** δ 8.07 (dd, *J* = 8.4, 1.4 Hz, 2H), 7.60 (tt, *J* = 7.6, 1.4 Hz, 1H), 7.46 (d, *J* = 7.6 Hz, 2H), 6.38 (s, 1H), 4.49 (dd, *J* = 11.4, 4.3 Hz, 1H), 4.42 (dd, *J* = 11.4, 6.7 Hz, 1H), 4.14 (apparent ddt, *J* = 10.1, 6.7, 4.9 Hz, 1H), 3.39 (m, 2H), 2.68 (apparent ddt, *J* = 10.4, 8.6, 4.9 Hz, 1H), 2.43 (ddd, *J* = 12.4, 8.6, 4.8 Hz, 1H), 2.36 (ddd, *J* = 14.5, 10.1, 4.9 Hz, 1H), 1.82 (ddd, *J* = 12.4, 10.4, 9.0 Hz, 1H), 1.70 (ddd, *J* = 14.5, 10.1, 4.9 Hz, 1H).^**13**^**C{**^**1**^**H} NMR (126 MHz, CDCl**_**3**_**)** δ 178.7 (CO), 166.1 (CO), 158.8 (C), 133.7 (ArH), 129.9 (ArH), 129.2 (ArC), 128.7 (ArH), 65.2 (CH_2_), 52.0 (CH), 40.3 (CH_2_), 37.5 (CH), 32.9 (CH_2_), 27.7 (CH_2_). **HRMS (ESI)**: *m*/*z* calcd for C_15_H_16_N_2_O_3_Na ([M + Na]^+^) 295.1053, found 295.1056. **mp**: 151.2 °C. **[α]**_**D**_^**20**^ +6 (*c* = 2.0, CHCl_3_).

#### (*S*)-2-((1R,2S,5S)-3-((S)-3,3-dimethyl-2-(2,2,2-trifluoroacet-amido)butanoyl)-6,6-dimethyl-3-azabicyclo[3.1.0]-hexane-2-carboxamido)-3-((S)-2-oxopyrrolidin-3-yl)-propylbenzoate (**12**)

The imine bisulfite adduct **7** (1.876 g, 8.8 mmol, 2.2 equiv) was dissolved in 0.5 M NaOH (aq.) (20 mL) and stirred at room temperature for 1 h. The free imine **3** was extracted with CH_2_Cl_2_ (4 × 20 mL), dried over Na_2_SO_4_, and filtered, after which the carboxylic acid **2** (2.00 g, 8.8 mmol, 2.2 equiv) was added to the combined organic extracts and concentrated under reduced pressure. A solution of the isocyanide **4a** (1.089 g, 4 mmol, 1.0 equiv) in CH_2_Cl_2_ (8 mL) was added thereto, and the reaction mixture was stirred at room temperature for 48 h. Upon consumption of the isocyanide (as judged by TLC analysis), the reaction mixture was diluted with additional CH_2_Cl_2_ (200 mL) and successively washed with a solution 0.25 M HCl (2 × 200 mL), followed by a saturated solution of NaHCO_3_ (250 mL), water (250 mL) and brine (250 mL). The organic phase was then dried (Na_2_SO_4_), concentrated *in vacuo*, and subjected to column chromatography over silica gel (2–3% MeOH in CH_2_Cl_2_) to afford **12** (1.65 g, 2.72 mmol, **68%**, dr > 25:1) as a transparent to off-white amorphous solid. ***R***_***f***_: 0.40 (5% MeOH in CH_2_Cl_2_, KMnO_4_ stain). ^**1**^**H NMR (600 MHz, DMSO*****-d***_***6***_**)** δ 9.38 (d, *J* = 8.5 Hz, 1H), 8.20 (d, *J* = 9.0 Hz, 1H), 7.99 (dd, *J* = 8.4, 1.3 Hz, 2H), 7.67 (tt, *J* = 7.7, 1.3 Hz, 1H), 7.53 (d, *J* = 7.7 Hz, 2H), 4.42 (d, *J* = 8.5 Hz, 1H), 4.31–4.24 (m, 3H), 4.18 (s, 1H), 3.89 (dd, *J* = 10.2, 5.5 Hz, 1H), 3.67 (d, *J* = 10.2 Hz, 1H), 3.14 (t, *J* = 9.2 Hz, 1H), 3.01 (apparent td, *J* = 9.2, 7.1 Hz, 1H), 2.45 (apparent tdd, *J* = 11.7, 8.4, 3.4 Hz, 1H), 2.22–2.14 (m, 1H), 2.23–2.14 (m, 1H), 1.91 (ddd, *J* = 13.4, 11.7, 3.3 Hz, 1H), 1.62 (ddd, *J* = 12.2, 9.2, 3.1 Hz, 1H), 1.43 (dd, *J* = 7.8, 5.5 Hz, 1H), 1.35 (ddd, *J* = 14.7, 11.7, 3.0 Hz, 1H), 1.09 (d, *J* = 7.8 Hz, 1H), 0.99 (s, 9H), 0.87 (s, 3H), 0.82 (s, 3H). ^**13**^**C{**^**1**^**H} NMR (126 MHz, DMSO*****-d***_***6***_**)** δ 178.7 (CO), 170.9 (CO), 167.2 (CO), 165.5 (CO), 156.9 (q, *J* = 37.0 Hz, CO), 133.4 (ArH), 129.6 (ArC), 129.3 (ArH), 128.7 (ArH), 117.0 (q, *J* = 288 Hz, CF_3_), 66.9 (CH_2_), 60.4 (CH), 58.2 (CH), 47.7 (CH_2_), 45.4 (CH), 39.3 (CH_2_), 37.1 (CH), 34.6 (C), 32.3 (CH_2_), 30.8 (CH), 27.5 (CH_2_), 27.2 (CH), 26.3 (3 × CH_3_), 25.8 (CH_3_), 18.5 (C), 12.3 (CH_3_). ^**19**^**F NMR (470 MHz, DMSO*****-d***_***6***_**)** δ −72.9. **HRMS (ESI)**: *m*/*z* calcd for C_30_H_40_N_4_O_6_F_3_ ([M + H]^+^) 609.2894, found 609.2895. **[α]**_**D**_^**20**^ −62.0 (*c* = 2.0, CHCl_3_).

#### (1*R*,2*S*,5*S*)-3-((*S*)-3,3-Dimethyl-2-(2,2,2-trifluoroacetamido)butanoyl)-*N*-((*S*)-1-hydroxy-3-((*S*)-2-oxopyrrolidin-3-yl)propan-2-yl)-6,6-dimethyl-3-azabicyclo[3.1.0]hexane-2-carboxamide (**13**)

U3CR adduct **12** (1.556 g, 2.556 mmol, 1.0 equiv) was dissolved in MeOH (51 mL), and K_2_CO_3_ (1.237 mg, 8.948 mmol, 3.5 equiv) was added. The reaction mixture was stirred at room temperature for 1.5 h until full conversion of the starting material was observed by TLC analysis. Upon completion, the reaction mixture was first quenched with a saturated solution of NH_4_Cl (30 mL) and further diluted with water (30 mL). The aqueous layer was then extracted with EtOAc (5 × 50 mL), after which the combined organic layers were collected, dried (Na_2_SO_4_), concentrated *in vacuo*, and subjected to column chromatography (5–7% MeOH in CH_2_Cl_2_) to afford **13** (1.098 g, 2.18 mmol, **85%**, dr > 25:1) as a colorless amorphous solid. ***R***_***f***_: 0.22 (5% MeOH in CH_2_Cl_2_, KMnO_4_ stain). ^**1**^**H NMR (600 MHz, DMSO*****-d***_***6***_**)** δ 9.39 (d, *J* = 8.5 Hz, 1H), 7.83 (d, *J* = 9.3 Hz, 1H), 7.46 (s, 1H), 4.67 (apparent t, *J* = 5.7 Hz, 1H), 4.43 (d, *J* = 8.5 Hz, 1H), 4.20 (s, 1H), 3.90 (dd, *J* = 10.5, 5.5 Hz, 1H), 3.87–3.78 (m, 1H), 3.69 (d, *J* = 10.5 Hz, 1H), 3.40–3.34 (m, 1H), 3.33–3.25 (m, 1H), 3.12 (d, *J* = 9.4 Hz, 1H), 3.00 (td, *J* = 9.4, 7.0 Hz, 1H), 2.40 (apparent tdd, *J* = 12.0, 8.3, 3.3 Hz, 1H), 2.23–2.15 (m, 1H), 1.73 (ddd, *J* = 13.7, 12.0, 3.3 Hz, 1H), 1.54 (ddd, J = 12.0, 9.4, 7.0 Hz, 1H), 1.51 (dd, *J* = 7.7, 5.5 Hz, 1H), 1.32 (d, *J* = 7.7 Hz, 1H), 1.30 (ddd, *J* = 12.0, 10.7, 2.9 Hz, 1H), 1.02 (s, 3H), 0.99 (s, 9H), 0.85 (s, 3H). ^**13**^**C{**^**1**^**H} NMR (126 MHz, DMSO*****-d***_***6***_**)** δ 179.1 (CO), 170.5 (CO), 167.1 (CO), 156.8 (q, *J* = 37.3 Hz, CO), 114.9 (q, *J* = 287.5 Hz, CF_3_), 64.2 (CH_2_), 60.4 (CH), 58.2 (CH), 48.4 (CH), 47.7 (CH_2_), 39.3 (CH_2_), 37.1 (CH), 34.7 (C), 33.0 (CH_2_), 30.8 (CH), 27.8 (CH_2_), 27.2 (CH), 26.3 (3x CH_3_), 25.9 (CH_3_), 18.5 (C), 12.4 (CH_3_). ^**19**^**F NMR (470 MHz, DMSO*****-d***_***6***_**)** δ −73.0. **HRMS (ESI)**: *m*/*z* calcd for C_23_H_36_N_4_O_5_F_3_ ([M + H]^+^) 505.2632, found 505.2646. **[α]**_**D**_^**20**^ −76 (*c* = 2.0, CHCl_3_).

#### (1*R*,5*S*)-*N*-((S)-1-Cyano-2-((*S*)-2-oxopyrrolidin-3-yl)ethyl)-3-((*S*)-3,3-dimethyl-2-(2,2,2-trifluoroacetamido)butanoyl)-6,6-dimethylazabicyclo[3.1.0]hexane-2-carboxamide (Nirmatrelvir, **1**)

To a solution of alcohol **13** (897 mg, 1.78 mmol, 1.0 equiv) in CH_3_CN/H_2_O 9:1 (18.8 mL) were successively added TEMPO (13.9 mg, 0.09 mmol, 0.05 equiv), PhI(OAc)_2_ (1.260 g, 3.91 mmol, 2.2 equiv), and NH_4_OAc (548 mg, 7.11 mmol, 4.0 equiv) The yellow solution was then stirred at room temperature for 2 h, and the conversion was carefully monitored by TLC analysis (*p*-anisaldehyde stain). Upon completion, the reaction mixture was first quenched with a 35 wt % Na_2_S_2_O_3_ solution (25 mL) and further diluted with water (25 mL). The aqueous layer was then extracted with EtOAc (4 × 30 mL), after which the combined organic layers were washed with water (100 mL) and brine (100 mL), dried (Na_2_SO_4_), filtered, concentrated *in vacuo*, and subjected to column chromatography over silica gel (2–4% MeOH in CH_2_Cl_2_) to afford **1** (nirmatrelvir) (740 mg, 1.48 mmol, **83%**, dr > 25:1) as a white foam. ***R***_***f***_: 0.42 (5% MeOH in CH_2_Cl_2_). ^**1**^**H NMR (600 MHz, DMSO*****-d***_***6***_**)** δ 9.41 (d, *J* = 8.5 Hz, 1H), 9.02 (d, *J* = 8.6 Hz, 1H), 7.67 (s, 1H), 4.97 (ddd, *J* = 11.0, 8.6, 5.2 Hz, 1H), 4.42 (d, *J* = 8.5 Hz, 1H), 4.16 (s, 1H), 3.92 (dd, *J* = 10.5, 5.5 Hz, 1H), 3.70 (d, *J* = 10.5 Hz, 1H), 3.15 (t, *J* = 9.2 Hz, 1H), 3.05 (dd, *J* = 9.2, 7.1 Hz, 1H), 2.41 (apparent tdd, *J* = 10.5, 8.4, 4.5 Hz, 1H), 2.15 (ddd, *J* = 13.5, 10.9, 4.5 Hz, 1H), 2.12–2.05 (m, 1H), 1.78–1.66 (m, 2H), 1.57 (dd, *J* = 7.7, 5.5 Hz, 1H), 1.32 (d, *J* = 7.7 Hz, 1H), 1.03 (s, 3H), 0.99 (s, 9H), 0.86 (s, 3H). ^**13**^**C{**^**1**^**H} NMR (126 MHz, DMSO*****-d***_***6***_**)** δ 177.5 (CO), 170.7 (CO), 167.4 (CO), 156.9 (q, *J* = 37.5 Hz, CO), 119.6 (CN), 115.8 (q, *J* = 285 Hz, CF_3_), 60.1 (CH), 58.2 (CH), 47.6 (CH_2_), 39.3 (CH_2_), 37.8 (CH), 36.7 (CH), 34.6 (CH_2_), 34.1 (C), 30.3 (CH), 27.3 (CH), 26.9 (CH_2_), 26.2 (3x CH_3_), 25.7 (CH_3_), 18.8 (C), 12.3 (CH_3_). ^**19**^**F NMR (470 MHz, DMSO*****-d***_***6***_**)** δ −72.9. **HRMS (ESI)**: *m*/*z* calcd for C_23_H_33_N_5_O_4_F_3_ ([M + H]^+^) 500.2479, found 500.2479. **[α]**_**D**_^**20**^ −67 (*c* = 2.0, CHCl_3_).

## Data Availability

The data underlying this study are available in the published article and its Supporting Information.

## Supporting Information Available

Comparison to reported route, supplementary experimental procedures and characterization data, crystal data for **4a**, NMR spectra for all compounds, and SFC traces for **12**, **13**, and **1** (PDF)
FAIR data, including the primary NMR FID files, for compounds **1**, **13**, **12**, **4a**, **11**, **10** and **9** (ZIP)

## Notes

An earlier version of this work was posted as a preprint: Preschel, H. D.; Otte, R. T.; Zhuo, Y.; Ruscoe, R. E.; Burke, A. J.; Cowan, R.; Horst, B.; Hennig, S.; Janssen, E.; Green, A.; Turner, N. J.; Ruijter, E. *ChemRxiv***2022**. DOI: 10.26434/chemrxiv-2022-8r8h7.
