# Supplementary material for: Multicomponent Synthesis of the SARS-CoV-2 Main Protease Inhibitor Nirmatrelvir
Source: J Org Chem. 2023 Aug 22;88(17):12565–71. doi: 10.1021/acs.joc.3c01274 (PMC10476182; doi:10.1021/acs.joc.3c01274)
Supplement: Supplementary file 1 — jo3c01274_si_001.pdf [file jo3c01274_si_001.pdf]

Multicomponent Synthesis of the SARS-CoV-2 Main Protease Inhibitor Nirmatrelvir

H. Daniel Preschel,[a] Ruben T. Otte,[a] Ying Zhuo,[b] Rebecca E. Ruscoe,[b] Ashleigh J. Burke,[b] Rachel Kellerhals,[a] Brendan Horst,[a] Sven Hennig,[a] Elwin Janssen,[a] Anthony P. Green,[b] Nicholas J. Turner,[b] Eelco Ruijter[a] *

[a] Department of Chemistry & Pharmaceutical Sciences, Amsterdam Institute of Molecular & Life Sciences (AIMMS), Vrije Universiteit Amsterdam, De Boelelaan 1108, 1081 HZ Amsterdam (The Netherlands)

[b] Department of Chemistry, The University of Manchester, Manchester Institute of Biotechnology, 131 Princess Street, Manchester M1 7DN (United Kingdom)

* Email: e.ruijter@vu.nl

Table of Contents

Contents

General Information S2

Comparison of synthetic routes S3

Biotransformation of **6** to **3** S4

Construction of pET29b(+)_MAON S4

Protein production and purification S4

General procedure for analytical scale biotransformation S4

DNA sequence of MAON-401 ..... S5

Alignment of MAON variants S6

Derivatization for Enantioselectivity Determination S7

GC-MS data S7

Chiral GC analysis S8

Supplementary Synthetic Procedures and Compound Characterization S9

X-Ray crystal structure of **4a** S13

NMR Spectra S16

HRMS S34

SFC-MS S35

References S36

General Information

Compounds **2** and **5** were purchased from Angene Chemical. Other commercially available reagents were purchased from Merck (Sigma-Aldrich), Fischer Scientific, Strem Chemicals, TCI Europe or Fluorochem and were used as received, unless mentioned otherwise. Solvents were purchased from VWR Chemicals or Sigma-Aldrich and used without purification, unless stated otherwise. Anhydrous, air-free solvents (CH_2Cl_2, toluene, THF) were obtained from a PureSolv MD 5 solvent purification system. Nuclear magnetic resonance (NMR) spectra were recorded on a Bruker Avance 600, Bruker Avance 500 or Bruker Avance 300 using the residual $CHCl_3$ signal (^{1}H: δ 7.26 ppm) or the $CDCl_3$ signal (^{13}C{^{1}H}: δ 77.16 ppm) as internal reference standard. Chemical shifts (δ) are given in ppm and coupling constants (*J*) are quoted in hertz (Hz). Resonances are described as s (singlet), d (doublet), t (triplet), q (quartet), br (broad singlet) and m (multiplet) or combinations thereof. Structural assignments were made with additional information from gCOSY, gHSQC, and gHMBC experiments. Electrospray Ionization (ESI) high-resolution mass spectrometry was carried out using a Bruker micrOTOF-Q instrument in positive ion mode (capillary potential of 4500 V). Flash chromatography was performed on Silicycle Silica-P Flash Silica Gel (particle size 40-63 µm, pore diameter 60 Å) using the indicated eluent. Thin Layer Chromatography (TLC) was performed using TLC plates from Merck (SiO_2, Kieselgel 60 F254 neutral, on aluminum with fluorescence indicator) and compounds were visualized by UV detection (254 nm), potassium permanganate, cerium (IV) sulfate, ninhydrin, and/or *p*-anisaldehyde stain. SFC-MS analysis was conducted using a Shimadzu Nexera SFC-MS equipped with a Nexera X2 SIL-30AC autosampler, Nexera UC LC-30AD SF CO_2 pump, Nexera X2 LC-30AD liquid chromatograph, Nexera UC SFC-30A back pressure regulator, prominence SPD-M20A diode array detector, prominence CTO-20AC column oven and CBM-20A system controller, coupled to a. Shimadzu LCMS-2020 mass spectrometer. The data were acquired in full-scan APCI mode (MS) from m/z 100 to 800 in positive ionization mode. Data was processed using Shimadzu Labsolutions 5.82. Enantiomeric excess was determined by SFC-MS analysis using: Method A) Lux® 3 µm Cellulose-3 column (cellulose tris(4-methylbenzoate), 150 x 4.6 mm) eluting with an isocratic mixture of supercritical CO_2 (99%) and methanol (1%) at 1 mL per minute, run length of 10 minutes, detection at 246 nm; Method B) Lux® 3 µm Cellulose-1 column (cellulose tris(3,5-dimethylphenylcarbamate). eluting with an isocratic mixture of supercritical CO_2 (70%) and methanol (30%) at 2 mL per minute, run length of 5.5 minutes, detection at 224 nm. The sample injection volume was 5 µL. Specific rotations were measured on a Krüss P3000 polarimeter, using a 0.5 dm cell and solvent as indicated. GC-FID analysis was performed on an Agilent 6850 GC with a Gerstel Multipurpose sampler MPS2L using β-dex 325 (Supelco) with dimensions 30 m x 0.25 mm x 0.25µm. X-ray crystallographic data were obtained from a Bruker D8 Quest instrument, equipped with PHOTON II detector. X-rays were generated from an INCOATEC IµS 3.0 Cu-Kα sealed X-ray tube source at 1.54178 Å. Measurements were carried out at 100(2) K, using an Oxford Cryosystems CRYOSTREAM 800.

Comparison of synthetic routes

To assess the efficiency of our route to the original route reported by Pfizer,[1] we compared several characteristics of both routes. In order to make a fair comparison, it was assumed the bicyclic proline derivative **15**, a key intermediate in the Pfizer synthesis, was obtained from **7** as reported previously.[2] Thus, the two routes were compared starting from the common intermediates **5** and **7**.

Scheme S1. Comparison of synthetic route to reported route to nirmatrelvir[1]

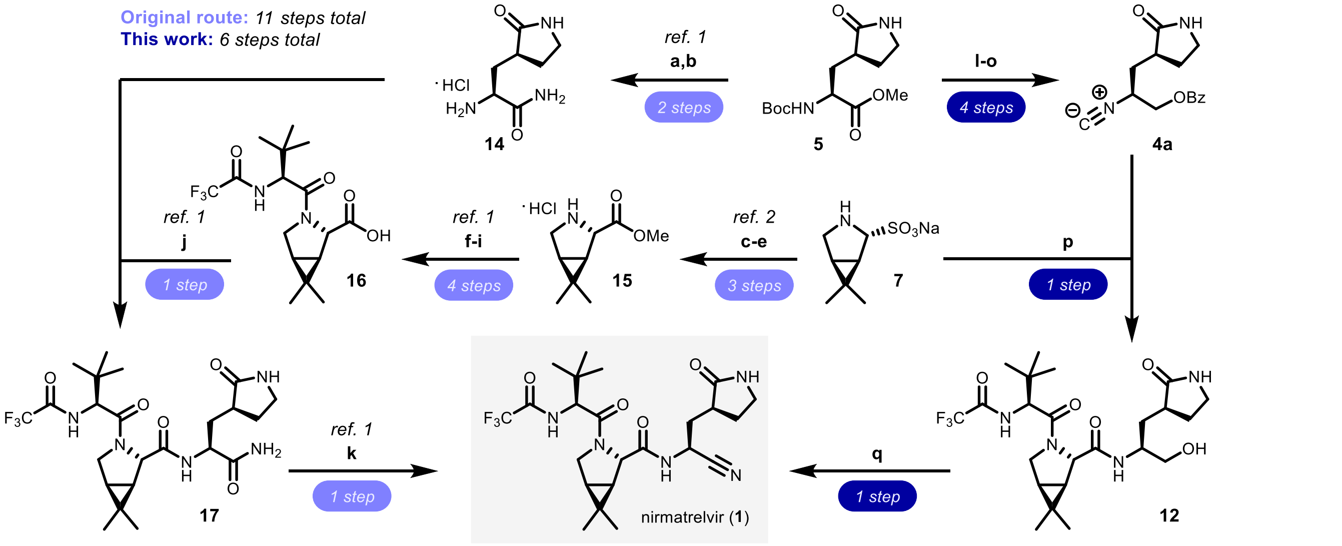

Reagents and conditions: a) NH_3, MeOH, 98%; **b**) HCl, *i*PrOH, quant.; **c**) NaCN, CPME; **d**) 5 N HCl, MeOH, 50 °C; then aq. NaOH, *t*BuOMe; **e**) HCl, *i*PrOH/*t*BuOMe, 56% over three steps; **f**) Boc-Tle-OH, HATU, *i*Pr_2NEt, DMF/MeCN, 0 °C, 96%; **g)** LiOH, THF/H_2O/MeOH, 0 °C, quant.; **h**) 4 M HCl, dioxane/CH_2Cl_2, 0 °C, 99%; **i)** F_3CCO_2Et, Et_3N, MeOH, 0 °C, 71%; **j)** EDCI, DIPEA, HOPO, MEK, 98%; **k)** Burgess reagent, CH_2Cl_2, 75%; **l**) $LiBH_4$, THF, 89%; **m**) BzCl, py, CH_2Cl_2, 96%; **n**) TFA, CH_2Cl_2, then AcOCHO, *i*Pr_2NEt, 92%; **o**) $(Cl_3CO)_2CO$, Et_3N, CH_2Cl_2, −78 °C, 84% [*alternatively*: TFAA, Et_3N, CH_2Cl_2, 0 °C, 83%]; **p**) aq. NaOH, CH_2Cl_2, then $F_3CC(O)$-Tle-OH (**2**), then **4a**, MeOH; then K_2CO_3; **q**) cat. TEMPO, $PhI(OAc)_2$, NH_4OAc, MeCN/H_2O, 70% over two steps.

Table S1: Comparison of step economy and overall yield

	**Original route[1]**	**This work**
Total # steps	**11**	**6**
Longest linear sequence (LLS)	**9 steps (7** to **1)**	**6 steps (5** to **1)**
Overall yield (over LLS)	**27.7%**	**46.2%**
Chromatographic purifications	**2**	**2**

Biotransformation of 6 to 3

Construction of pET29b(+)_MAON

The gene (synthesized by Integrated DNA Technologies) for monoamine oxidase (MAON) from literature reports[2,3] was cloned into pET29b(+) using NdeI and XhoI restriction sites to yield pET29b(+)_MAON.

Protein production and purification

For expression of MAON enzymes, chemically competent *E. coli* BL21 (DE3) were transformed with the relevant pET29b(+)_MAON constructs. Single colonies of freshly transformed cells were cultured for 18 h in 5 mL LB medium containing 50 µg mL^{-1} kanamycin. Starter cultures (500 µL) were used to inoculate 50 mL 2×YT medium supplemented with 50 µg mL^{-1} kanamycin. Cultures were grown at 37 °C, 200 rpm. to an optical density at 600 nm (OD600) of around 0.5. Protein expression was induced with the addition of IPTG to a final concentration of 0.1 mM. Induced cultures were incubated for 20 h at 30 °C and the cells were subsequently collected by centrifugation (3,220 x g for 10 min). Pelleted cells were resuspended in lysis buffer (50 mM HEPES, 300 mM NaCl, pH 7.5 containing 20 mM imidazole) and lysed by sonication. Cell lysates were cleared by centrifugation (27,216 x g for 30 min) and supernatants were subjected to affinity chromatography using Ni-NTA Agarose (Qiagen). Purified protein was eluted using 50 mM HEPES, 300 mM NaCl, pH 7.5 containing 250 mM imidazole. Proteins were desalted using 10DG desalting columns (Bio-Rad) with PBS pH 7.4. SDS-PAGE was run to confirm purity/weight (Figure S1). Proteins were aliquoted, flash-frozen in liquid nitrogen and stored at −80 °C. Protein concentrations were determined by measuring the absorbance at 280 nm and assuming an extinction coefficient of 99350 M^{-1} cm^{-1}.

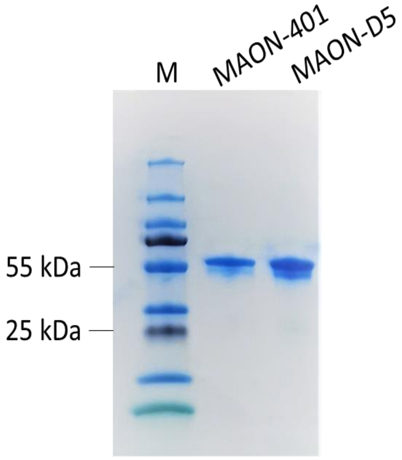

Figure S1. SDS-PAGE analysis of purified MAON variants

General procedure for analytical scale biotransformation

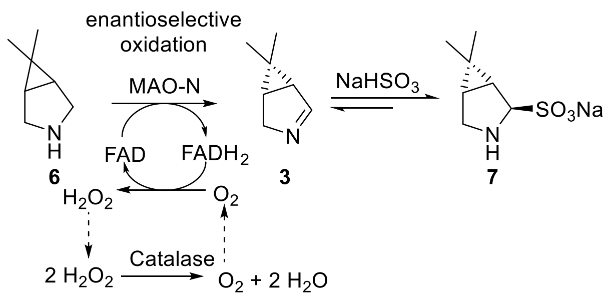

Scheme S2. Oxidative desymmetrization of substrate **6**.

To compare the activity of MAON variants, analytical scale biotransformations were performed using **6** (25 mM), MAON enzyme (1 µM) and 0.6% catalase in 200 µl PBS buffer (pH 7.4) (Scheme S1). For GCMS analysis, reactions were quenched with the addition of 0.25 volume of 10 N NaOH, then extracted with 1 volume of MTBE. Samples were vortexed and precipitated proteins were removed by centrifugation (14,000 x g for 5 minutes). The extraction solution was dried over anhydrous $MgSO_4$, and analyzed by GCMS to monitor the conversion (Figure S2).
Consistent with previous reports,[1] both enzymes showed activity on **6** in the amplex red-based assay (data not shown). All the substrate was completely converted to the product **3** in 6 h incubation at 30 °C (0.01% enzyme loading). The product **3** from MAON assay was determined to be the desired enantiomer with an *ee* ≥99% (see derivatization data). Of the two active amine oxidases, the MAON-401 was more stable upon storage at −80 °C and in fridge for two weeks.

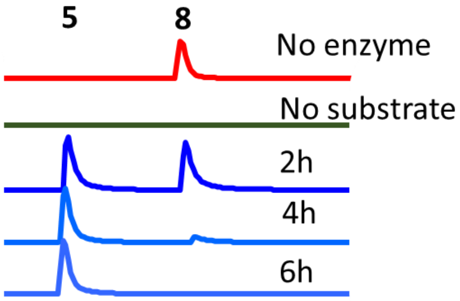

Figure S2. GCMS traces showing amine substrate **6** (25 mM) conversion to imine **3** at 2, 4 and 6 hour time points catalyzed by MAON-401 (1 µM) in PBS buffer pH 7.4.

DNA sequence of MAON-401

>MAON-401_DNA sequence
ATGACAAGCCGTGATGGCTATCAATGGACACCCGAAACGGGCTTAACGCAAGGAGTACCTAGTTTAGGGGTCATCTCACCGCCCACCAA
CATTGAAGATACGGATAAGGACGGGCCATGGGACGTTATTGTTATCGGGGGTGGGTACTGCGGCTTGACGGCGACACGCGACTTGACC
GTAGCTGGTTTCAAAACGTTGCTGCTTGAGGCTCGTGACCGCATTGGAGGTCGCTCATGGTCCTCCAACATTGACGGCTACCCGTATGAA
ATGGGAGGCACTTGGGTGCACTGGCATCAAAGCCACGTCTGGCGTGAGATTACTCGCTACAAGATGCACAATGCGCTTTCACCTTCCTTT
AATTTTTCCCGCGGGGTGAATCATTTTCAACTTCGTACAAACCCCACCACGTCGACCTATATGACCCACGAGGCTGAGGATGAGTTGCTT
CGTTCGGCTCTGCATAAGTTCACCAACGTAGATGGGACGAACGGACGTACCGTGTTGCCGTTCCCGCATGACATGTTCTATGTTCCAGAA
TTTCGCAAGTATGATGAGATGTCCTATTCGGAACGCATTGATCAGATCCGTGACGAGCTTAGTCTGAACGAGCGCTCAAGCCTGGAGGC
CTTTATTTTGCTGTGTAGCGGTGGGACACTGGAAAACTCTTCGTTCGGTGAGTTCTTGCATTGGTGGGCAATGTCAGGGTATACCTATCA
AGGTTGTATGGACTGCCTGATCAGCTACAAGTTCAAAGACGGGCAATCAGCCTTCGCTCGTCGCTTTTGGGAGGAAGCCGCTGGCACTG
GGCGCCTGGGATACGTCTTTGGTTGTCCGGTTCGTTCTGTTGTCAATGAGCGCGACGCAGTGCGTGTTACCGCCCGCGATGGACGCGAA
TTCGCCGCCAAGCGTCTTGTTTGTACTATTCCCCTGAACGTCTTGAGCAGTGTCCACTTTAGTCCTCCGTTATCACCTCAACGTATGGCAG
CCGCCAACATTGGACATGTTAACCAATGTGTGAAAGTTCACGCTGAAGTGAGTTGTCCTGACATGCGCAGCTGGTCGGGTATCAGCTAT
CCGTTCAACAAACTTGCCTACGCGATTGGAGACGGTACAACACCGGCGGGGAATACTCATATTGTGTGCCTTGGGGGAGCCCATAATCA
TATCCAACCGGAAGAGGACGTGGAAGCAACGAAAATGGCAGTGGAGAACATGTCGCCGGGAAACATGGATATCAAACGCTTAGTTTTT
CATAATTGGTGTAAGGACGAATTTGCCAAAGGTGCGTGGTTCTTTGCCCCGCCTCAGCTGTTAAGCAAAAGCCTTGATGAGTTACGTTGC
CGCCATGGAAATGTTTTGTTTGCCAATTCAGACTGGGCGCTGGGATGGCGCGGGTTTATTGACGGAGCCATTGAAGAAGGGACACGTG
CTGCGGTAACTGTGATCGAAGAGCTTCGTCCAGCCCCCGCAGTCCGCTCGCACCTTCTCGAGCACCACCACCACCACCACTGA

Alignment of MAON variants

```
MAON-WT         MTSRDGYQWTPETGLTQGVPSLGVISPPTNIEDTDKDGPWDVIVIGGGYCGLTATRDLTV 60
MAON-D5         MTSRDGYQWTPETGLTQGVPSLGVISPPTNIEDTDKDGPWDVIVIGGGYCGLTATRDLTV 60
MAON-401        MTSRDGYQWTPETGLTQGVPSLGVISPPTNIEDTDKDGPWDVIVIGGGYCGLTATRDLTV 60
                ************************************************************

MAON-WT         AGFKTLLLEARDRIGGRSWSSNIDGYPYEMGGTWVHWHQSHVWREITRYKMHNALSPSFN 120
MAON-D5         AGFKTLLLEARDRIGGRSWSSNIDGYPYEMGGTWVHWHQSHVWREITRYKMHNALSPSFN 120
MAON-401        AGFKTLLLEARDRIGGRSWSSNIDGYPYEMGGTWVHWHQSHVWREITRYKMHNALSPSFN 120
                ************************************************************

MAON-WT         FSRGVNHFQLRTNPTTSTYMTHEAEDELLRSALHKFTNVDGTNGRTVLPFPHDMFYVPEF 180
MAON-D5         FSRGVNHFQLRTNPTTSTYMTHEAEDELLRSALHKFTNVDGTNGRTVLPFPHDMFYVPEF 180
MAON-401        FSRGVNHFQLRTNPTTSTYMTHEAEDELLRSALHKFTNVDGTNGRTVLPFPHDMFYVPEF 180
                ************************************************************

MAON-WT         RKYDEMSYSERIDQIRDELSLNERSSLEAFILLCSGGTLENSSFGEFLHWWAMSGYTYQG 240
MAON-D5         RKYDEMSYSERIDQIRDELSLNERSSLEAFILLCSGGTLENSSFGEFLHWWAMSGYTYQG 240
MAON-401        RKYDEMSYSERIDQIRDELSLNERSSLEAFILLCSGGTLENSSFGEFLHWWAMSGYTYQG 240
                ************************************************************

MAON-WT         CMDCLISYKFKDGQSAFARRFWEEAAGTGRLGYVFGCPVRSVVNERDAVRVTARDGREFV 300
MAON-D5         CMDCLMSYKFKDGQSAFARRFWEEAAGTGRLGYVFGCPVRSVVNERDAARVTARDGREFV 300
MAON-401        CMDCLISYKFKDGQSAFARRFWEEAAGTGRLGYVFGCPVRSVVNERDAVRVTARDGREFA 300
                *****:******************************************.**********.

MAON-WT         AKRVVCTIPLNVLSTIQFSPALSTERISAMQAGHVNMCTKVHAEVDNKDMRSWTGIAYPF 360
MAON-D5         AKRVVCTIPLNVLSTIQFSPALSTERISAMQAGHVSMCTKVHAEVDNKDMRSWTGIAYPF 360
MAON-401        AKRLVCTIPLNVLSSVHFSPPLSPQRMAAANIGHVNQCVKVHAEVSCPDMRSWSGISYPF 360
                ***:**********:::***.**.:*::* : ***. *.******.  *****:**:***

MAON-WT         NKLCYAIGDGTTPAGNTHLVCFGTDANHIQPDEDVRETLKAVGQLAPGTFGVKRLVFHNW 420
MAON-D5         NKLCYAIGDGTTPAGNTHLVCFGNSANHIQPDEDVRETLKAVGQLAPGTFGVKRLVFHNW 420
MAON-401        NKLAYAIGDGTTPAGNTHIVCLGGAHNHIQPEEDVEATKMAVENMSPGNMDIKRLVFHNW 420
                ***.**************:**:*   *****:***. *  ** :::**.:.:********

MAON-WT         VKDEFAKGAWFFSRPGMVSECLQGLREKHGGVVFANSDWALGWRSFIDGAIEEGTRAARV 480
MAON-D5         VKDEFAKGAWFFSRPGMVSECLQGLREKHGGVVFANSDWALGWRSFIDGAIEEGTRAARV 480
MAON-401        CKDEFAKGAWFFAPPQLLSKSLDELRCRHGNVLFANSDWALGWRGFIDGAIEEGTRAAVT 480
                 ***********: * ::*:.*: ** :**.*:***********.************* .

MAON-WT         VLEELGTKREVKARL 495
MAON-D5         VLEELGTKREVKARL 495
MAON-401        VIEELRPAPAVRSHL 495
                *:*** .   *:::*
```

Derivatization for Enantioselectivity Determination

In order to determine the *ee* of compound **3** obtained from the biotransformation, it was compared to a racemic standard. Both the racemate and the enantioenriched material were reacted with phenylmagnesium bromide to give the stable Grignard adduct **S1**. Comparison of the peak surface are of the major diastereomer by chiral GC-FID allowed accurate determination of the *ee* of **3**.

racemic standard:

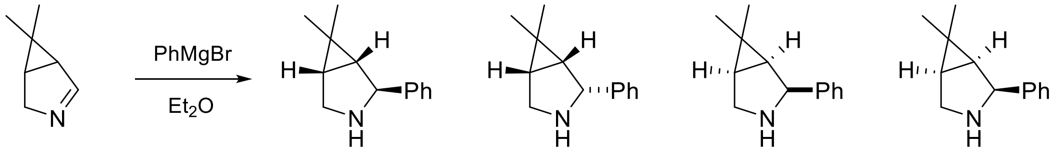

rac-**3** | *trans*-**S1** (major) | *cis*-**S1** (minor) | *trans*-**S1** (major) | *cis*-**S1** (minor)

enantioenriched from biotransformation:

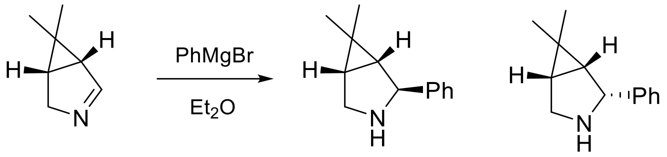

3 | *trans*-**S1** (major) | *cis*-**S1** (minor)

GC-MS data

Racemic standard *rac*-S1:

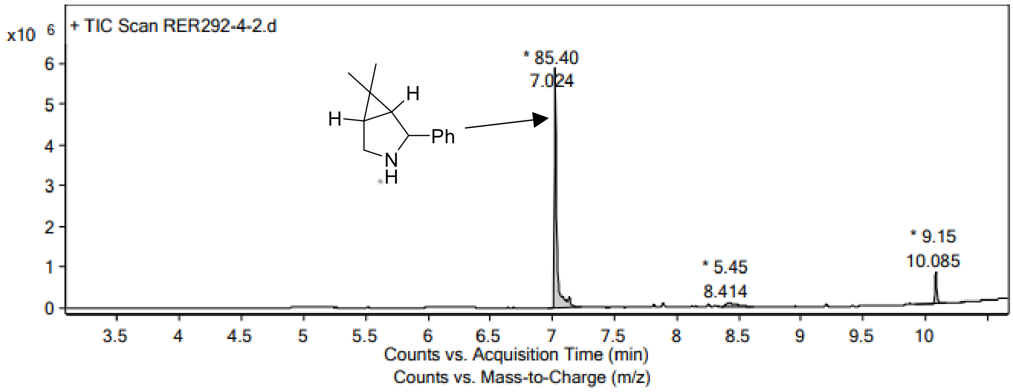

Sample from biotransformation S1:

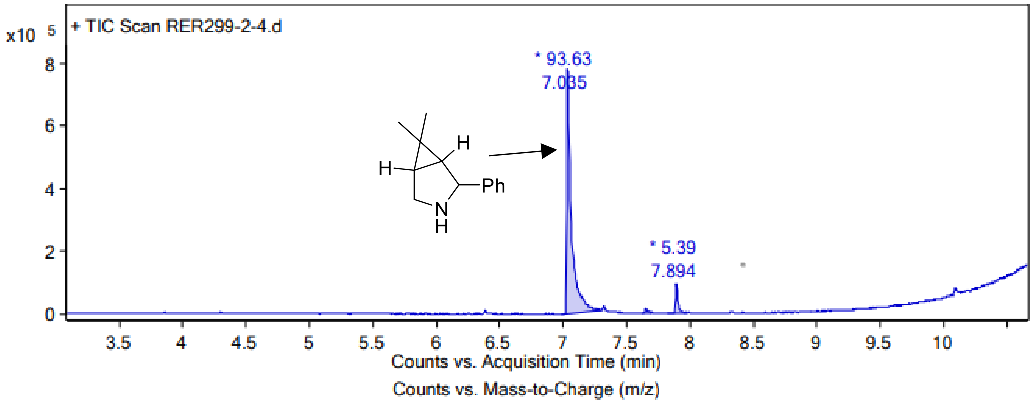

Chiral GC analysis

(a) Racemic Standard

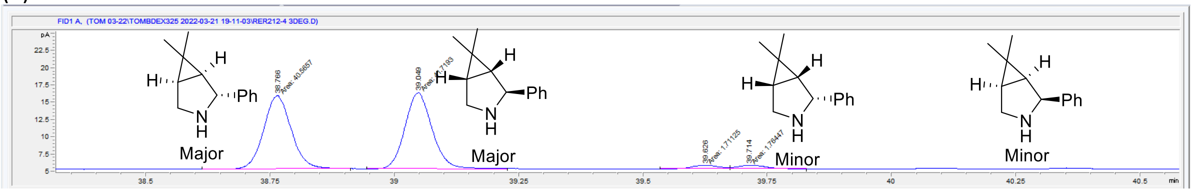

(b) Sample from Biotransformation

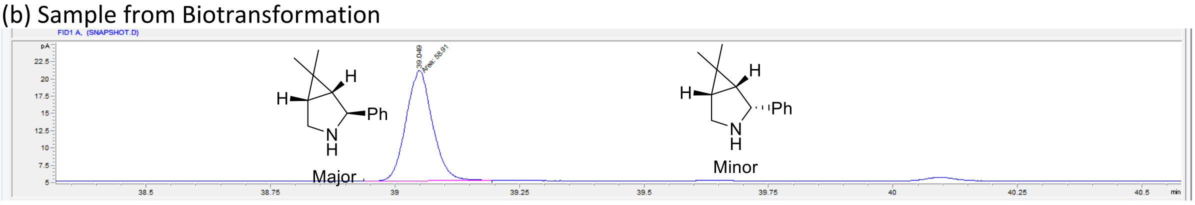

Zoomed in spectra of above:

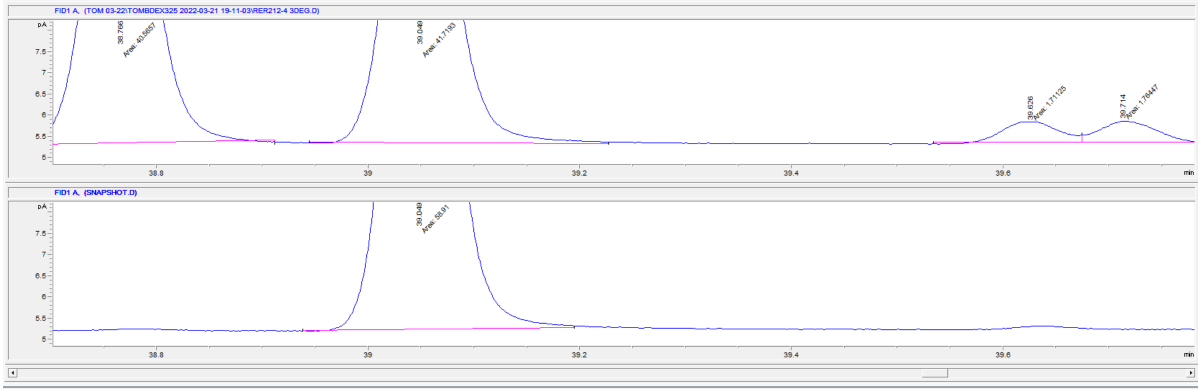

(a) Racemic Standard

Time (min)	Area
38.766	40.5657
39.049	41.7193
39.626	1.71125
39.714	1.76447

(b) Sample from Biotransformation

Time (min)	Area	
39.049	58.91	

Only one peak corresponding to major diastereomer could be integrated. Therefore, the enantiomeric excess of **S1** from the biotransformation is ≥99%, suggesting an enantioselective formation of imine by MAO-N 401. Imine **3** is formed with ≥99% *ee* by MAO-N 401.

Supplementary Synthetic Procedures and Compound Characterization

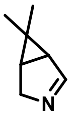

rac-**3**

***rac*-6,6-Dimethyl-3-azabicyclo[3.1.0]hex-2-ene (3):** 6,6-Dimethyl-3-azabicyclo[3.1.0]hexane (**6**) (0.33 mL, 2.7 mmol, 1.0 equiv.) was added to a solution of NaOH (460 mg, 11.5 mmol, 4.3 equiv.), $K_2S_2O_8$ (811 mg, 3.00 mmol, 1.1 equiv.) in water (4.5 mL) and MeCN (1.0 mL) at −5 °C under an atmosphere of nitrogen. After stirring for 1 hour at this temperature, a solution of $AgNO_3$ (23 mg, 0.14 mmol, 5 mol%) was added dropwise to the reaction mixture and the reaction mixture was left to stir at -5 °C for 1 hour. The reaction mixture was warmed to room temperature before being diluted with water (5 mL). The pH was corrected to 7 and extracted with Et_2O (3 x 5 mL). The combined organics were dried ($MgSO_4$) and concentrated *in vacuo* to give 6,6-dimethyl-3-azabicyclo[3.1.0]hex-2-ene (*rac*-**3**) as a pale yellow oil (34 mg, 0.31 mmol, **12%**). **^{1}H NMR (400 MHz, $CDCl_3$)** δ 7.37 (1H, app. t, *J* = 2.4 Hz, N=CH), 3.87 (1H, app. ddd, *J* = 18.1, 7.4, 2.0 Hz), 3.57 (1H, app. dq, *J* = 17.8, 2.6 Hz), 2.13 (1H, app. dd, *J* = 6.4, 2.9 Hz), 1.68 (1H, td, *J* = 6.4, 1.8 Hz), 1.11 (3H, s, CH_3), 0.76 (3H, s, CH_3). Data are consistent with the literature.[2]

GC-MS trace of **3**:

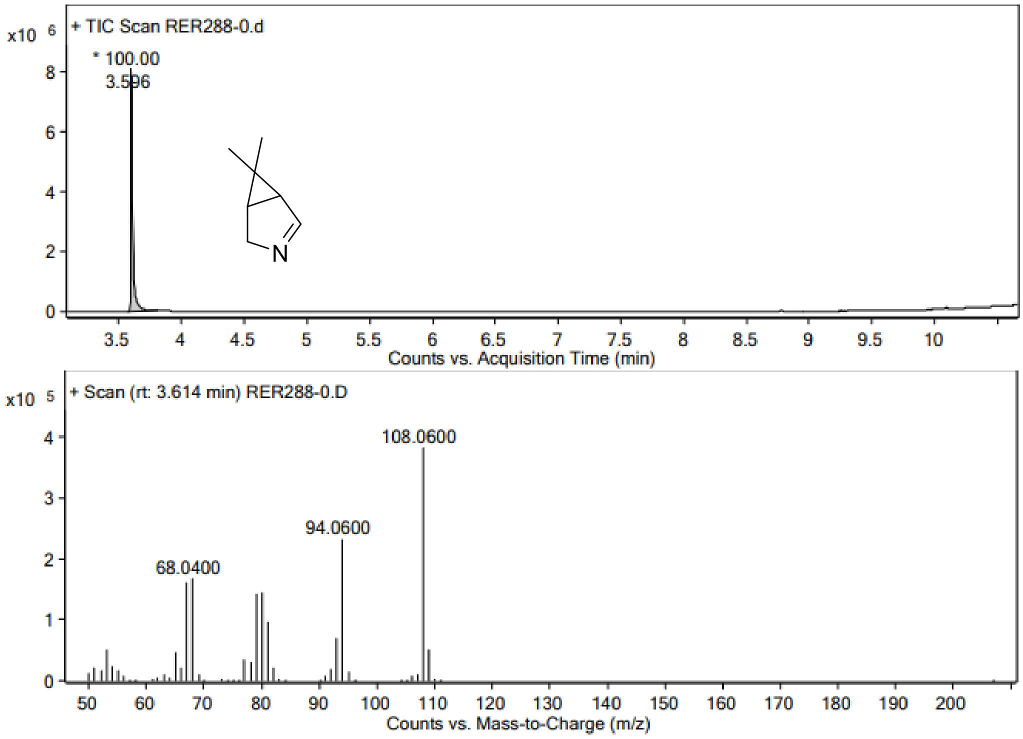

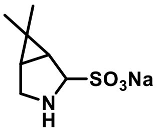

rac-**7**

Sodium *rac*-6,6-dimethyl-3-azabicyclo[3.1.0]hexane-2-sulfonate (*rac*-7): 6,6-Dimethyl-3-azabicyclo[3.1.0]hexane (**6**) (0.60 mL, 5.0 mmol, 1.0 equiv.) was added to a solution of NaOH (460 mg, 11.5 mmol, 2.3 equiv.), $K_2S_2O_8$ (1.50 g, 5.5 mmol, 1.1 equiv.) in water (8 mL) and MeCN (2 mL) at −5 °C under an atmosphere of nitrogen. After stirring for 1 hour at this temperature, a solution of $AgNO_3$ (43 mg, 0.25 mmol, 5 mol%) was added dropwise to the reaction mixture and the reaction mixture was left to stir at −5 °C for 1 hour. The reaction mixture was warmed to room temperature before being diluted with water (10 mL). The pH was corrected to 7, extracted with *t*BuOMe (3 x 10 mL). A solution of $NaHSO_3$ (520 mg, 5 mmol, 1.0 equiv.) in water (13.5 mL) was added to the *t*BuOMe solution and stirred for 1 hour at room temperature. The water layer was washed with *t*BuOMe (3 x 15 mL) and the aqueous layer was freeze dried to give bisulfite adduct *rac*-**7** as a white powder (730 mg, 3.42 mmol, **68%**). **^{1}H NMR (400 MHz, D_2O)** δ 4.22 (1H, s, NH), 3.40 (1H, dd, *J* = 11.5, 5.4 Hz), 3.12 (1H, d, *J* = 11.5 Hz), 1.77 (1H, d, *J* = 7.2 Hz), 1.66 (1H, t, *J* = 6.5 Hz), 1.22 – 1.12 (1H, m), 1.09 (3H, s, CH_3), 1.04 (3H, s, CH_3). Data are consistent with literature.[2]

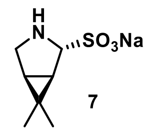

7

Sodium (1*R*,2*R*,5*S*)-6,6-dimethyl-3-azabicyclo[3.1.0]hexane-2-sulfonate (7): 6,6-Dimethyl-3-azabicyclo[3.1.0]hexane (**6**, 1.5 g, 13.49 mmol, 50 mM, 7.5 g L^{-1}) was dissolved in PBS buffer (200 mL, pH 7) in a 500 mL flask. MAO-N 401 purified enzyme (40 mg, 1.8 µM, 0.2 g L^{-1} final concentration) and catalase (Roche, 0.40 g) were added to the reaction mixture. The reaction was left on a tabletop roller for 6 h at 30 °C during which time the conversion of **6** to **3** was monitored by GC-MS. Following complete conversion of **6** to imine **3**, the pH of reaction mixture was adjusted to 8 by addition of 10 N NaOH, followed by extraction with *t*BuOMe three times (3 x 120 mL) to give an organic solution containing the desired imine **3**. The bisulfite adduct **7** was formed by the addition of $NaHSO_3$ (1.00 g, 9.7 mmol) in water (50 mL) followed by stirring of the biphasic mixture for 2 h. The resulting mixture was allowed to stand and the aqueous layer removed and washed with *t*BuOMe (3 x 100 mL). The resulting aqueous solution was submerged into liquid nitrogen, before being transferred onto a freeze drier, to give bisulfite adduct **9** (2.02 g, 9.38 mmol, **92%**) as a white powder. **^{1}H NMR (400 MHz, D_2O)** δ 4.07 (1H, s, NH), 3.20 (1H, dd, *J* = 11.5, 5.4 Hz), 2.99 (1H, d, *J* = 11.5 Hz), 1.64 (1H, d, *J* = 7.2 Hz), 1.51 (1H, t, *J* = 6.5 Hz), 1.22 – 1.12 (1H, m), 1.06 (3H, s, CH_3), 1.00 (3H, s, CH_3); **^{13}C NMR (101 MHz, D_2O)** δ 75.5, 45.9, 31.5, 29.6, 25.8, 19.0, 12.4. The NMR data are consistent with those reported in the literature.[2]

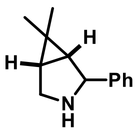

S1

6,6-Dimethyl-2-phenyl-3-azabicyclo [3.1.0] hexane (S1): PhMgBr (4 mL, 8.0 mmol, 4.0 equiv.) was added dropwise to a solution of 6,6-dimethyl-3-azabicyclo[3.1.0]hex-2-ene (*rac*-**3**) (2 mmol) in anhydrous Et_2O (4 mL) at 0 °C under an atmosphere of nitrogen. After the addition was complete the reaction mixture was allowed to warm to room temperature and left to stir for 12 hours. The reaction was quenched by the addition of a saturated solution of $NaHCO_3$ (10 mL) and extracted with Et_2O (3x 10 mL). The combined organics were dried ($MgSO_4$), filtered and concentrated *in vacuo.* Column chromatography (20% EtOAc/pet. ether) followed by a preparative TLC (EtOAc) gave 6,6-dimethyl-2-phenyl-3-azabicyclo[3.1.0]hexane (**S1**) as a pale yellow viscous oil (36 mg, 0.19 mmol, **10%** over two steps) as a ~25:1 mixture of diastereomers. **^{1}H NMR (400 MHz, $CDCl_3$)** δ 7.38 – 7.30 (5H, m, ArH), 4.88 (1H, qd, J = 6.5, 1.3 Hz, CH), 4.10 (1H, app. s, 1H), 3.41 (1H, dd, *J* = 10.8, 5.0 Hz, CH_2), 3.03 (1H, d, *J* = 10.8 Hz, CH_2), 2.41 (1H, br. s, NH), 1.49 (3H, s, CH_3), 1.50 (3H, s, CH_3), 1.30 (1H, app. d, *J* = 7.2 Hz, CH); **^{13}C NMR (101 MHz, $CDCl_3$)** δ 145.9 (ArC), 128.5 (2 x ArH), 126.4 (ArH), 125.4 (2 x ArH), 70.3 (CH), 62.0 (CH), 47.0 (CH_2), 38.2 (CH), 25.2 (2 x CH_3), 19.8 (C).†

The same procedure used to obtain the racemic standard was followed using enantioenriched 6,6-dimethyl-3-azabicyclo[3.1.0]hex-2-ene (**3**) (4.58 mmol) obtained from the large scale biotransformation.

† ^{1}H and ^{13}C NMR spectra of **S1** contain an unidentified impurity.

Table S2. Optimization of dehydration conditions of formamide **11** to isocyanide **4a**.

Entry	Dehydration agent	equiv.	Base	equiv.	Solvent	T (°C)	[conc]	t (h)	Yield (%)[e]	Ref.
1	T3P	1.9	Et_3N	8	EtOAc	55	0.1	6	41	4
2	Burgess	1.5	-	-	DCM	40	0.04	1.2	51	5
3	Burgess	1.5	-	-	DCM	40	0.04	1.2	58[a]	5
4	Burgess	1.05	-	-	DCM	40	0.04	2	70[b]	5
5	$EtOPOCl_2$	1.2	-	-	DCM	RT	0.6	16	n.d.[b]	6
6	TsCl	1.5	pyridine	3	DCM	RT	0.25	3.5	51[b]	7
7	TsCl	2.5	pyridine	3	DCM	RT	0.1	-	n.d.[b]	7
8	TsCl	1.5	Et_3N	5	THF	RT	0.25	2	n.d.[b]	7
9	triphosgene	0.35	Et_3N	10	DCM	-78	0.05	1.5	64[b]	
10	triphosgene	0.5	Et_3N	10	DCM	-78	0.05	2	75[b]	
11	triphosgene	0.66	Et_3N	10	DCM	-78	0.05	1.2	84[c]	
12	triphosgene	1	Et_3N	10	DCM	-78	0.05	2	26[d]	
13	$POCl_3$	1	Et_3N	6	DCM	-78	0.1	2	35[b,d]	
14	TFAA	1.9	Et_3N	8	DCM	0	0.1	3	65	
15	TFAA	1.2	Et_3N	8	DCM	0	0.1	2.5	53	
16	TFAA	2.4	Et_3N	8	DCM	0	0.1	2.5	46	
17	TFAA	1.9	Et_3N	8	THF	0	0.1	3	85	
18	TFAA	1.9	*i*Pr_2NEt	8	DCM	0	0.1	3	66	
19	TFAA	1.9	pyridine	8	DCM	0	0.1	3	29	

[a] crude product loaded onto silica gel flash column directly. [b] incomplete conversion observed by TLC analysis. [c] >gram scale reaction. [d] significant degradation observed. [e] isolated yield of purified (>95%) product as confirmed by ^{1}H NMR analysis. T3P = propanephosphonic anhydride; Burgess reagent = methyl *N*-(triethylammoniumsulfonyl)carbamate; n.d. = not determined; TFAA = trifluoroacetic anhydride.

Procedure for the streamlined synthesis of isocyanide 4a from 5:

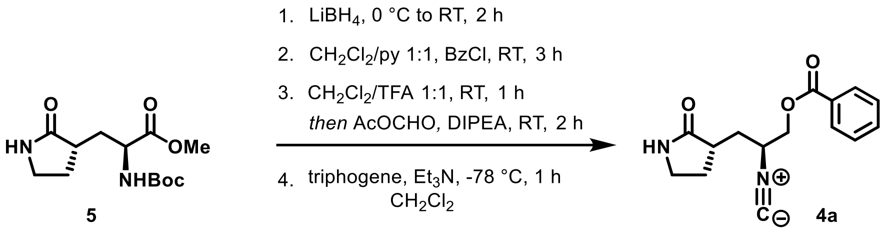

To a solution of Boc-amino methyl ester **5** (5.00 g, 17.46 mmol, 1.0 equiv.) in dry THF (140 mL) at 0 °C under N_2 atmosphere, $LiBH_4$ (2 M in THF, 21.8 mL, 43.6 mmol, 2.5 equiv.) was added dropwise. The reaction mixture was kept at 0 °C for 10 minutes, then allowed to warm to room temperature and stirred for 2.5 h. Upon full conversion (TLC: 6% MeOH in CH_2Cl_2, R_f (prod) = 0.35, ninhydrin stain) the reaction mixture was cooled down to 0 °C and quenched by dropwise addition of a solution of NH_4Cl (50 mL, 0.5 M) until gas evolution ceased. The reaction mixture was further diluted with water (100 mL), and the aqueous phase was repeatedly extracted with ethyl acetate (6 x 150 mL). The combined organic extracts were dried (Na_2SO_4), filtered, and concentrated *in vacuo* to a total volume of approximately 100 mL. The solution was then stored at 5 °C for 2 hours and allowed to precipitate. After filtration, the crude Boc-amino alcohol **9** (3.81 g, 15.58 mmol, 1 equiv.) was further dried *in vacuo* at 40 °C for 2 hours and obtained as a white foamy solid. This material was redissolved in dry CH_2Cl_2 /pyridine 1:1 (147 mL) and cooled to 0 °C to form a clear solution. Benzoyl chloride (2.0 mL, 16.9 mmol, 1.15 equiv.) was added dropwise

under stirring and the reaction mixture was brought back to room temperature after 10 minutes. TLC (3% MeOH in CH_2Cl_2, R_f (prod) = 0.24) indicated full conversion after 3 h, after which the solvents were removed *in vacuo* by co-evaporation with chloroform (2x). The concentrate was diluted with additional CH_2Cl_2 (100 mL) and washed successively with 0.25 M HCl (150 mL), a saturated solution of $NaHCO_3$ (150 mL), water (150 mL) and brine (250 mL), after which the organic layer was dried ($MgSO_4$), filtered, and concentrated *in vacuo*. The crude benzoate **10** (5.28 g, 14.57 mmol, 1 equiv.) was afforded as viscous, slightly yellowish oil, and directly redissolved in dry CH_2Cl_2/TFA 1:1 (80 mL) to yield a clear, yellow solution. The reaction mixture was stirred for 2 h at room temperature, after which the solvents were removed *in vacuo* by co-evaporation with toluene (2x) and chloroform (2x). The yellow to orange oily residue was redissolved in dry CH_2Cl_2 (40 mL) and freshly prepared acetic formic anhydride (4.7 mL, 58.2 mmol, 4.0 equiv.) was added. The reaction mixture was then cooled down to 0 °C, followed by dropwise addition of DIPEA (6.3 mL, 36.4 mmol, 2.5 equiv.). Thereafter the reaction mixture was brought back to room temperature and stirred for 2 h. Upon full conversion (as indicated by TLC) the reaction mixture was diluted with additional CH_2Cl_2 (60 mL) and washed successively with 0.25 M HCl (100 mL), a saturated solution of $NaHCO_3$ (100 mL), water (100 mL) and brine (100 mL), after which the organic phase was dried (Na_2SO_4), filtered, and concentrated *in vacuo*. The crude formamide **11** (3.38 g, 11.65 mmol, 1 equiv.) was obtained as a sticky off-white solid and redissolved in dry CH_2Cl_2 (108 mL) and Et_3N (13 mL, 93.1 mmol, 10 equiv.). The solution was cooled to -78 °C before triphosgene (2.30 g, 7.74 mmol, 0.66 equiv.) was quickly added. The reaction mixture was stirred for 2 h, then carefully quenched with water (60 mL) and a saturated solution of $NaHCO_3$ (60 mL). After slowly warming to room temperature, the layers were separated and the aqueous phase was reextracted with CH_2Cl_2 (4 x 100 mL). The combined organic extracts were dried (Na_2SO_4), concentrated *in vacuo* and finally subjected to column chromatography over silica gel (3% MeOH in CH_2Cl_2) to afford isocyanide **4a** (2.78 g, 10.19 mmol, **58%**) as a crystalline white solid. **R_f**: 0.42 (4% MeOH in CH_2Cl_2, $KMnO_4$ stain).

Procedure for streamlined multicomponent synthesis of nirmatrelvir (1):

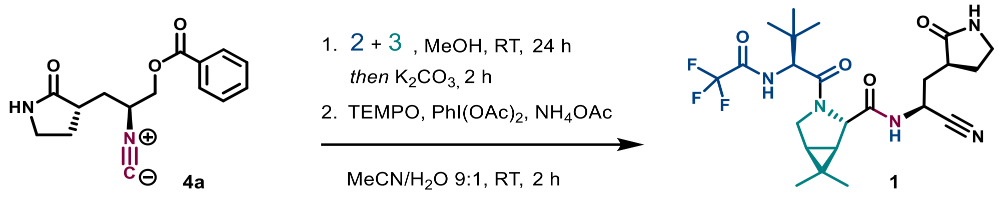

The imine bisulfite adduct **7** (344 mg, 1.62 mmol, 2.2 equiv.) was dissolved in 0.5 M NaOH (aq.) (8 mL) and stirred at room temperature for 1 h. The resulting free imine **3** was extracted with CH_2Cl_2 (4 x 8 mL), dried (Na_2SO_4), and filtered, after which the carboxylic acid **2** (367 mg, 1.62 mmol, 2.2 equiv.) was added to the combined organic extracts and concentrated *in vacuo*. A solution of the isocyanide **4a** (200 mg, 0.73 mmol, 1.0 equiv.) in MeOH (1.8 mL) was added, and the reaction mixture was stirred at room temperature for 48 h. Upon consumption of the isocyanide (TLC: 4% MeOH in CH_2Cl_2, R_f (prod) = 0.35), the crude U3CR adduct **12** was further diluted with MeOH (30 mL) and K_2CO_3 (355 mg, 2.57 mmol, 3.5 eq.) was added. The reaction mixture was stirred at room temperature for 1.5 h until full conversion of **12** was observed by TLC (5% MeOH in CH_2Cl_2, R_f (prod) = 0.22, $KMnO_4$ or *p*-anisaldehyde stain). The reaction mixture was then first quenched with a saturated solution of NH_4Cl (30 mL), and the aqueous layer extracted with ethyl acetate (4 x 30 mL), after which the combined organic layers were collected, dried (Na_2SO_4), and concentrated *in vacuo*. The crude alcohol **13** was redissolved in CH_3CN/H_2O 9:1 (7.3 mL) and TEMPO (5.7 mg, 0.04 mmol, 0.05 equiv.), $PhI(OAc)_2$ (520 mg, 1.62 mmol, 2.2 equiv.) and NH_4OAc (226 mg, 2.94 mmol, 4.0 equiv.) were successively added. The yellow solution was then stirred at room temperature for 2 h, the conversion carefully monitored by TLC (5% MeOH in CH_2Cl_2, R_f (prod) = 0.42, *p*-anisaldehyde stain). Upon completion, the reaction mixture was quenched with a 35 wt% $Na_2S_2O_3$ solution (10 mL), and further diluted with water (10 mL) after which the aqueous layer was extracted with ethyl acetate (4 x 20 mL). The combined organic layers were then washed with 0.25 M HCl (100 mL), sat. aq. $NaHCO_3$ (100 mL), water (100 mL) and brine (100 mL), dried (Na_2SO_4), filtered, concentrated in *vacuo* and subjected to column chromatography over silica gel (2-4% MeOH in CH_2Cl_2) to afford **1** (nirmatrelvir) (254 mg, 0.51 mmol, **70%**, *dr* >25:1) as a foamy white solid. **R_f**: 0.4 (5% MeOH in CH_2Cl_2).

X-Ray crystal structure of 4a

Single block-like crystals were obtained by cooling a solution of isocyanide **4a** in boiling acetone to room temperature. A suitable crystal was selected, mounted, and cooled to 100 K.

The total exposure time was 2.72 hours. The frames were integrated with the Bruker SAINT software package using a narrow-frame algorithm. The integration of the data using a monoclinic unit cell yielded a total of 12376 reflections to a maximum θ angle of 72.30° (0.81 Å resolution), of which 2678 were independent (average redundancy 4.621, completeness = 99.4%, R_{int} = 2.37%, R_{sig} = 1.80%) and 2652 (99.03%) were greater than 2σ(F^2). The final cell constants of a = 7.6150(2) Å, b = 6.59060(10) Å, c = 13.9974(3) Å, β = 102.1910(10)°, volume = 686.65(3) $Å^3$, are based upon the refinement of the XYZ-centroids of 9927 reflections above 20 σ(I) with 6.460° < 2θ < 144.6°.Data were corrected for absorption effects using the multi-Scan method (SADABS). The ratio of minimum to maximum apparent transmission was 0.685.

The structure was solved and refined using the Bruker SHELXTL Software Package, using the space group P 1 21 1, with Z = 2 for the formula unit, $C_{15}H_{16}N_2O_3$.[8,9] The final anisotropic full-matrix least-squares refinement on F^2 with 181 variables converged at R1 = 2.63%, for the observed data and wR2 = 6.71% for all data. The goodness-of-fit was 1.110. The largest peak in the final difference electron density synthesis was 0.231 $e^-/Å^3$ and the largest hole was -0.188 $e^-/Å^3$ with an RMS deviation of 0.036 $e^-/Å^3$. Based on the final model, the calculated density was 1.317 g/cm^3 and F(000), 288 e^-.

Analysis and visualization of the final absolute structure was performed using PLATON.[10,11] The results indicate that the absolute stereochemistry had been correctly assigned based on the Flack parameter: x = 0.08(4).[12]

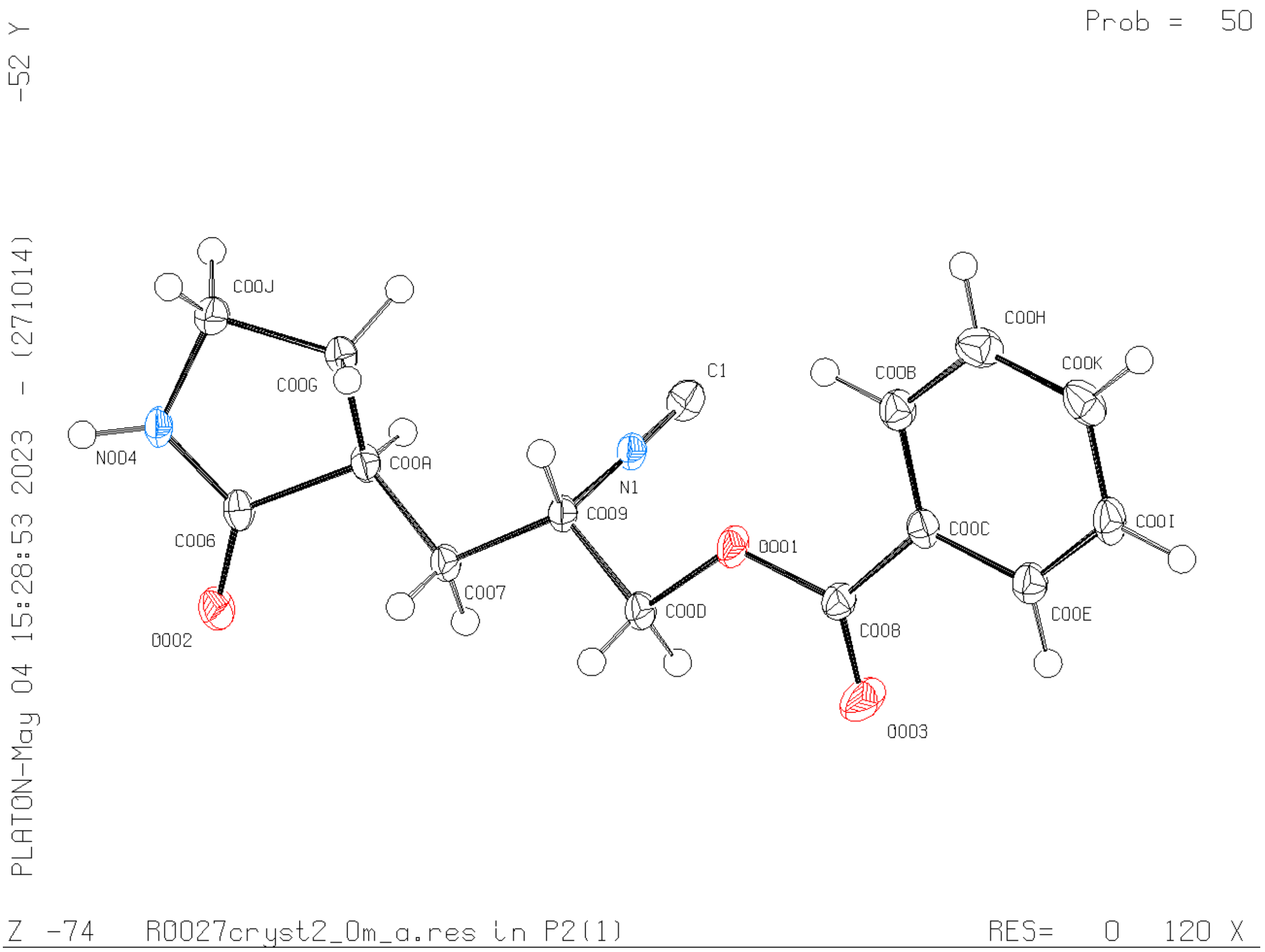

Figure S3. ORTEP plot for isocyanide **4a** at 50% ellipsoid contour probability level.

Table S3. Crystal and refinement data for RO027cryst2.

**Identification code**	RO027cryst2	
**Chemical formula**	$C_{15}H_{16}N_2O_3$	
**Formula weight**	272.30 g/mol	
**Temperature**	100(2) K	
**Wavelength**	1.54178 Å	
**Crystal system**	monoclinic	
**Space group**	P 1 21 1	
**Unit cell dimensions**	a = 7.6150(2) Å	α = 90°
	b = 6.59060(10) Å	β = 102.1910(10)°
	c = 13.9974(3) Å	γ = 90°
**Volume**	686.65(3) $Å^3$	
**Z**	2	
**Density (calculated)**	1.317 g/cm^3	
**Absorption coefficient**	0.762 mm^{-1}	
**F(000)**	288	
**Theta range for data collection**	3.23 to 72.30°	
**Index ranges**	-9<=h<=9, -8<=k<=8, -17<=l<=17	
**Reflections collected**	12376	
**Independent reflections**	2678 [R(int) = 0.0237]	
**Coverage of independent reflections**	99.4%	
**Absorption correction**	Multi-Scan	
**Structure solution technique**	direct methods	
**Structure solution program**	XT, VERSION 2018/2	
**Refinement method**	Full-matrix least-squares on F2	
**Refinement program**	SHELXL-2019/1 (Sheldrick, 2019)	
**Function minimized**	Σ w(Fo2 - Fc2)2	
**Data / restraints / parameters**	2678 / 1 / 181	
**Goodness-of-fit on F2**	1.110	
**Final R indices**	2652 data; I>2σ(I)	R1 = 0.0263, wR2 = 0.0670
	all data	R1 = 0.0265, wR2 = 0.0671
**Weighting scheme**	$w=1/[\sigma^2(F_o^2)+(0.0363P)^2+0.1107P]$ where $P=(F_o^2+2F_c^2)/3$	
**Absolute structure parameter**	0.08(4)	
**Largest diff. peak and hole**	0.231 and -0.188 $eÅ^{-3}$	
**R.M.S. deviation from mean**	0.036 $eÅ^{-3}$	

Table S4. Atomic coordinates and equivalent isotropic atomic displacement parameters (Å^2) for RO027cryst2.

U(eq) is defined as one third of the trace of the orthogonalized U_{ij} tensor.

	x/a	y/b	z/c	U(eq)
**O001**	0.62769(16)	0.55823(17)	0.42705(8)	0.0215(3)
**O002**	0.98061(17)	0.65996(19)	0.90290(8)	0.0240(3)
**O003**	0.5260(2)	0.8474(2)	0.35162(9)	0.0333(3)
**N1**	0.97943(18)	0.5757(2)	0.54744(9)	0.0175(3)
**N004**	0.03064(17)	0.3161(2)	0.90622(9)	0.0178(3)
**C1**	0.1088(2)	0.5993(3)	0.51848(13)	0.0241(4)
**C006**	0.0020(2)	0.4979(2)	0.86321(11)	0.0166(3)
**C007**	0.8495(2)	0.5985(2)	0.69205(11)	0.0170(3)
**C008**	0.5574(2)	0.6685(3)	0.34861(12)	0.0171(3)
**C009**	0.8177(2)	0.5421(2)	0.58392(11)	0.0163(3)
**C00A**	0.0001(2)	0.4724(2)	0.75456(11)	0.0163(3)
**C00B**	0.5754(2)	0.3444(3)	0.25497(12)	0.0194(3)
**C00C**	0.51967(19)	0.5458(3)	0.25743(11)	0.0167(3)
**C00D**	0.6633(2)	0.6585(2)	0.52145(11)	0.0186(3)
**C00E**	0.4219(2)	0.6367(3)	0.17274(12)	0.0194(3)
**C00G**	0.9839(2)	0.2412(3)	0.73986(11)	0.0180(3)
**C00H**	0.5335(2)	0.2371(3)	0.16779(13)	0.0244(4)
**C00I**	0.3775(2)	0.5273(3)	0.08665(12)	0.0224(4)
**C00J**	0.0609(2)	0.1524(3)	0.84148(11)	0.0199(3)
**C00K**	0.4336(2)	0.3271(3)	0.08422(13)	0.0248(4)

NMR Spectra

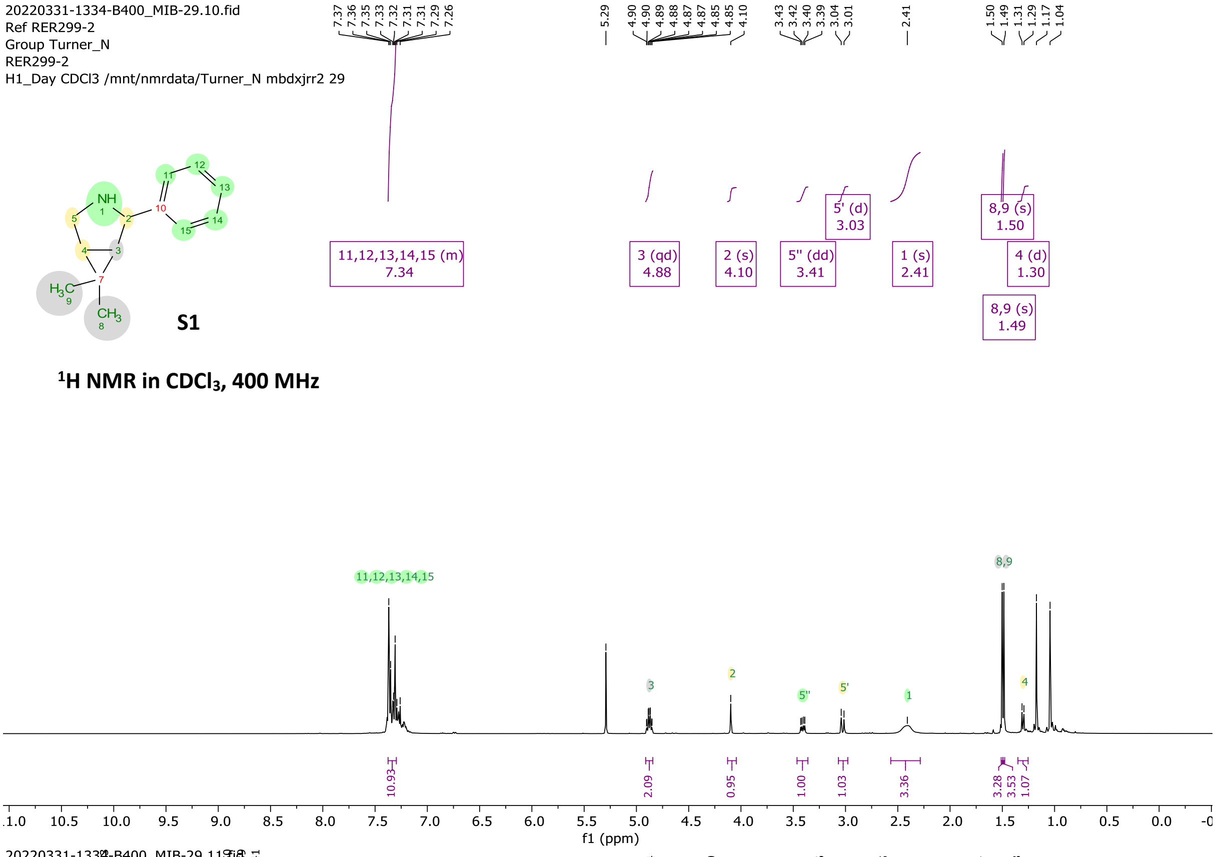

S1

^{1}H NMR in $CDCl_3$, 400 MHz

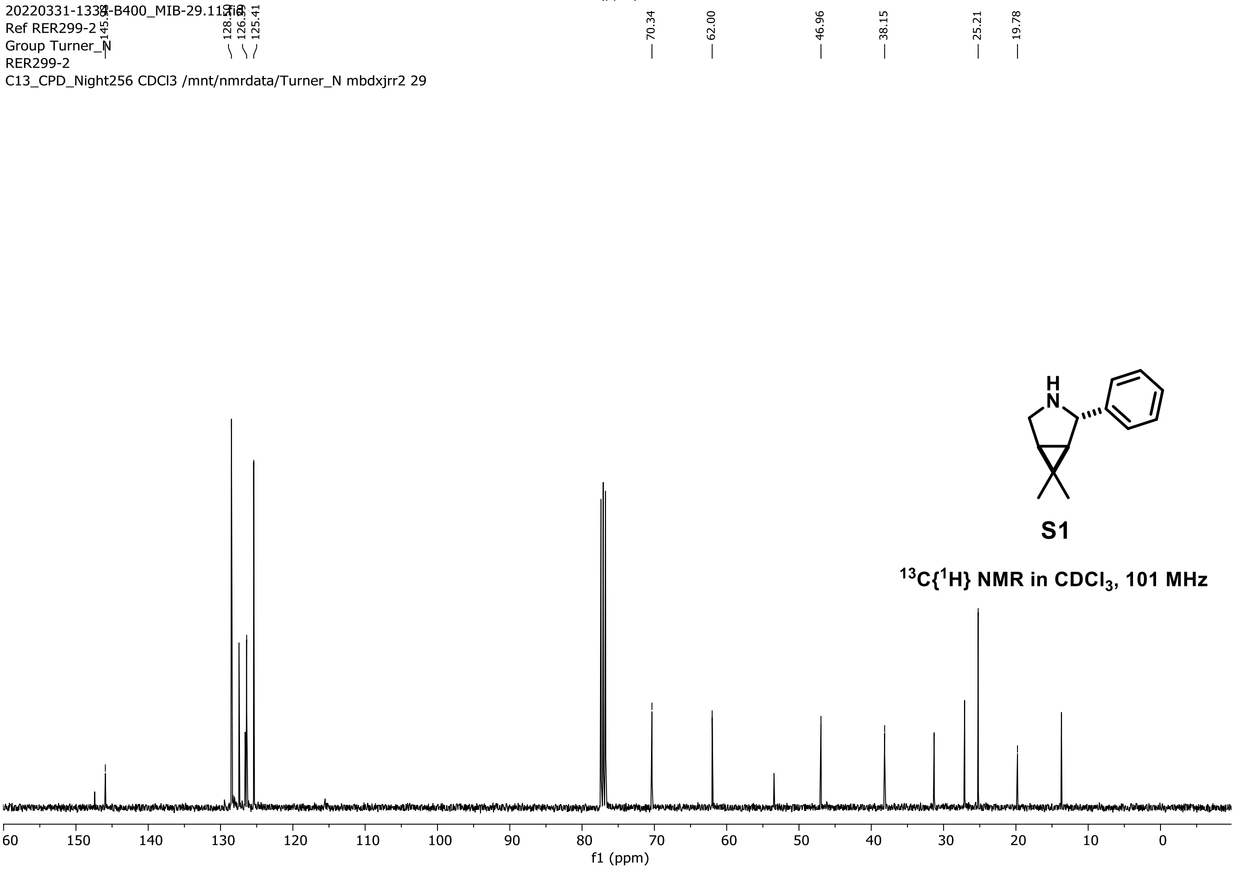

S1

$^{13}C\{^{1}H\}$ NMR in $CDCl_3$, 101 MHz

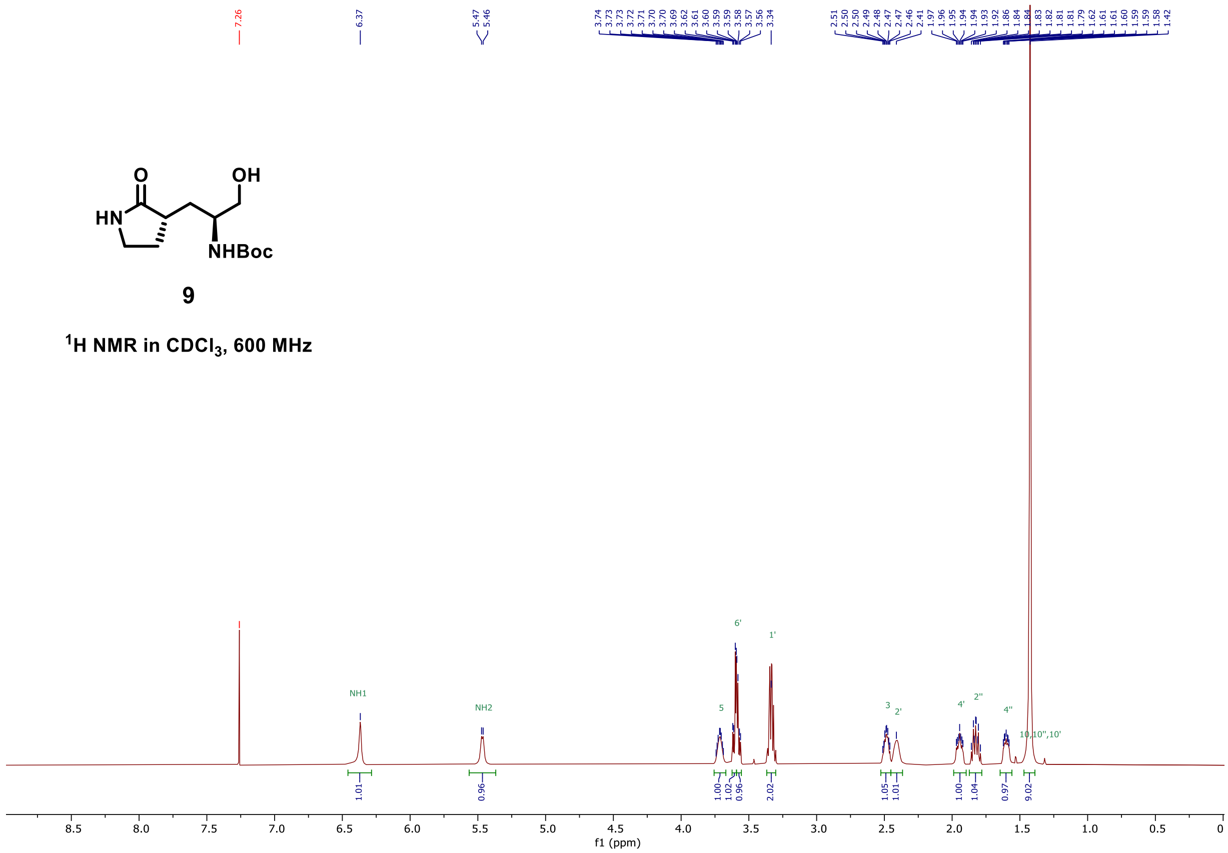

9

^{1}H NMR in $CDCl_3$, 600 MHz

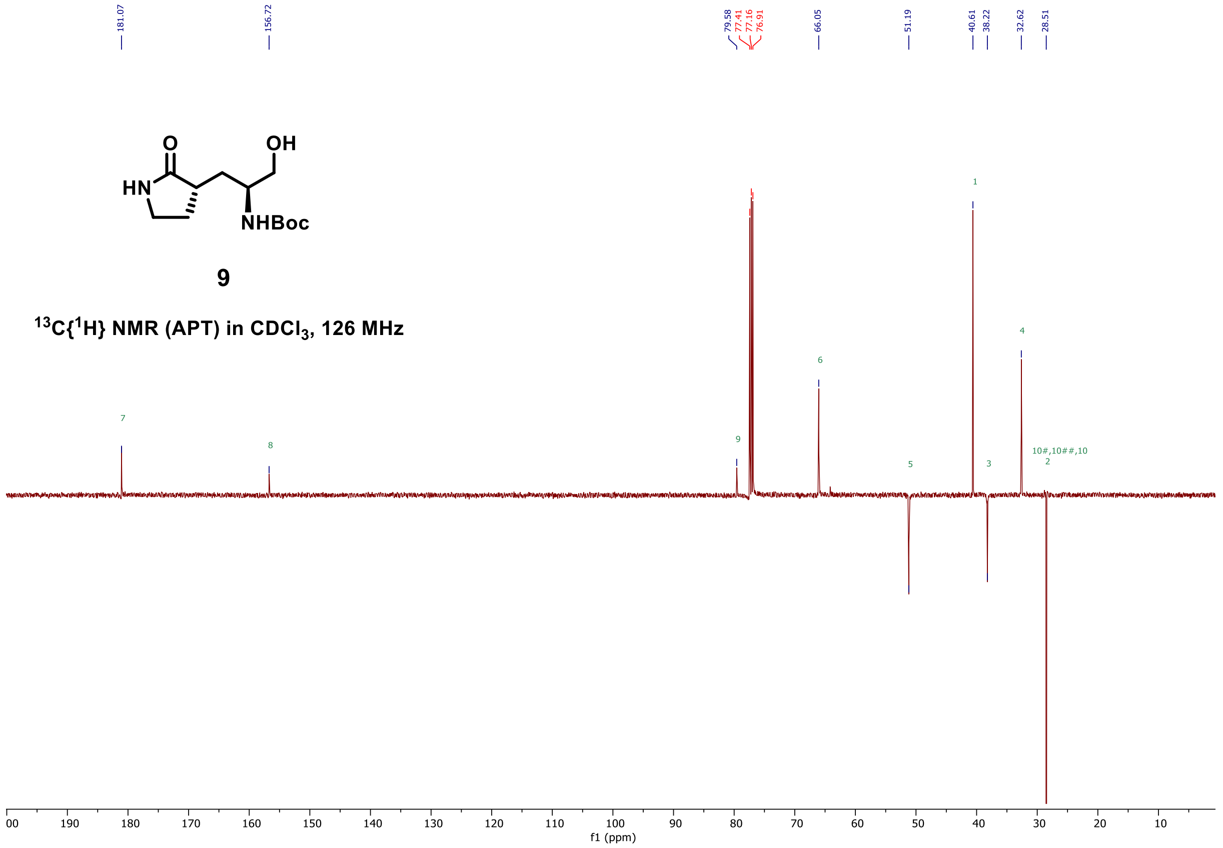

9

$^{13}C\{^{1}H\}$ NMR (APT) in $CDCl_3$, 126 MHz

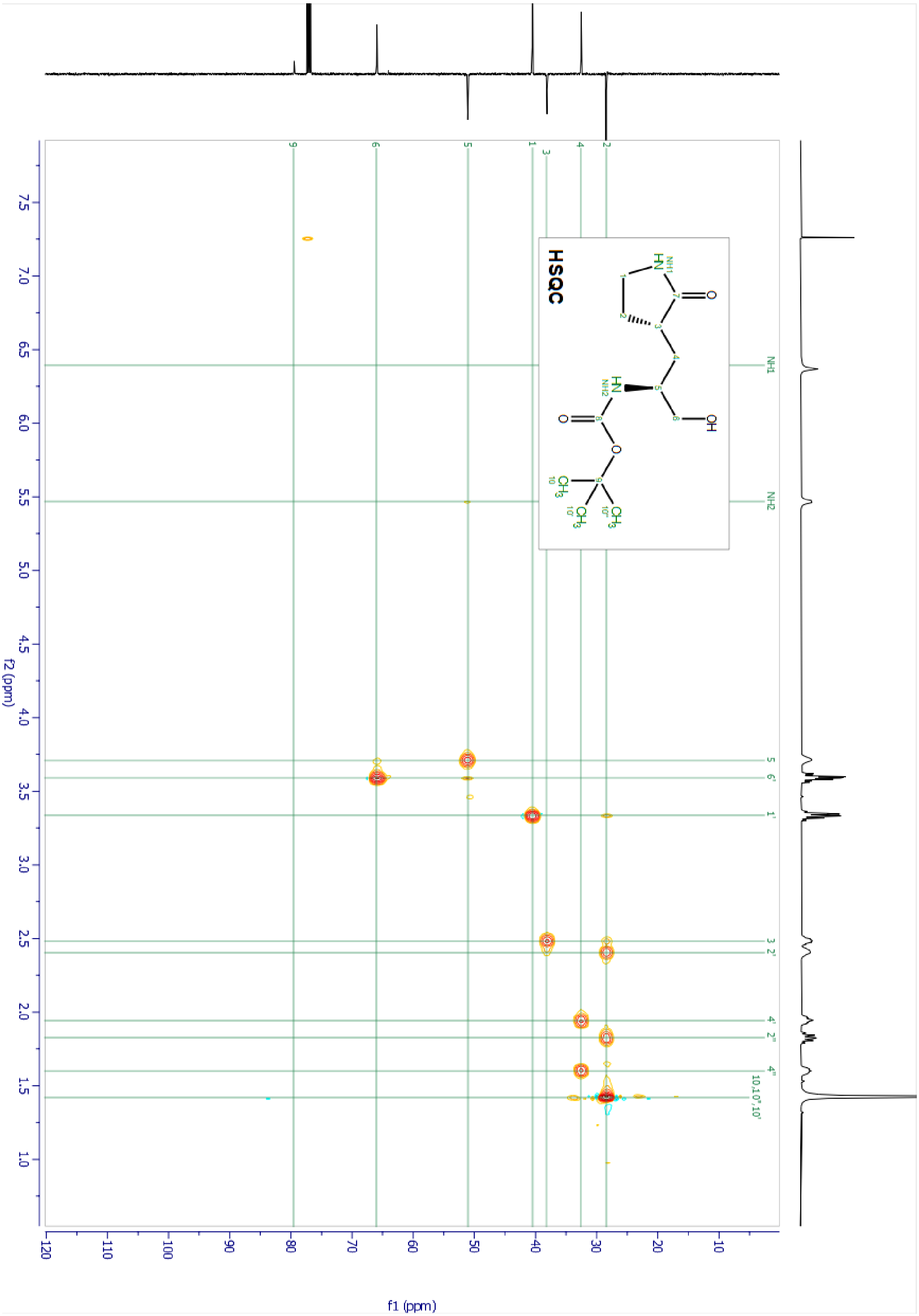
HSQC
f1 (ppm)
f2 (ppm)

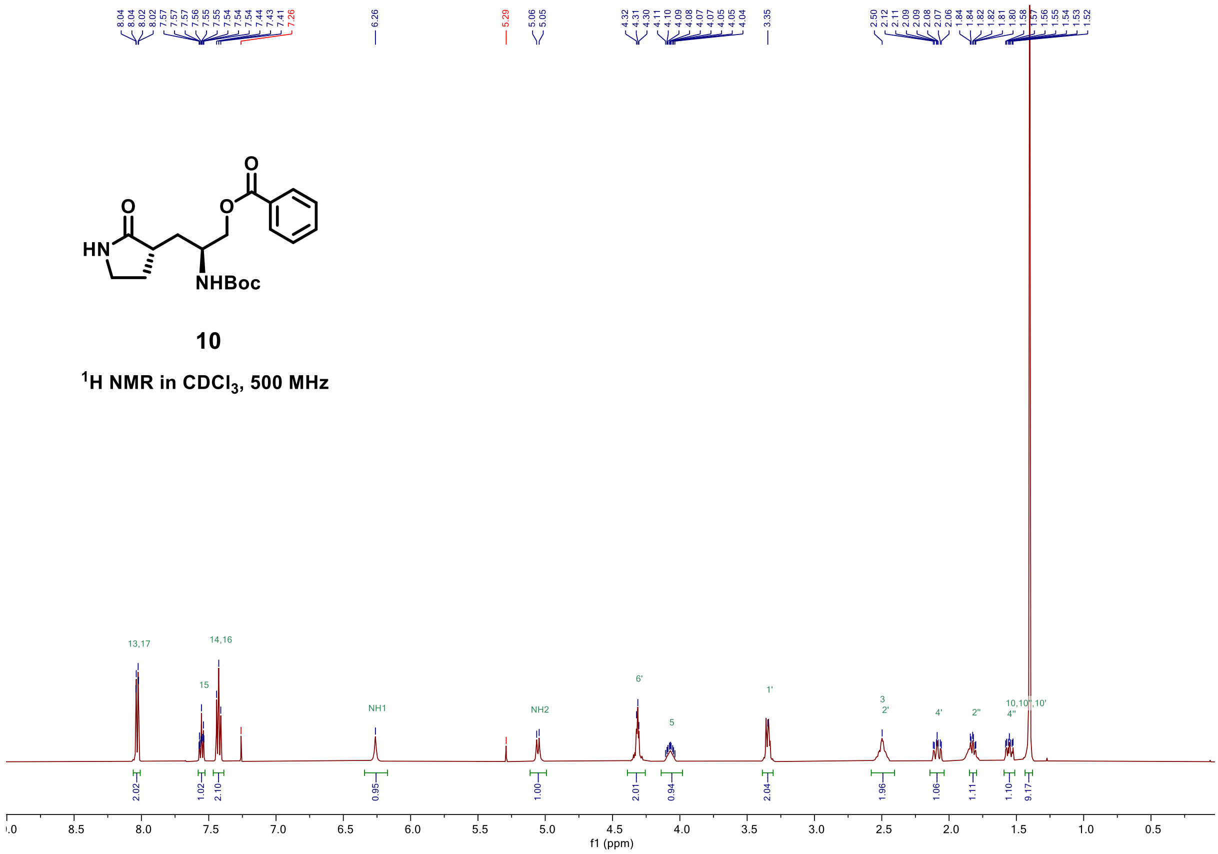

10

^{1}H NMR in $CDCl_3$, 500 MHz

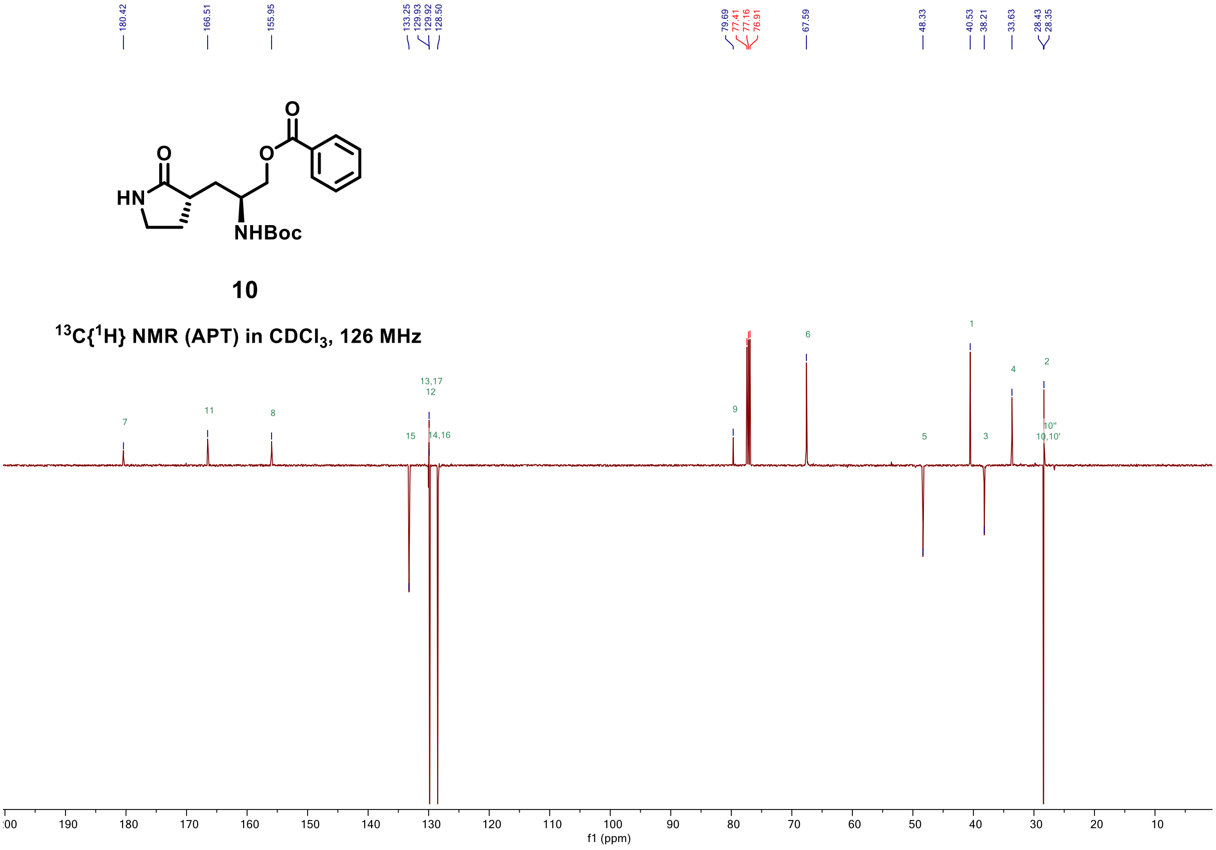

10

$^{13}C\{^{1}H\}$ NMR (APT) in $CDCl_3$, 126 MHz

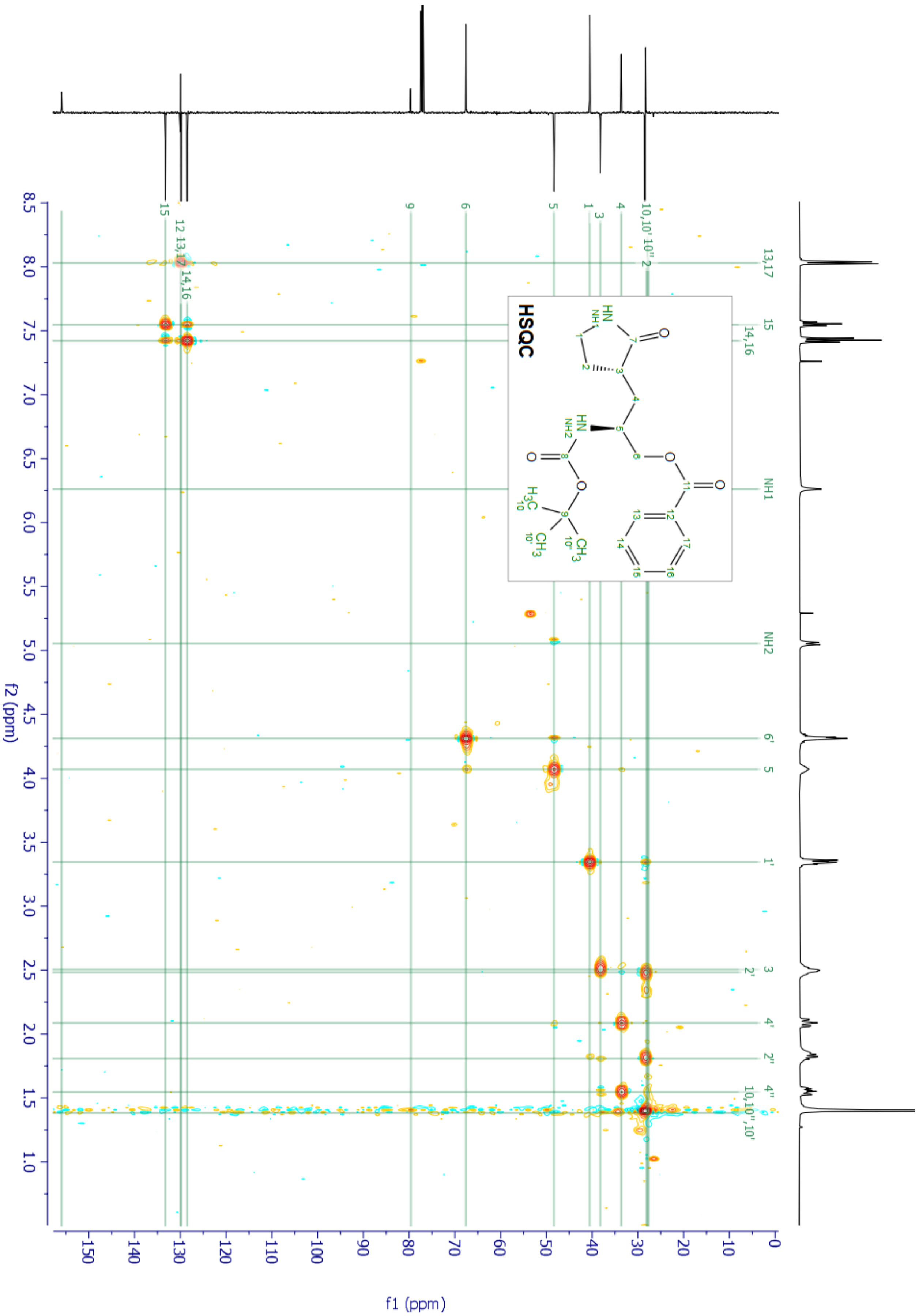
HSQC
f1 (ppm)
f2 (ppm)

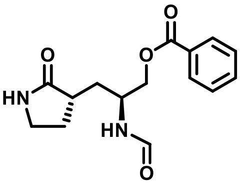

11

^{1}H NMR in $CDCl_3$, 500 MHz

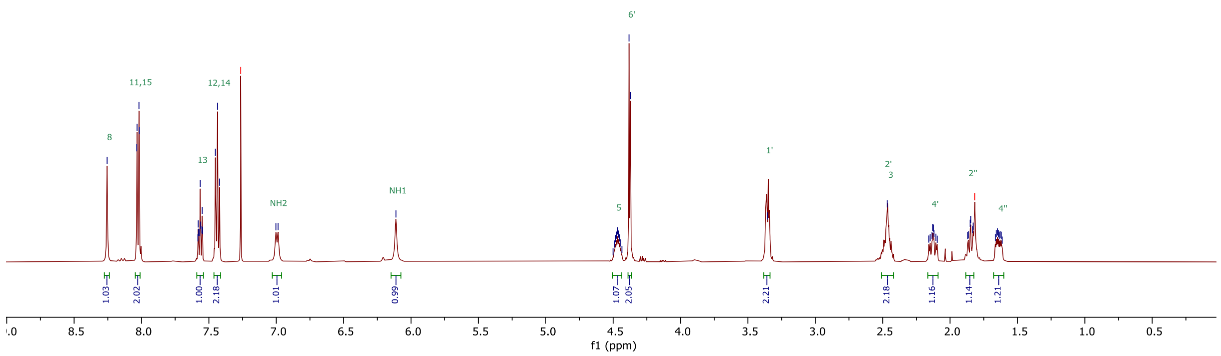

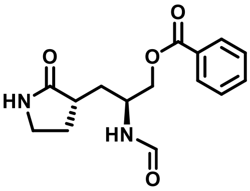

11

$^{13}C\{^1H\}$ NMR (APT) in $CDCl_3$, 126 MHz

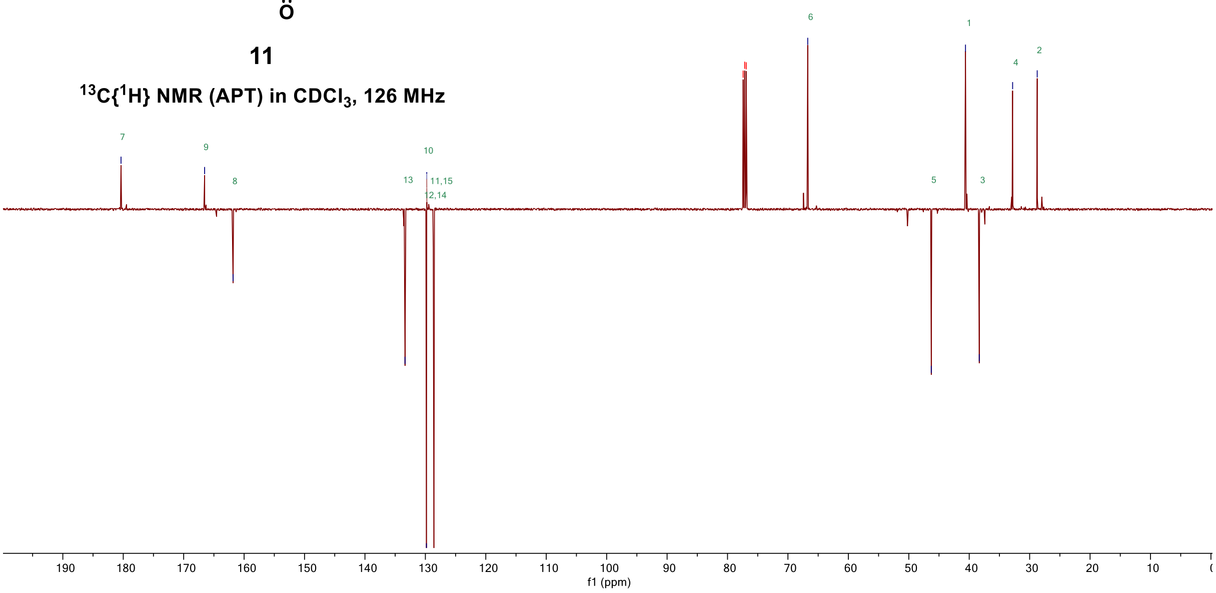

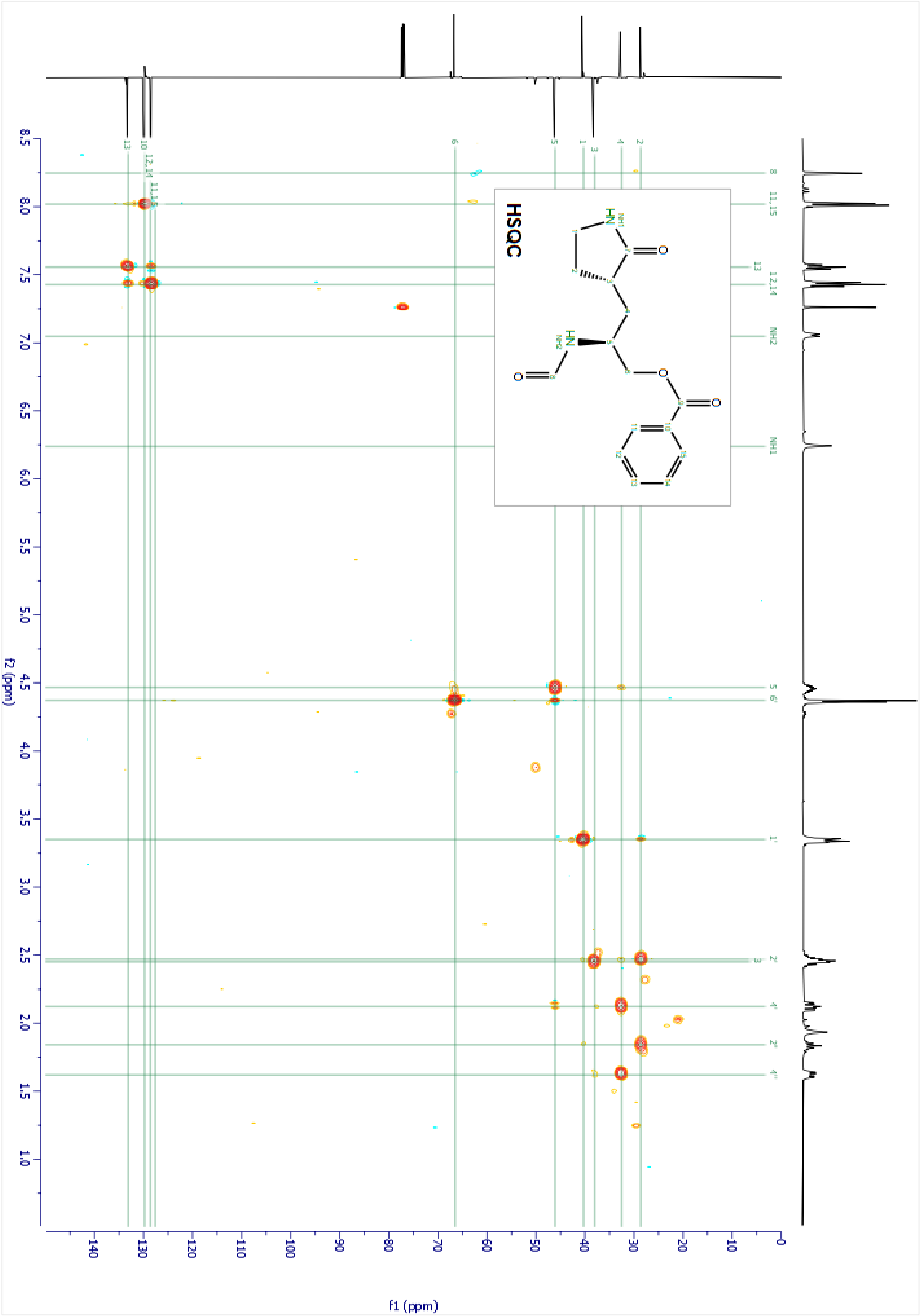
HSQC
f1 (ppm)
f2 (ppm)

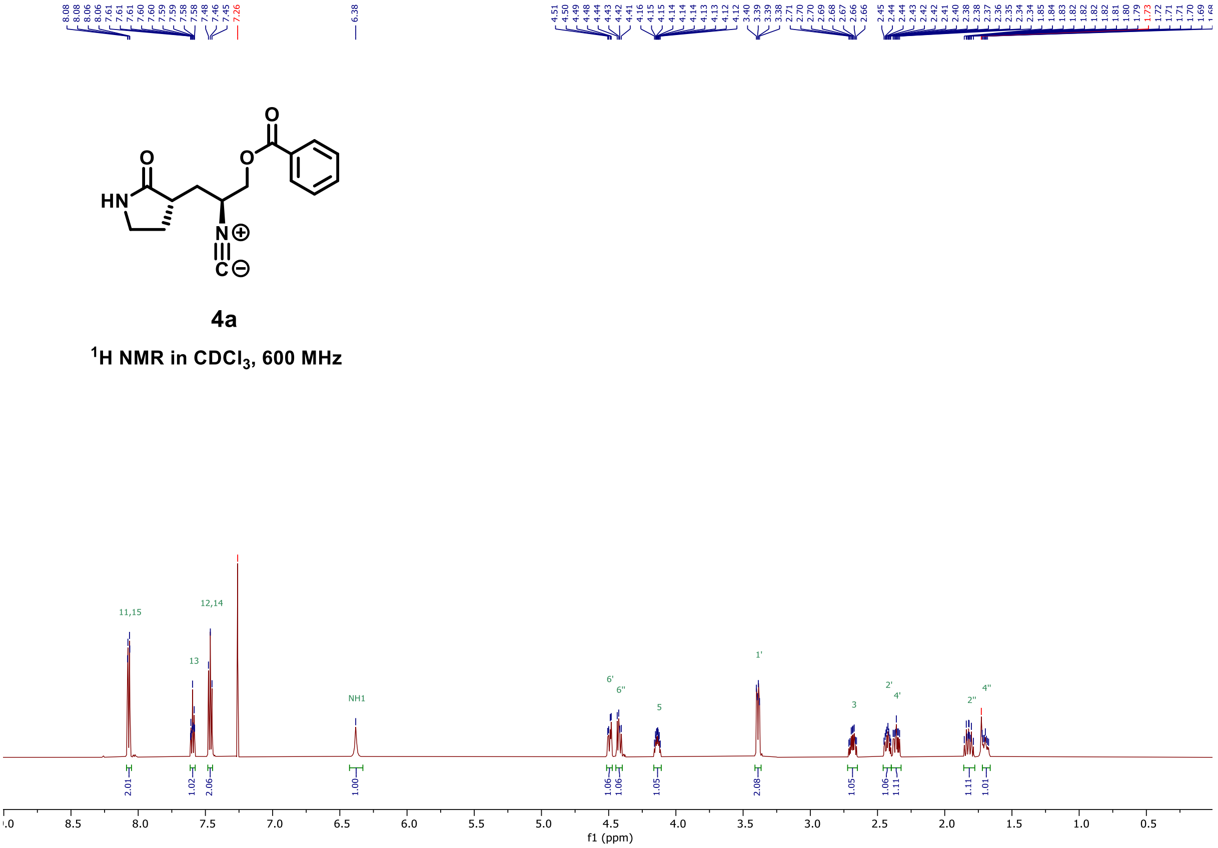

4a

^{1}H NMR in $CDCl_3$, 600 MHz

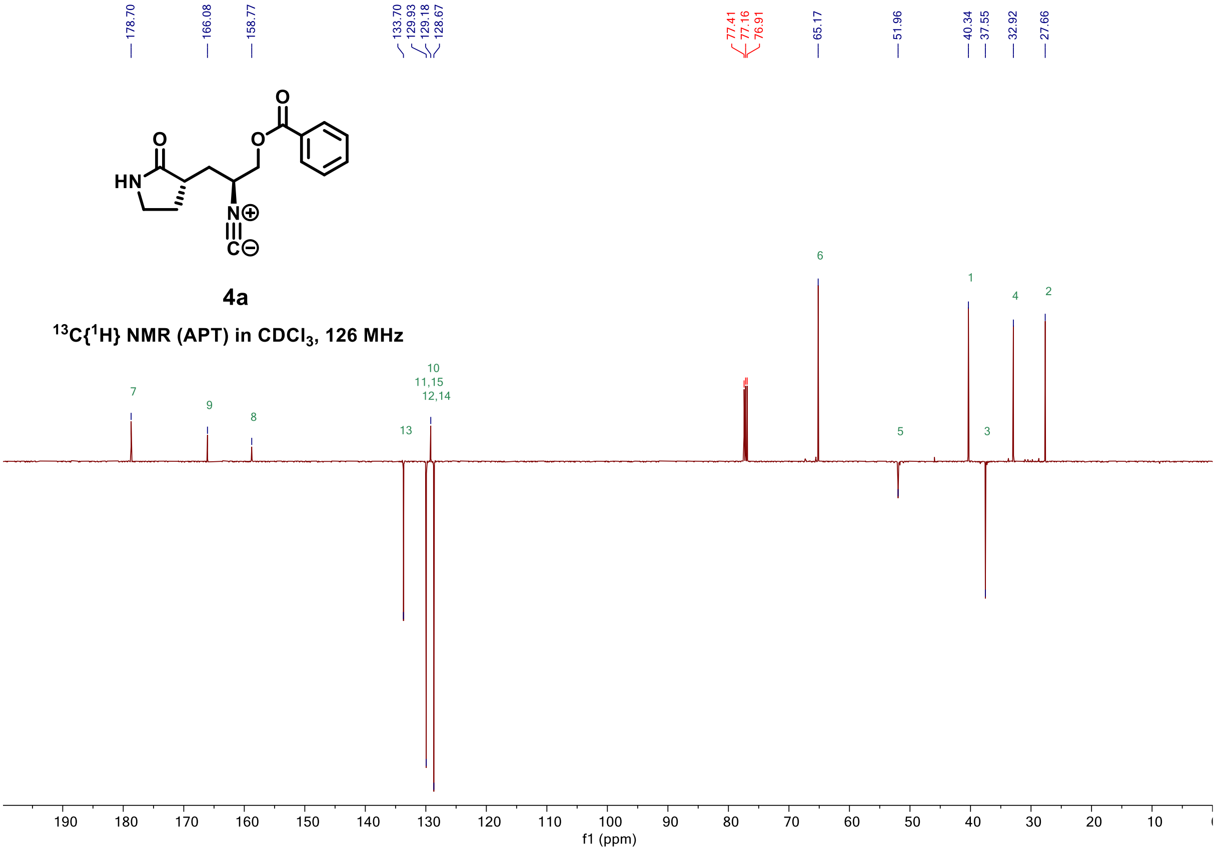

4a

^{13}C{^{1}H} NMR (APT) in $CDCl_3$, 126 MHz

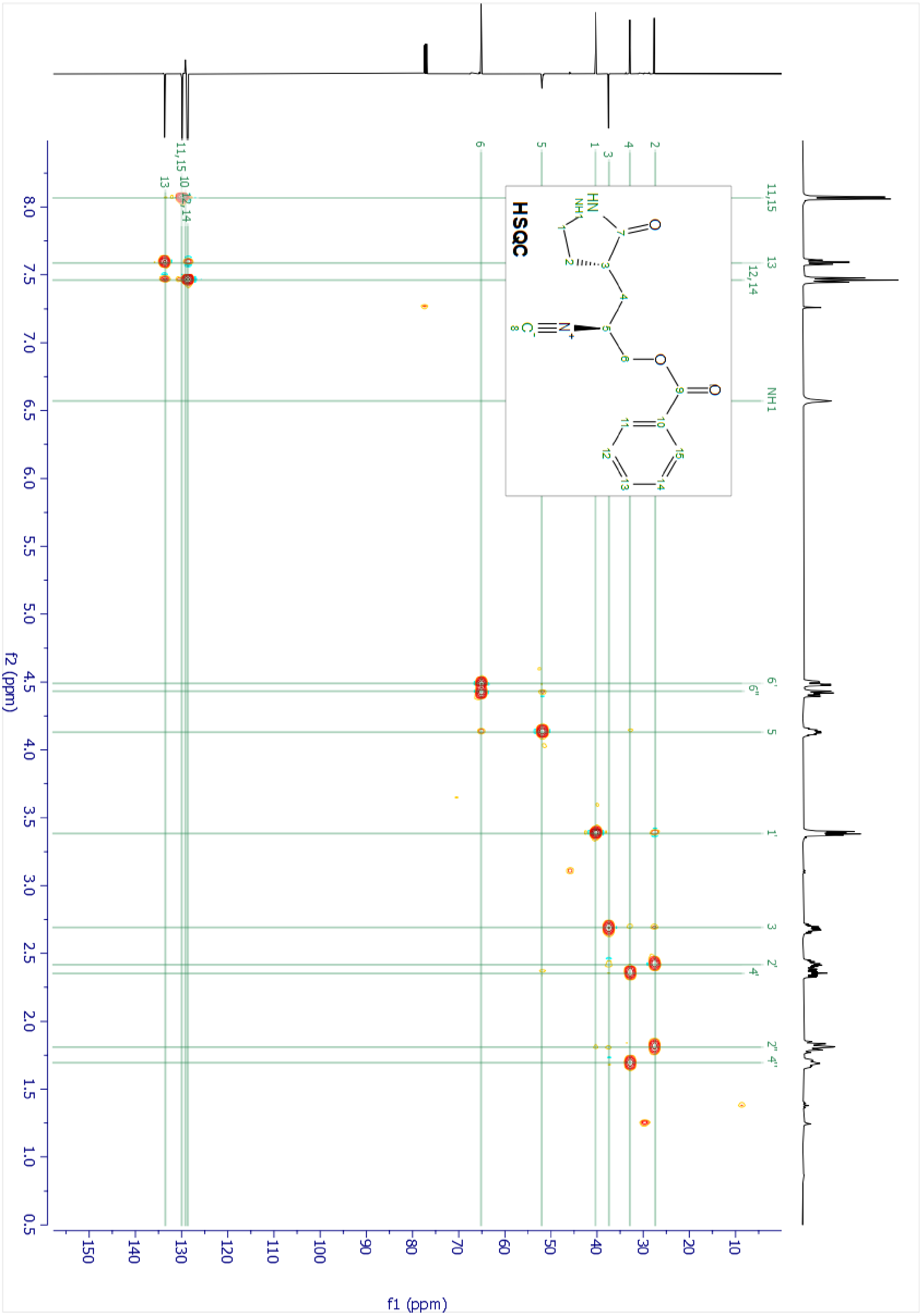
HSQC
f1 (ppm)
f2 (ppm)

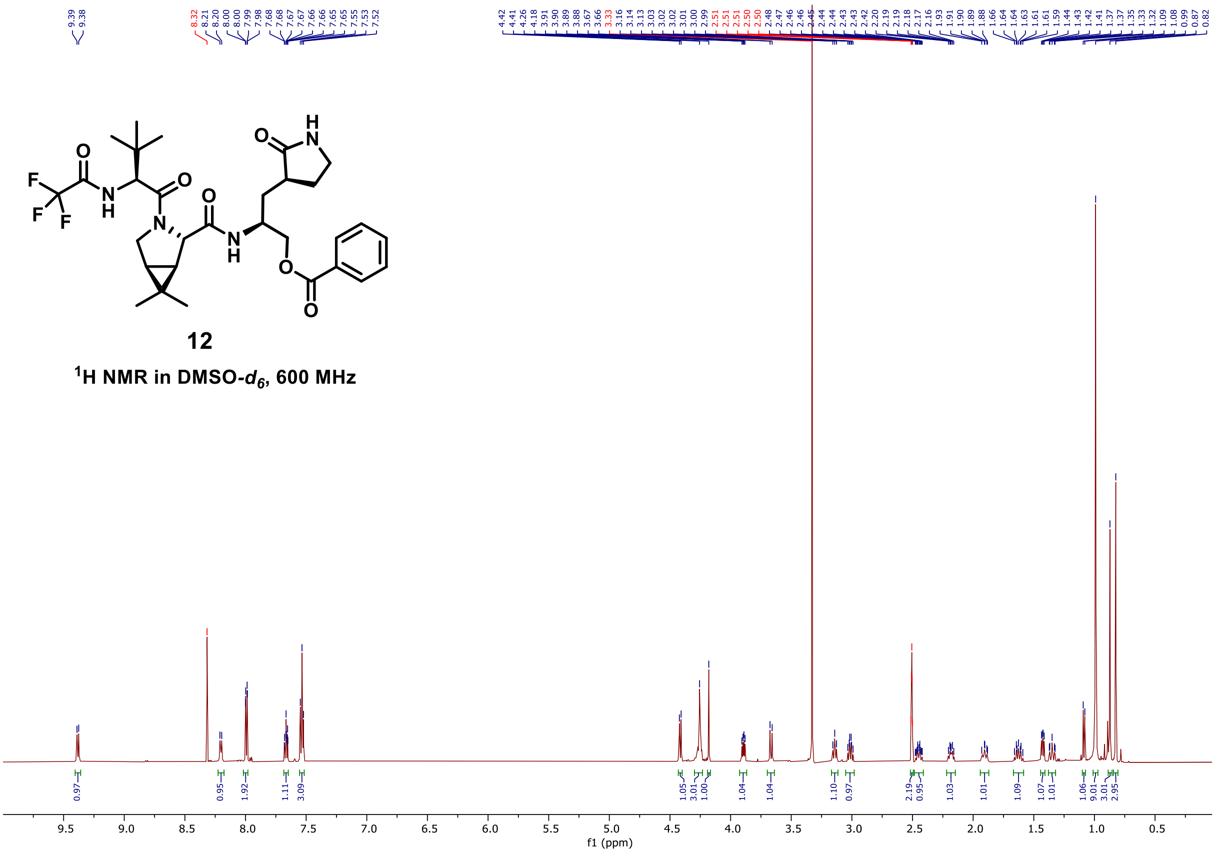
12
1H NMR in DMSO-d6, 600 MHz
f1 (ppm)

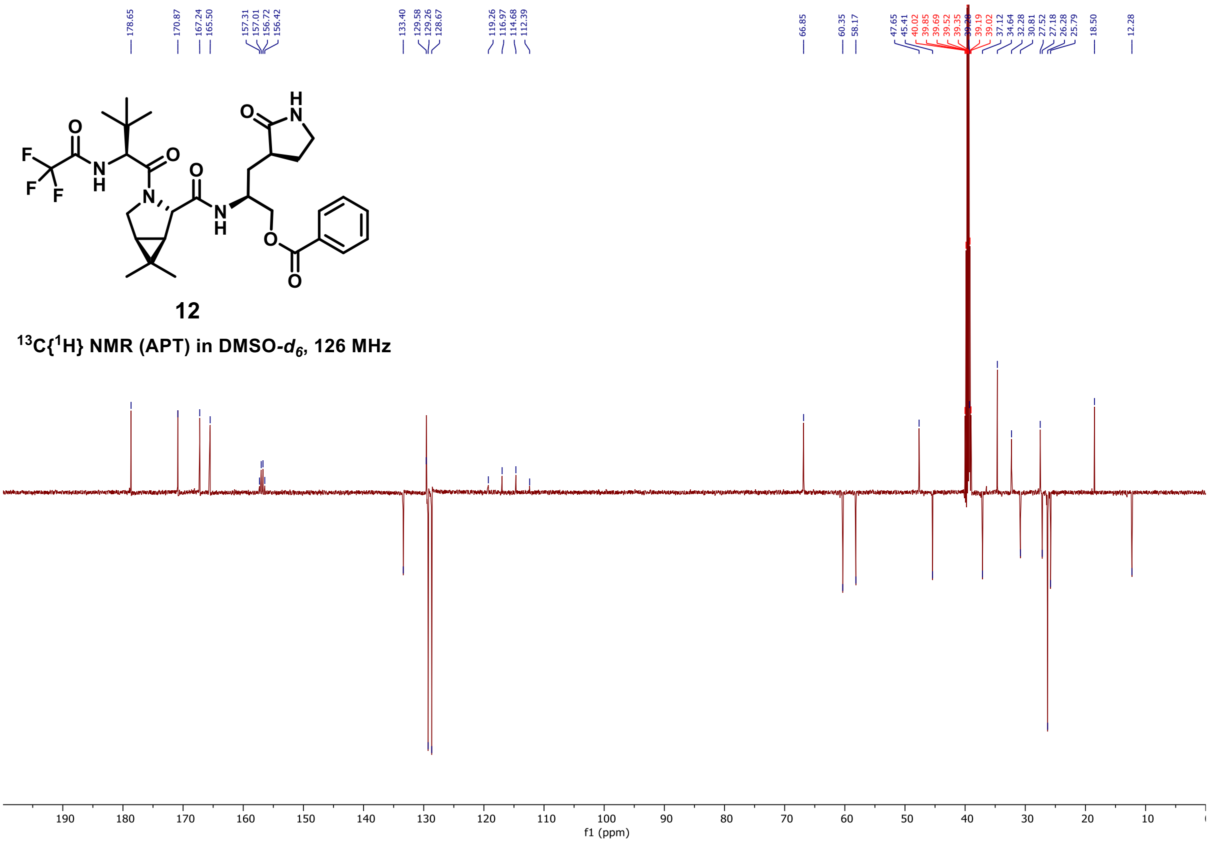
12
13C{1H} NMR (APT) in DMSO-d6, 126 MHz
f1 (ppm)

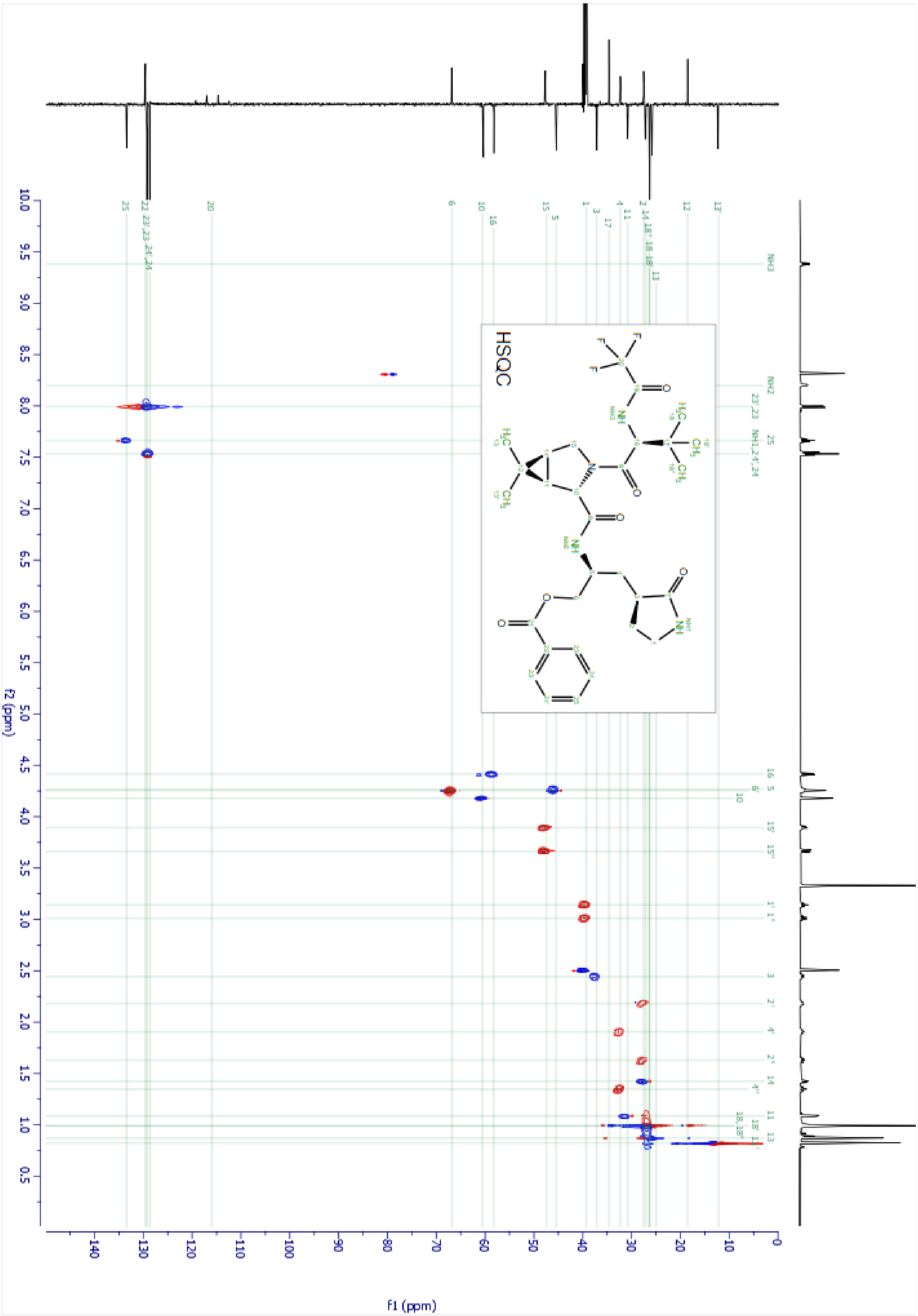
HSQC
f1 (ppm)
f2 (ppm)

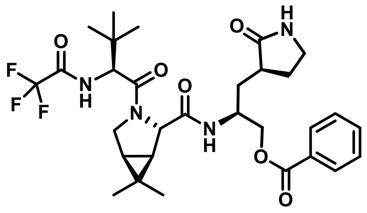

12

^{19}F NMR in DMSO-d_6, 470 MHz

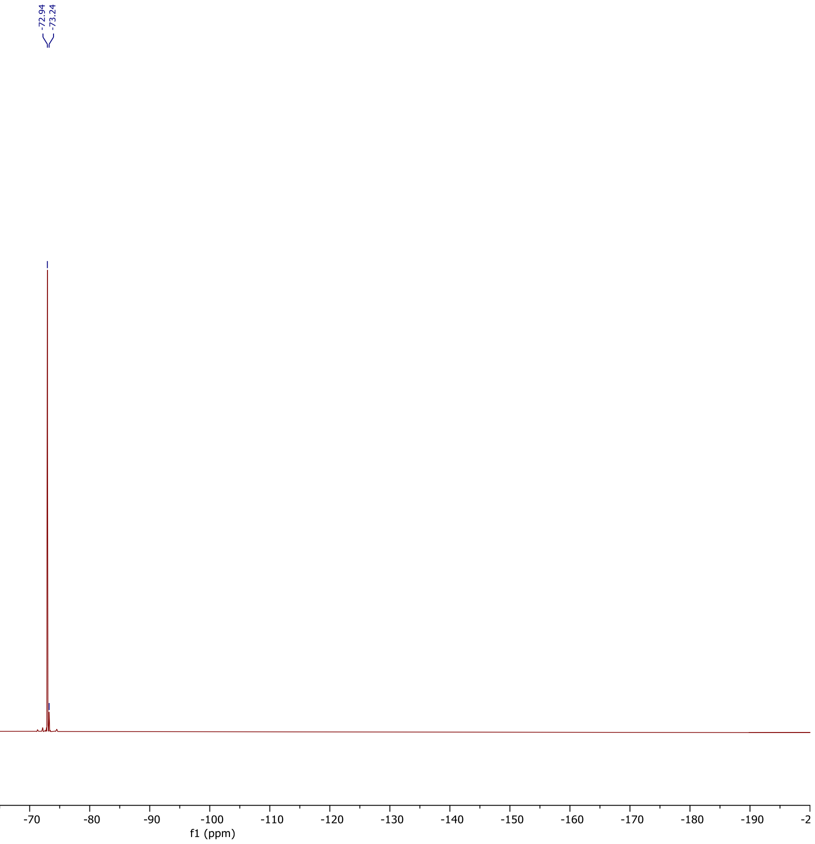

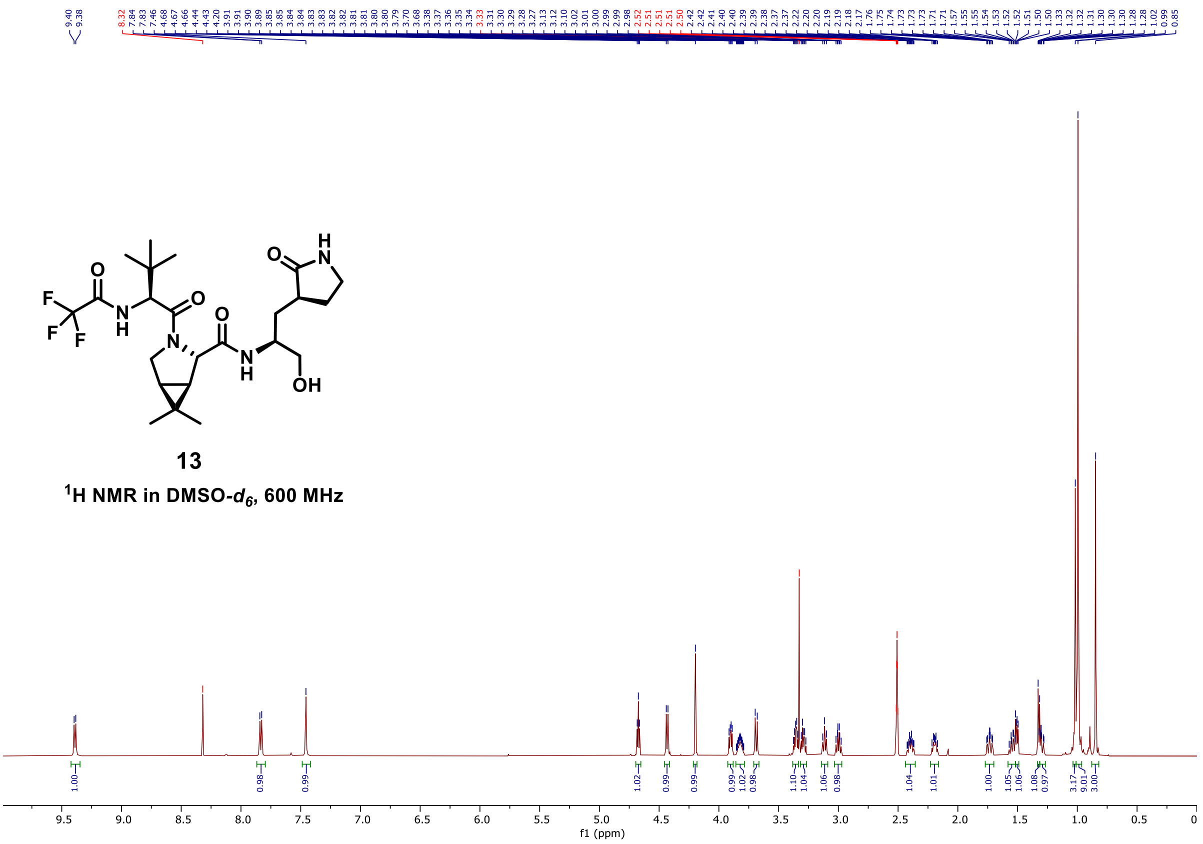

13

^{1}H NMR in DMSO-d_6, 600 MHz

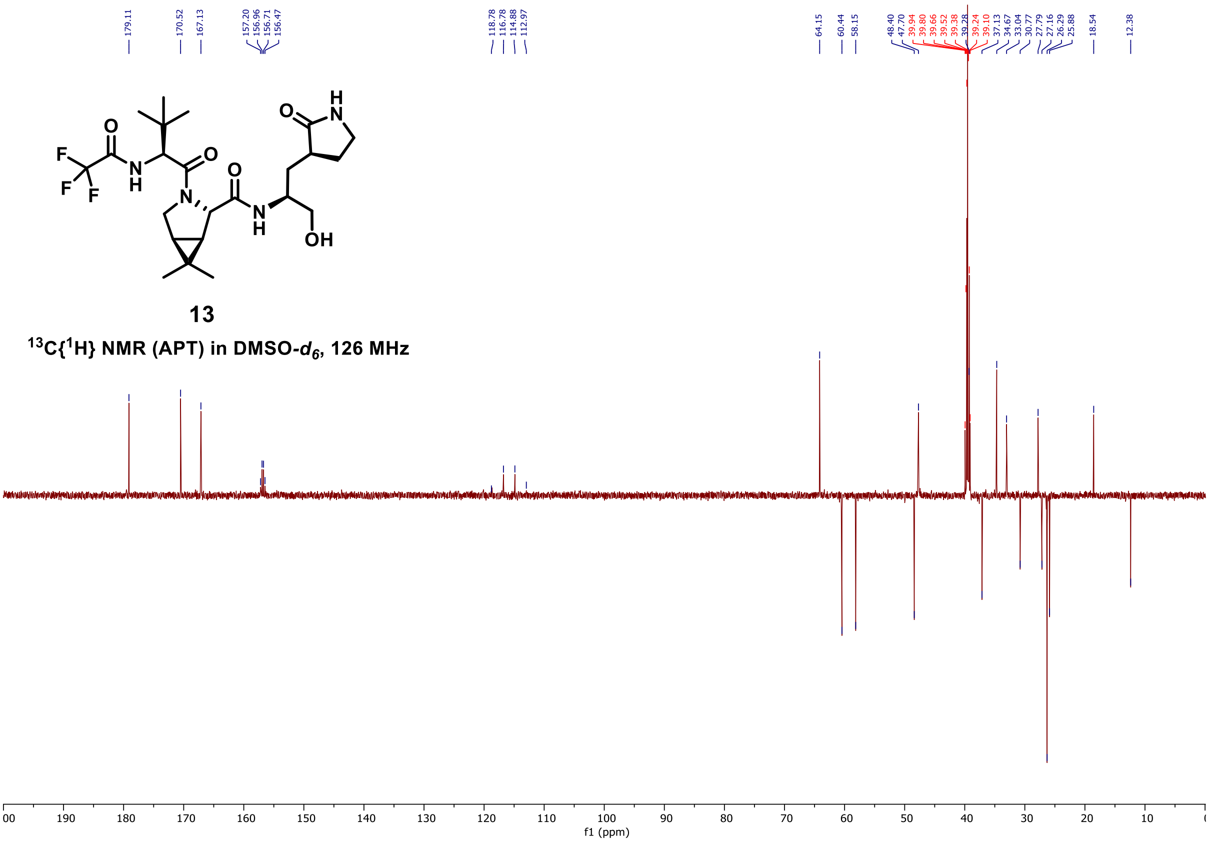

13

^{13}C{^{1}H} NMR (APT) in DMSO-d_6, 126 MHz

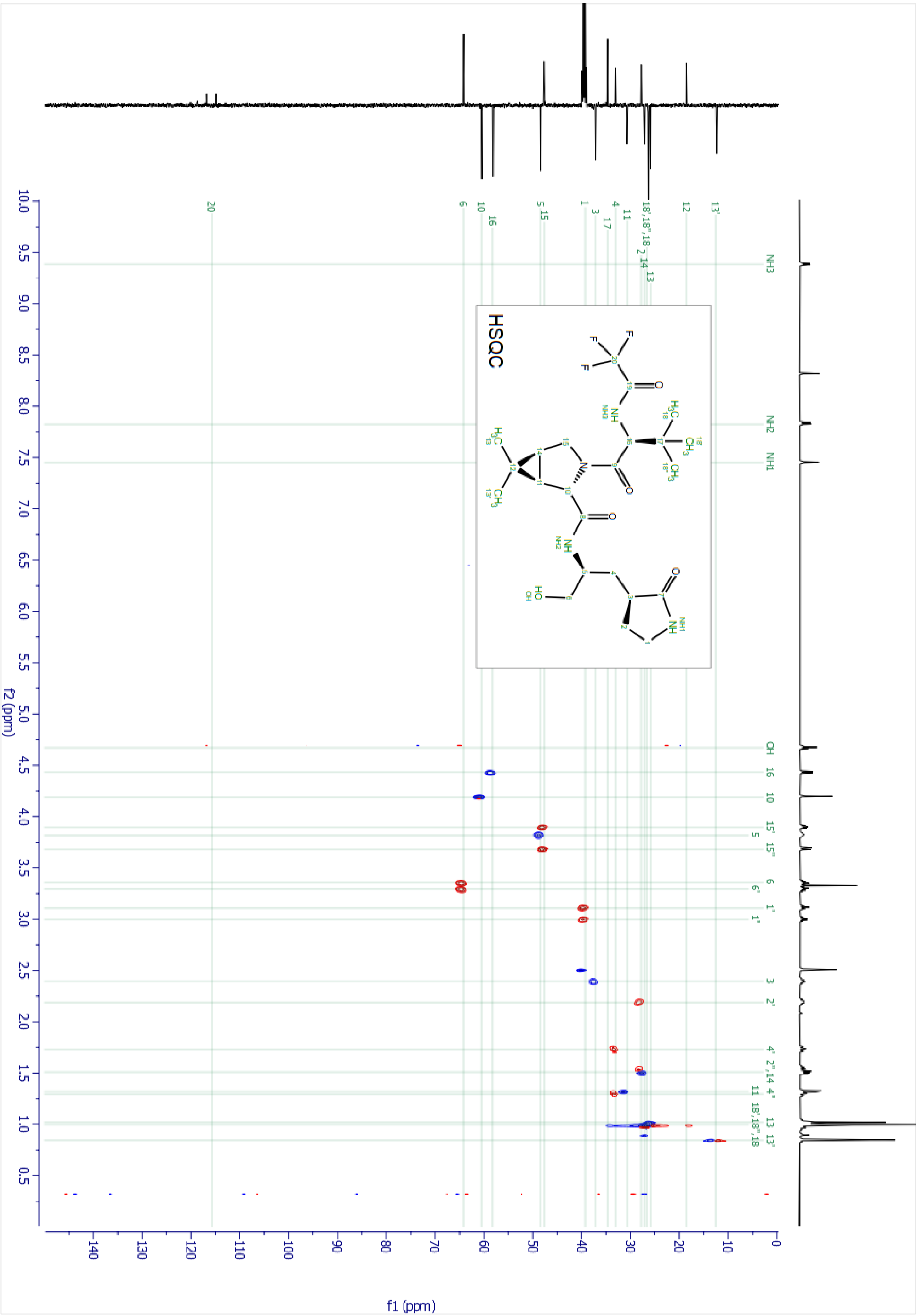
HSQC
f1 (ppm)
f2 (ppm)

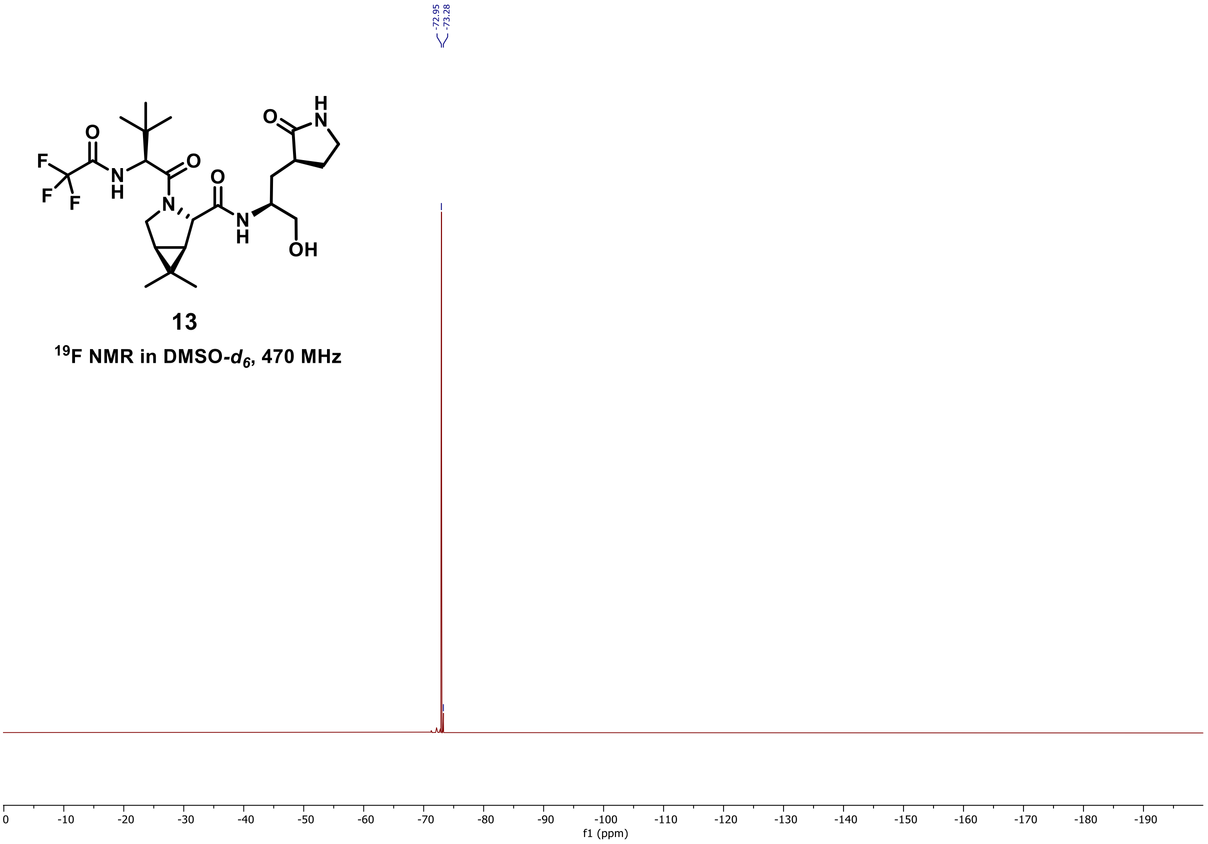

13

^{19}F NMR in DMSO-*d_6*, 470 MHz

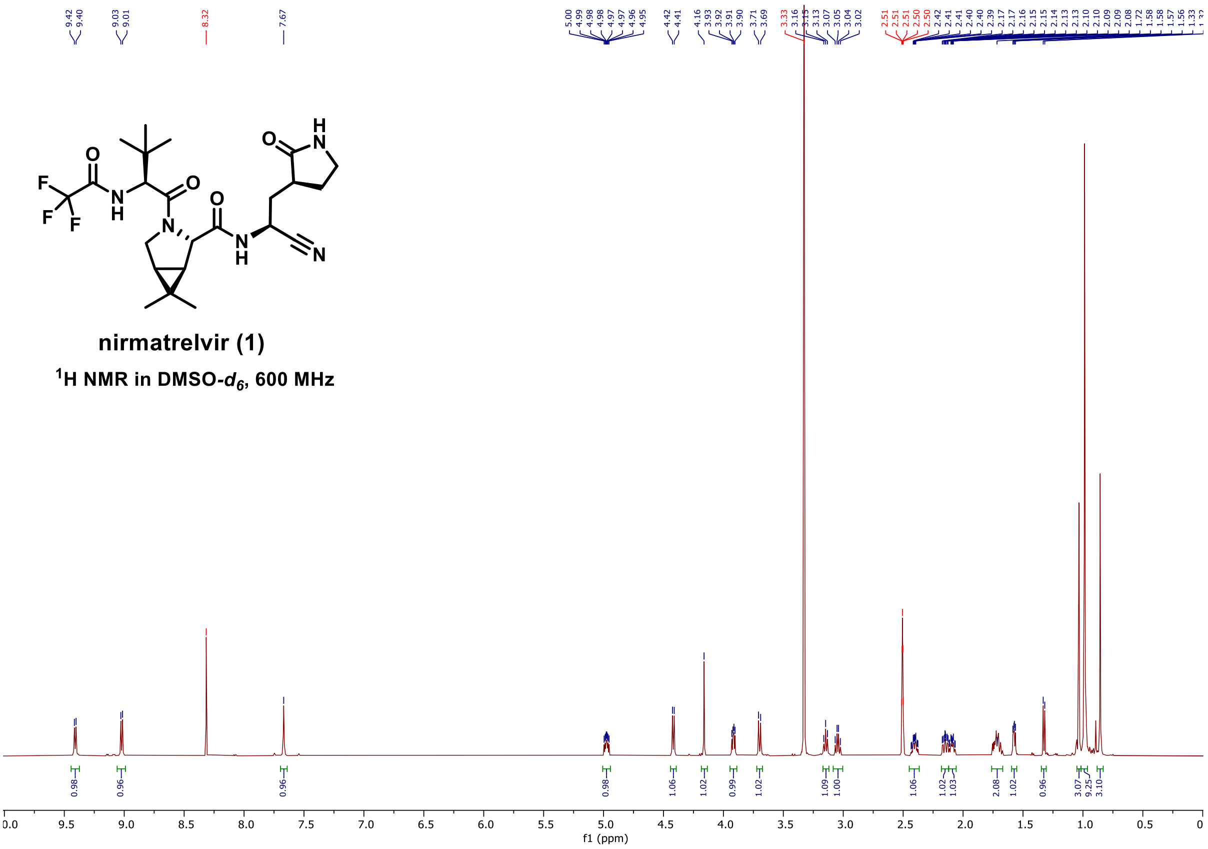
nirmatrelvir (1)
^{1}H NMR in DMSO-d_6, 600 MHz
f1 (ppm)

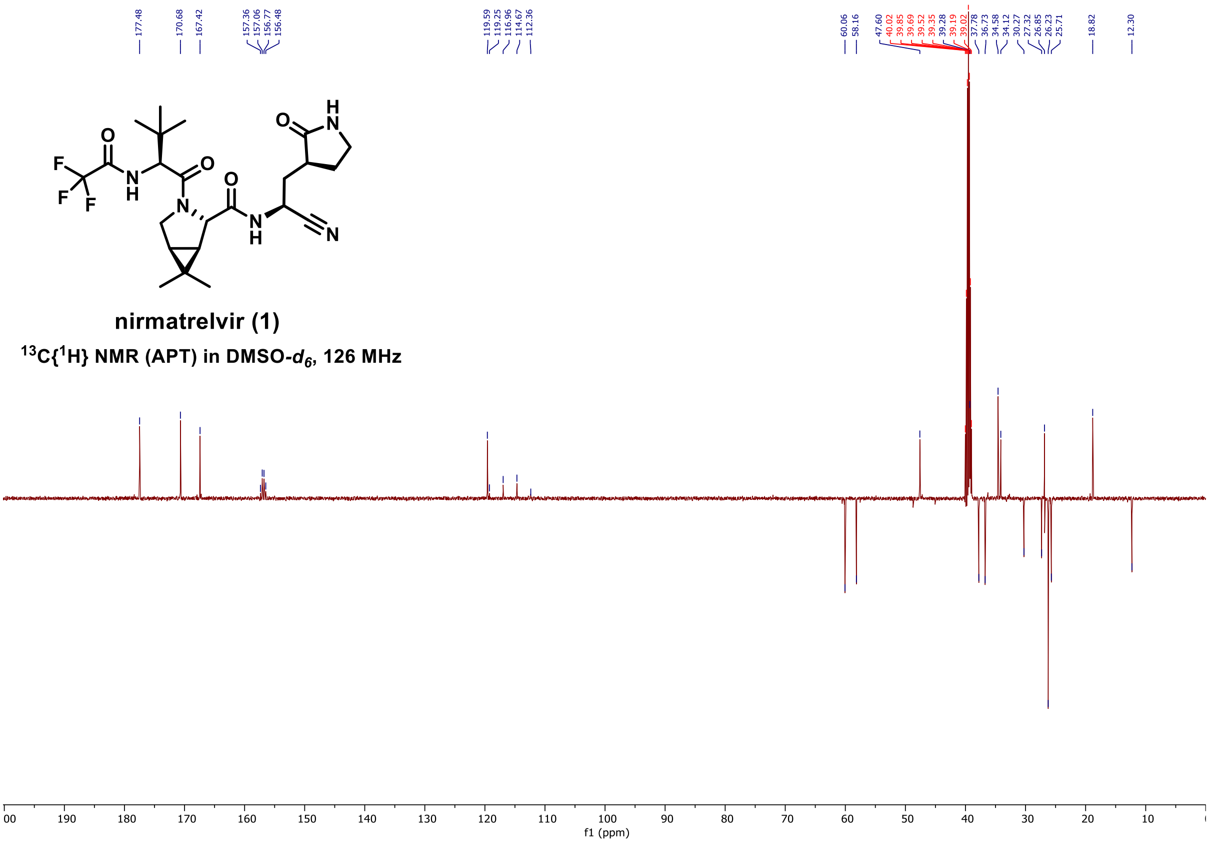
nirmatrelvir (1)
^{13}C{^{1}H} NMR (APT) in DMSO-d_6, 126 MHz
f1 (ppm)

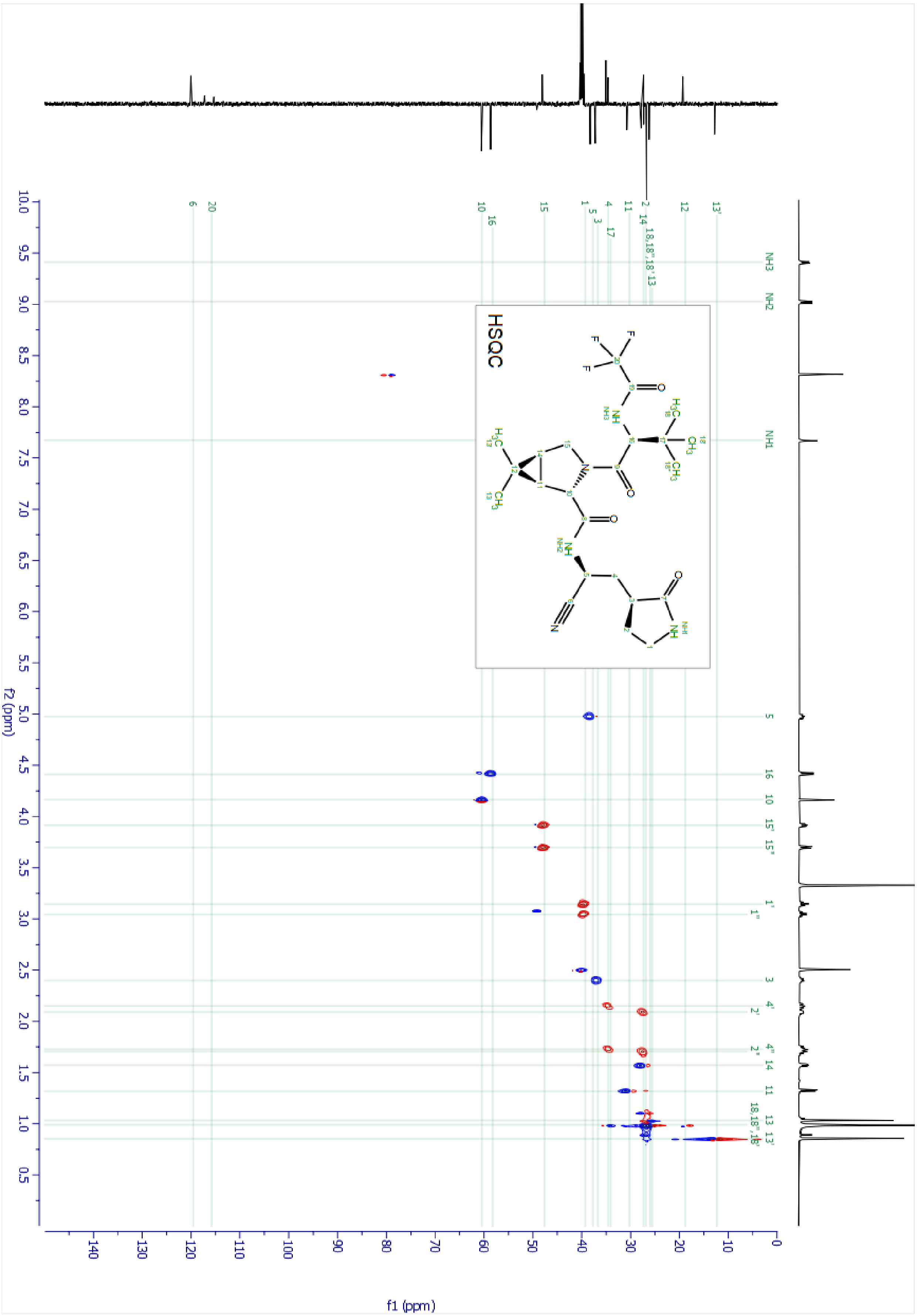
HSQC
f1 (ppm)
f2 (ppm)

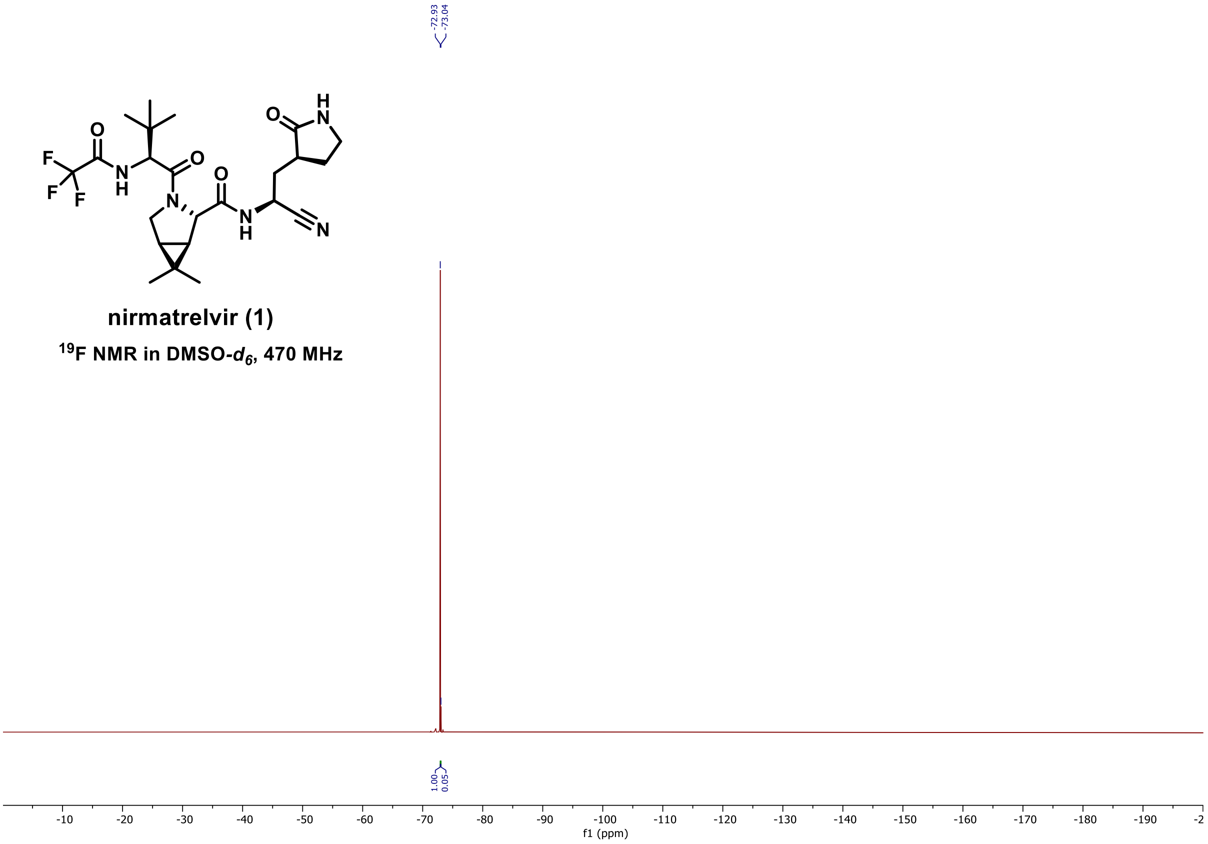

nirmatrelvir (1)

^{19}F NMR in DMSO-d_6, 470 MHz

HRMS

U3CR adduct **12**

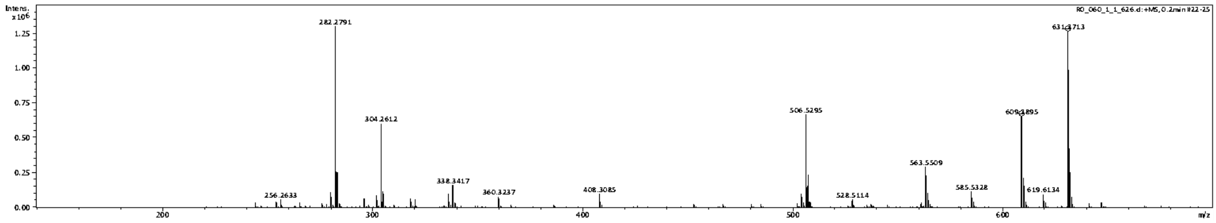

Meas. m/z	#	Ion Formula	m/z	err [ppm]	mSigma	# mSigma	Score	rdb	e^- Conf	N-Rule
609.2895	1	$C_{30}H_{40}N_4O_6F_3$	609.2894	**0.0**	12.0	1	100.00	12.0	even	ok
631.2714	1	$C_{30}H_{39}N_4O_6F_3Na$	631.2701	**2.1**	16.3	1	100.00	12.0	even	ok

Alcohol **13**

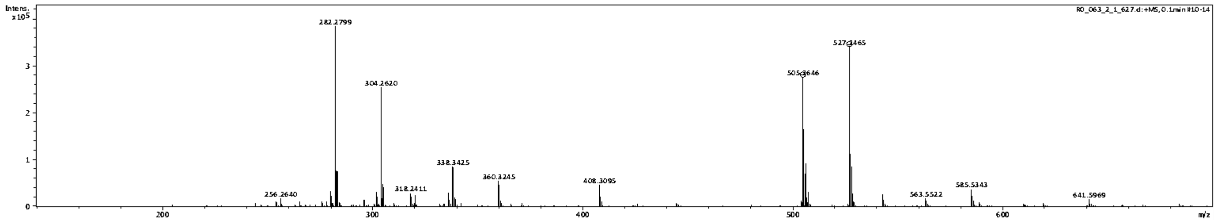

Meas. m/z	#	Ion Formula	m/z	err [ppm]	mSigma	# mSigma	Score	rdb	e^- Conf	N-Rule
505.2646	1	$C_{23}H_{36}N_4O_5F_3$	505.2632	**-2.9**	10.1	1	100.00	7.0	even	ok
527.2465	1	$C_{23}H_{35}N_4O_5F_3Na$	505.2652	**-2.5**	9.9	1	100.00	7.0	even	ok

Nirmatrelvir – **1**

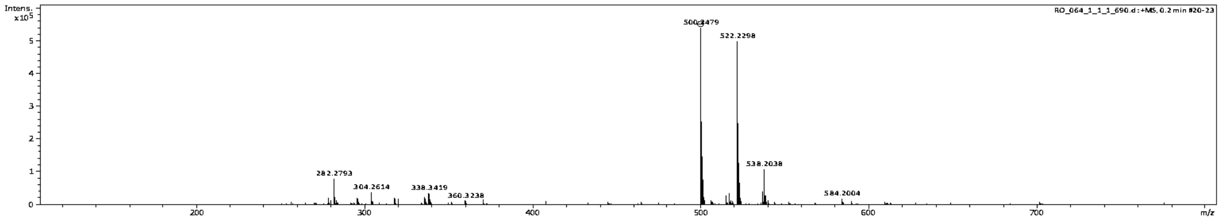

Meas. m/z	#	Ion Formula	m/z	err [ppm]	mSigma	# mSigma	Score	rdb	e^- Conf	N-Rule
500.2479	1	$C_{23}H_{36}N_4O_5F_3$	500.2479	**0.1**	1.2	1	100.00	9.0	even	ok
522.2298	1	$C_{23}H_{35}N_4O_5F_3Na$	505.2299	**0.1**	8.7	1	100.00	9.0	even	ok

SFC-MS

U3CR adduct **12**: Cellulose_1, 2-30% MeOH, 7 min. run time 2mL/min, PDA 224 nm Abs

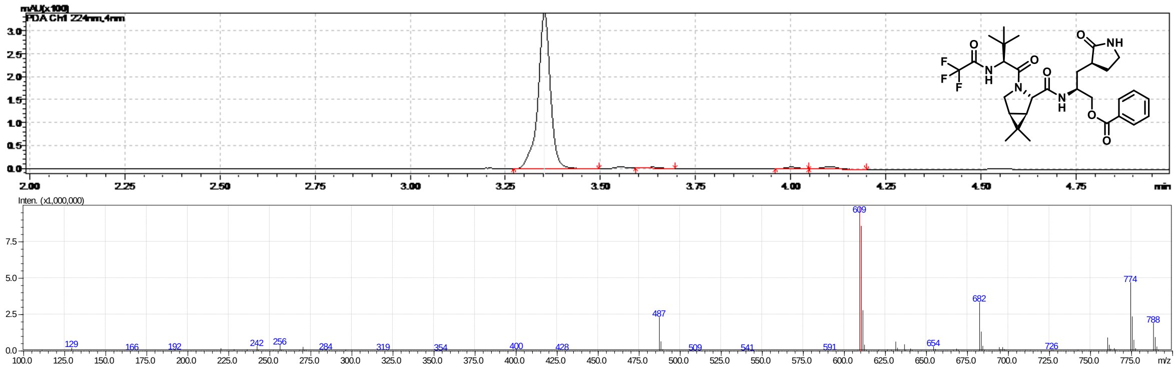

m/z [M+H] calc. =

Peak#	ret. time	area	area%
1	3.353	767421	**96.245**
2	3.638-4.103	29870	3.757
total:		797291	100.000

Alcohol **13**: Cellulose_1, 2-30% MeOH, 7 min. run time 2mL/min, PDA 224 nm Abs

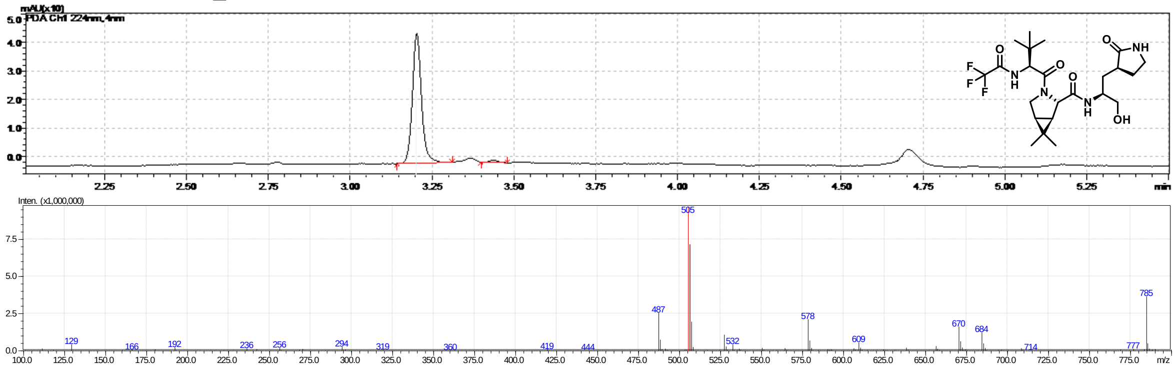

m/z [M+H] calc. =

Peak#	ret. time	area	area%
1	3.204	87.897	**98.663**
2	3.440	1.191	1.337
total:		89.088	100.000

Nirmatrelvir - **1**: Cellulose_3, 2-30% MeOH, 7 min. run time 2mL/min, PDA 224 nm Abs

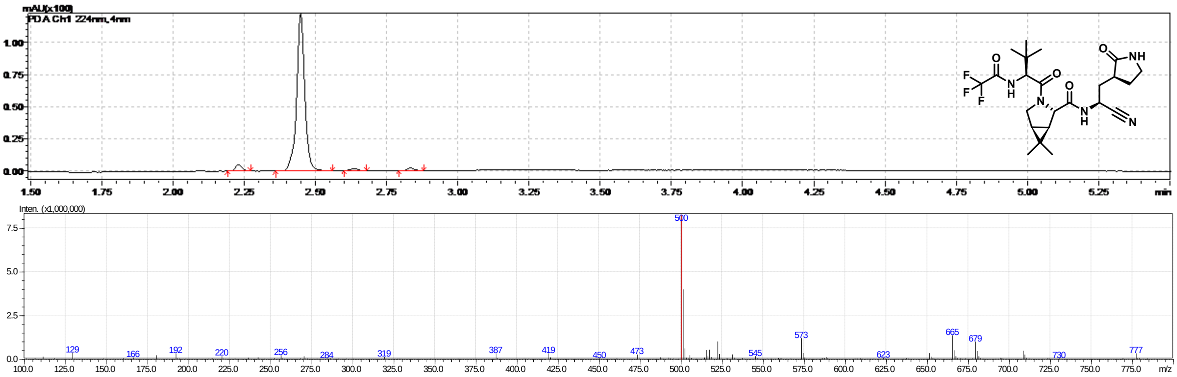

m/z [M+H] calc. =

Peak#	ret. time	area	area%
1	2.448	242.153	**97.234**
2	2.637-2.833	6.890	2.766
total:		249.042	100.000

References

1 Owen, D. R.; Allerton, C. M. N.; Anderson, A. S.; Aschenbrenner, L.; Avery, M.; Ber-ritt, S.; Boras, B.; Cardin, R. D.; Carlo, A.; Coffman, K. J.; Dantonio, A.; Di, L.; Eng, H.; Ferre, R.; Gajiwala, K. S.; Gibson, S. A.; Greasley, S. E.; Hurst, B. L.; Kadar, E. P.; Kalgutkar, A. S.; Lee, J. C.; Lee, J.; Liu, W.; Mason, S. W.; Noell, S.; Novak, J. J.; Obach, R. S.; Ogilvie, K.; Patel, C.; Pettersson, M.; Rai, D. K.; Reese, M. R.; Sammons, M. F.; Sathish, J. G.; Singh, R. S. P.; Steppan, C. M.; Stewart, A. E.; Tuttle, J. B.; Updyke, L.; Verhoest, P. R.; Wei, L.; Yang, Q.; Zhu, Y. An oral SARS-CoV-2 M^{pro} inhibitor clinical candidate for the treatment of COVID-19. *Science* **2021**, *374*, 1586–1593.

2 Li, T.; Liang, J.; Ambrogelly, A.; Brennan, T.; Gloor, G.; Huisman, G.; Lalonde, J.; Lekhal, A.; Mijts, B.; Muley, S.; Newman, L.; Tobin, M.; Wong, G.; Zaks, A.; Zhang, X. Efficient, Chemoenzymatic Process for Manufacture of the Boceprevir Bicyclic [3.1.0]Proline Intermediate Based on Amine Oxidase-Catalyzed Desymmetrization. *J. Am. Chem. Soc.* **2012**, *134*, 6467–6472.

3 Köhler, V.; Bailey, K. R.; Znabet, A.; Raftery, J.; Helliwell, M.; Turner, N. J. Enantioselective Biocatalytic Oxidative Desymmetrization of Substituted Pyrrolidines. *Angew. Chem. Int. Ed.* **2010**, *49*, 2182-2184.

4 Meudt, A.; Scherer, S.; Nerdinger, S. Method for producing nitriles and isonitriles by using dehydration reactors with propanephosphonic acid anhydrides. Eur. Pat. Appl. EP1713763A1, **2006**.

5 Creedon, S. M.; Crowley, H. K.; McCarthy, D. G. Dehydration of formamides using the Burgess reagent: a new route to isocyanides. *J. Chem. Soc. Perkin Trans. 1* **1998**, 1015–1017.

6 Kobayashi, G.; Saito, T.; Kitano, Y. A novel method for preparing isocyanides from *N*-substituted formamides with chlorophosphate compounds. *Synthesis* **2011**, *20*, 3225–3234.

7 Waibel, K. A.; Nickisch, R.; Möhl, N.; Seim, R.; Meier, M. A. R. A more sustainable and highly practicable synthesis of aliphatic isocyanides. *Green Chem.* **2020**, *22*, 933–941.

8 Sheldrick, G. M. Crystal Structure Refinement with SHELXL. *Acta Cryst.* **2015**, *C71*, 3–8.

9 Sheldrick, G. M. SHELXT – Integrated Space-Group and Crystal-Structure Determination. *Acta Cryst.* **2015**, *A71*, 3–8.

10 Spek, A. L. Single-Crystal Structure Validation with the Program PLATON. *J. Appl. Cryst.* **2003**, *36*, 7–13.

11 Spek, A. L. Structure Validation in Chemical Crystallography. *Acta Cryst.* **2009**, *D65*, 148–155.

12 Flack, H. D. On Enantiomorph‐polarity Estimation. *Acta Cryst.* **1983**, *A39*, 876–881.
