# Supplementary figures and images for: Multicomponent Synthesis of the SARS-CoV-2 Main Protease Inhibitor Nirmatrelvir
Source: J Org Chem. 2023 Aug 22;88(17):12565–71. doi: 10.1021/acs.joc.3c01274 (PMC10476182; doi:10.1021/acs.joc.3c01274)

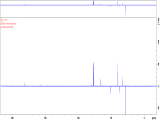

Supplement: Supplementary file 2 — jo3c01274_si_002.zip [file jo3c01274_si_002.zip › 1_NHBoc amino alcohol (9)/9_13C-NMR/pdata/1/thumb.png]

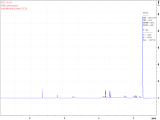

Supplement: Supplementary file 2 — jo3c01274_si_002.zip [file jo3c01274_si_002.zip › 1_NHBoc amino alcohol (9)/9_1H-NMR/pdata/1/thumb.png]

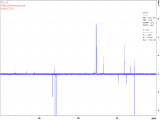

Supplement: Supplementary file 2 — jo3c01274_si_002.zip [file jo3c01274_si_002.zip › 2_NHBoc amino benzoate (10)/10_13C-NMR/pdata/1/thumb.png]

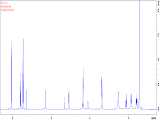

Supplement: Supplementary file 2 — jo3c01274_si_002.zip [file jo3c01274_si_002.zip › 2_NHBoc amino benzoate (10)/10_1H-NMR/pdata/1/thumb.png]

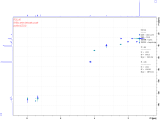

Supplement: Supplementary file 2 — jo3c01274_si_002.zip [file jo3c01274_si_002.zip › 2_NHBoc amino benzoate (10)/10_HSQC/pdata/1/thumb.png]

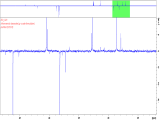

Supplement: Supplementary file 2 — jo3c01274_si_002.zip [file jo3c01274_si_002.zip › 3_Formamide (11)/11_13C-NMR/pdata/1/thumb.png]
