# Supplementary material for: Multicomponent Synthesis of the SARS-CoV-2 Main Protease Inhibitor Nirmatrelvir
Source: J Org Chem. 2023 Aug 22;88(17):12565–71. doi: 10.1021/acs.joc.3c01274 (PMC10476182; doi:10.1021/acs.joc.3c01274)
Supplement: Supplementary file 2 — jo3c01274_si_002.zip [file jo3c01274_si_002.zip › 3_Formamide (11)/11_HSQC/pdata/1/email_RO_023_7_1.pdf]

RO_023
b-formamido bezoate (rerun)
purified (CDCl3)

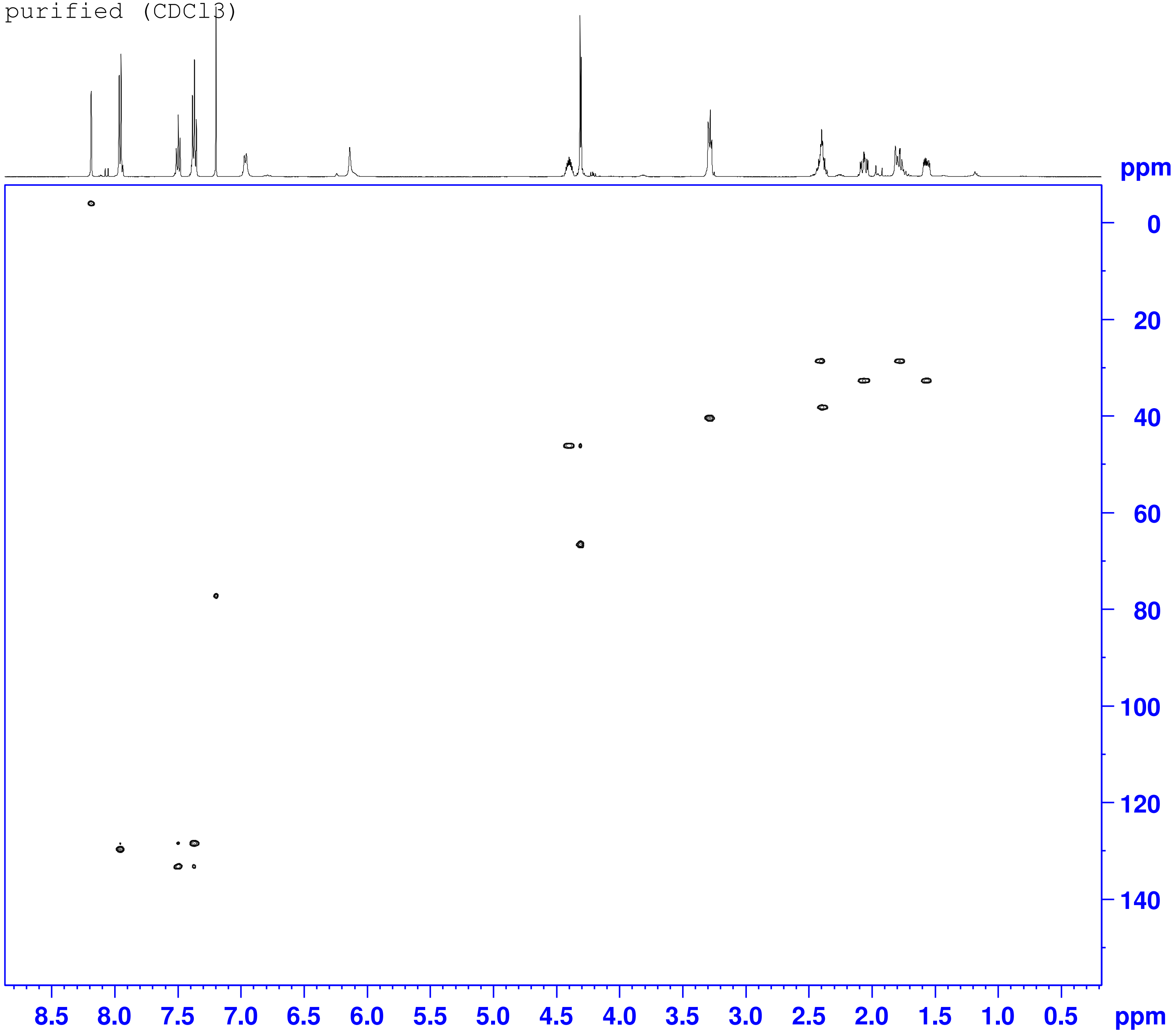


```
Current Data Parameters
NAME                RO_023
EXPNO                    7
PROCNO                   1

F2 - Acquisition Parameters
Date_             20220218
Time                 15.42
INSTRUM              spect
PROBHD   5 mm PABBO BB/
PULPROG      hsqcetgpsi2
TD                    1024
SOLVENT              CDCl3
NS                       2
DS                      16
SWH               4347.826 Hz
FIDRES            4.245924 Hz
AQ               0.1177600 sec
RG                    2050
DW                 115.000 usec
DE                    6.50 usec
TE                   295.3 K
CNST2        145.0000000
D0            0.00000300 sec
D1            1.45904005 sec
D4            0.00172414 sec
D11           0.03000000 sec
D16           0.00020000 sec
D24           0.00086207 sec
IN0           0.00002400 sec
ZGOPTNS

======== CHANNEL f1 ========
SFO1        500.2023124 MHz
NUC1                 1H
P1                12.40 usec
P2                24.80 usec
P28             1000.00 usec
PLW1        22.00000000 W

======== CHANNEL f2 ========
SFO2        125.7848205 MHz
NUC2                13C
CPDPRG[2           garp
P3                12.00 usec
P4                24.00 usec
PCPD2             70.00 usec
PLW2        95.00000000 W
PLW12        2.79180002 W

====== GRADIENT CHANNEL =====
GPNAM[1]      SMSQ10.100
GPNAM[2]      SMSQ10.100
GPNAM[3]      SMSQ10.100
GPNAM[4]      SMSQ10.100
GPZ1              80.00 %
GPZ2              20.10 %
GPZ3              11.00 %
GPZ4              -5.00 %
P16             1000.00 usec
P19              600.00 usec

F1 - Acquisition parameters
TD                   256
SFO1            125.7848 MHz
FIDRES         81.380211 Hz
SW               165.627 ppm
FnMODE      Echo-Antiecho

F2 - Processing parameters
SI                  1024
SF           500.2000480 MHz
WDW                QSINE
SSB                    2
LB       0 Hz
GB       0
PC                  1.40

F1 - Processing parameters
SI                  1024
MC2          echo-antiecho
SF           125.7753900 MHz
WDW                QSINE
SSB                    2
LB       0 Hz
GB       0
```
