# Supplementary material for: Multicomponent Synthesis of the SARS-CoV-2 Main Protease Inhibitor Nirmatrelvir
Source: J Org Chem. 2023 Aug 22;88(17):12565–71. doi: 10.1021/acs.joc.3c01274 (PMC10476182; doi:10.1021/acs.joc.3c01274)
Supplement: Supplementary file 2 — jo3c01274_si_002.zip [file jo3c01274_si_002.zip › 5_U3CR adduct (12)/12_19F/pdata/1/email_KH_006_5_1.pdf]

KH_006
U-3CR 13C APT
purified (DMSO)

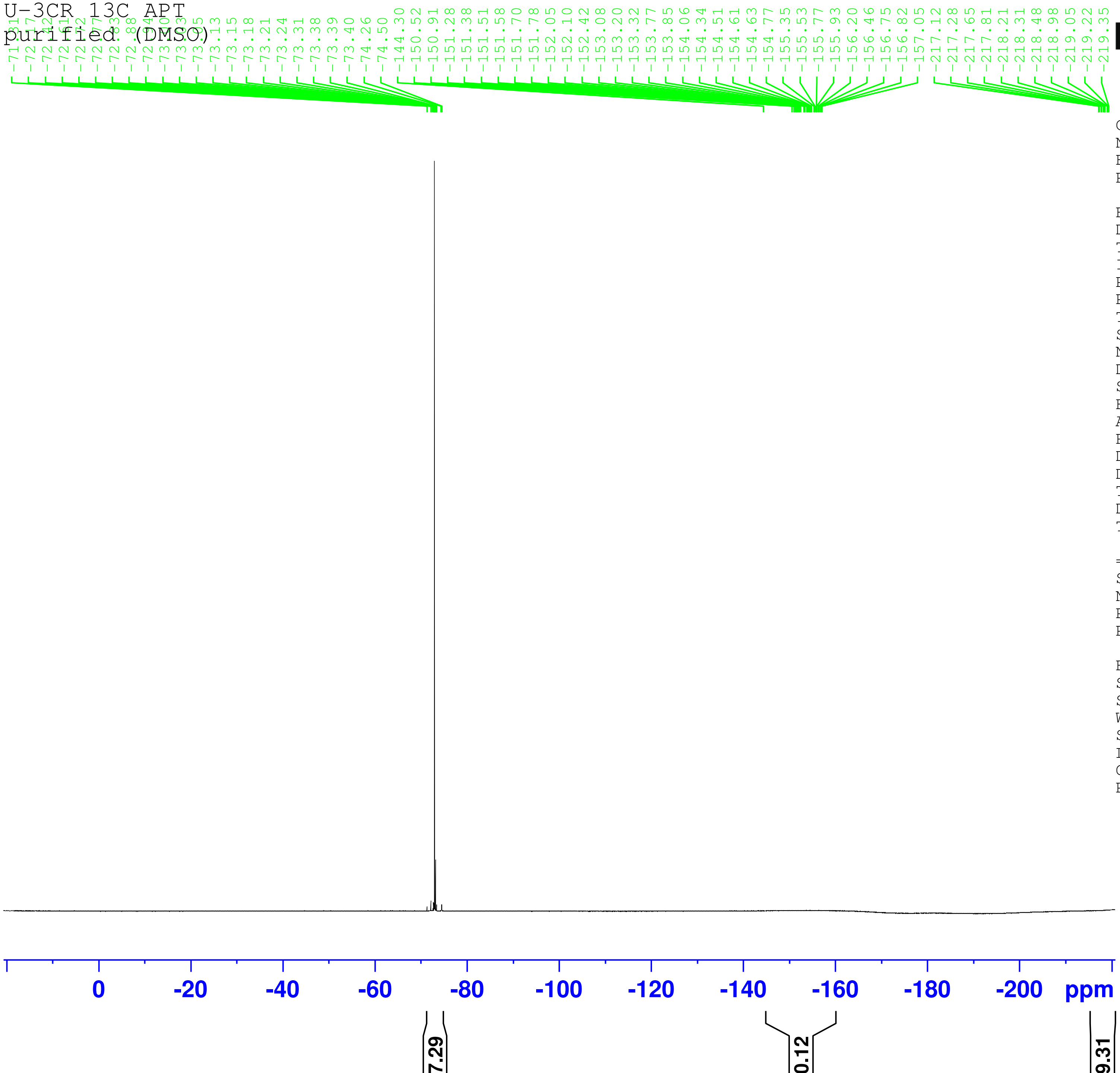

Current Data Parameters

NAME	KH_006
EXPNO	5
PROCNO	1

F2 - Acquisition Parameters

Date_	20221109
Time	0.13
INSTRUM	spect
PROBHD	5 mm PABBO BB/
PULPROG	zgflqn
TD	131072
SOLVENT	DMSO
NS	16
DS	4
SWH	113636.367 Hz
FIDRES	0.866977 Hz
AQ	0.5767168 sec
RG	322
DW	4.400 usec
DE	6.50 usec
TE	298.3 K
D1	1.00000000 sec
TD0	1

======== CHANNEL f1 ========

SFO1	470.6111772 MHz
NUC1	19F
P1	15.00 usec
PLW1	43.00000000 W

F2 - Processing parameters

SI	65536
SF	470.6582430 MHz
WDW	EM
SSB	0
LB	0.30 Hz
GB	0
PC	1.00
