# Supplementary material for: Multicomponent Synthesis of the SARS-CoV-2 Main Protease Inhibitor Nirmatrelvir
Source: J Org Chem. 2023 Aug 22;88(17):12565–71. doi: 10.1021/acs.joc.3c01274 (PMC10476182; doi:10.1021/acs.joc.3c01274)
Supplement: Supplementary file 2 — jo3c01274_si_002.zip [file jo3c01274_si_002.zip › 7_Nirmatrelvir (1)/1_13C-NMR/pdata/1/email_KH_006_8_1.pdf]

KH_009
Nirmatrelvir 13C APT
purified (DMSO)

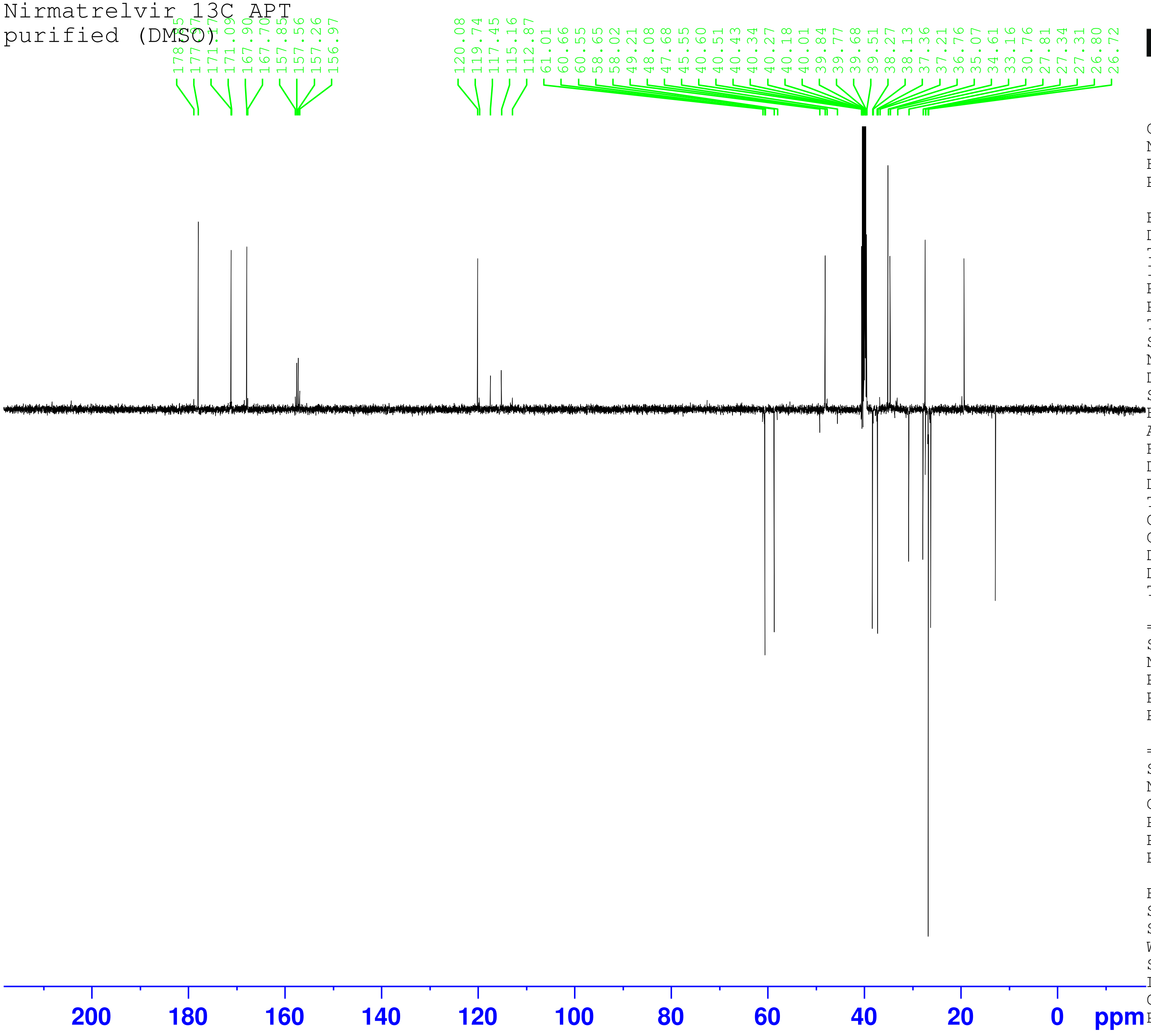

BRUKER

Current Data Parameters
NAME KH_006
EXPNO 8
PROCNO 1

F2 - Acquisition Parameters
Date_ 20221109
Time 4.51
INSTRUM spect
PROBHD 5 mm PABBO BB/
PULPROG jmod
TD 65536
SOLVENT DMSO
NS 2560
DS 4
SWH 29761.904 Hz
FIDRES 0.454131 Hz
AQ 1.1010048 sec
RG 2050
DW 16.800 usec
DE 6.50 usec
TE 298.2 K
CNST2 145.0000000
CNST11 1.0000000
D1 2.00000000 sec
D20 0.00689655 sec
TD0 1

======== CHANNEL f1 ========
SFO1 125.7879676 MHz
NUC1 13C
P1 11.00 usec
P2 22.00 usec
PLW1 110.00000000 W

======== CHANNEL f2 ========
SFO2 500.2020008 MHz
NUC2 1H
CPDPRG[2 waltz16
PCPD2 80.00 usec
PLW2 4.38959980 W
PLW12 0.50000000 W

F2 - Processing parameters
SI 32768
SF 125.7753900 MHz
WDW EM
SSB 0
LB 1.00 Hz
GB 0
PC 1.40
