# Supplementary material for: Multicomponent Synthesis of the SARS-CoV-2 Main Protease Inhibitor Nirmatrelvir
Source: J Org Chem. 2023 Aug 22;88(17):12565–71. doi: 10.1021/acs.joc.3c01274 (PMC10476182; doi:10.1021/acs.joc.3c01274)
Supplement: Supplementary file 2 — jo3c01274_si_002.zip [file jo3c01274_si_002.zip › 7_Nirmatrelvir (1)/1_19F/pdata/1/email_KH_006_7_1.pdf]

KH_009
Nirmatrelvir 13C APT
purified (DMSO)

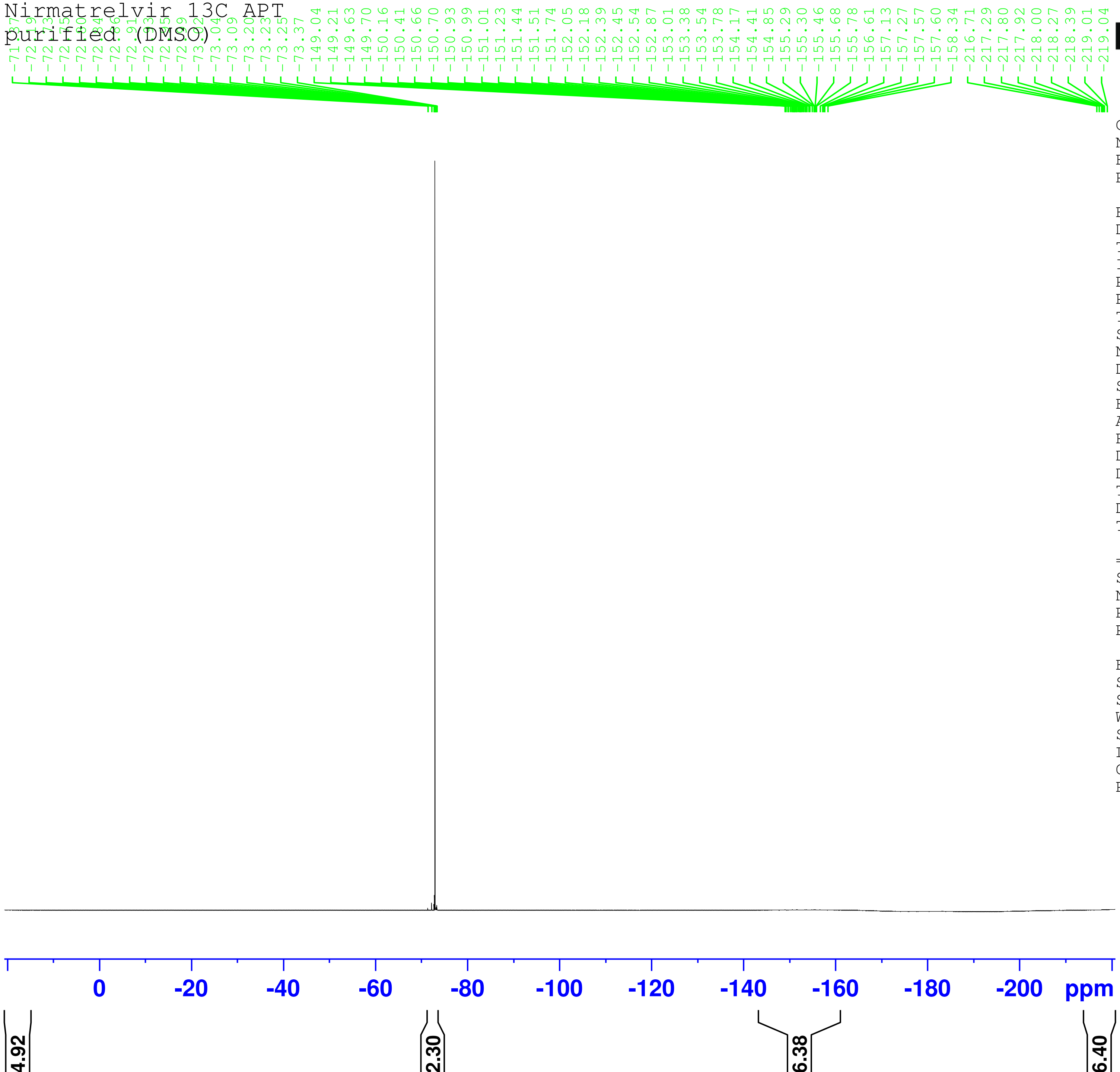

Current Data Parameters
NAME KH_006
EXPNO 7
PROCNO 1

F2 - Acquisition Parameters
Date_ 20221109
Time 11.03
INSTRUM spect
PROBHD 5 mm PABBO BB/
PULPROG zgflqn
TD 131072
SOLVENT DMSO
NS 16
DS 4
SWH 113636.367 Hz
FIDRES 0.866977 Hz
AQ 0.5767168 sec
RG 362
DW 4.400 usec
DE 6.50 usec
TE 298.2 K
D1 1.00000000 sec
TD0 1

======== CHANNEL f1 ========
SFO1 470.6111772 MHz
NUC1 19F
P1 15.00 usec
PLW1 43.00000000 W

F2 - Processing parameters
SI 65536
SF 470.6582430 MHz
WDW EM
SSB 0
LB 0.30 Hz
GB 0
PC 1.00
